# Manual for abdominal ultrasound in cancer screening and health checkups, revised edition (2021)

**DOI:** 10.1007/s10396-022-01272-w

**Published:** 2023-01-23

**Authors:** Shinji Okaniwa, Toshiko Hirai, Masahiro Ogawa, Sachiko Tanaka, Kazuo Inui, Takashi Wada, Naoki Matsumoto, Shigehiko Nishimura, Yuko Chiba, Hiroyoshi Onodera, Takashi Kumada, Masahisa Kojima, Michiko Nakajima, Yoshihiro Mizuma, Shinji Tanaka, Toru Nishikawa, Shuichi Mihara, Yoshioki Yoda, Masaki Adachi, Tomofumi Atarashi, Working Group on Revision of the Manual for Abdominal Ultrasound in Cancer Screening and Health Checkups, Ultrasound Screening Committee of the Japanese Society of Gastrointestinal Cancer Screening, Masayuki Kitano, Yutaka Chiba, Masahiko Nakata, Takashi Nishimura, Hiroshi Matsuo, Hideaki Mori, Noritaka Wakui, Kiyoka Sugita, Kenichi Maruyama, Kazuhiro Murakami, Akira Kuwajima, Ryuzo Sekiguchi, Satoru Ikeda, Yoshitomo Ishikawa, Kazuhiro Iwashita, Shinji Okaniwa, Masahiro Ogawa, Ayumi Oguri, Norikazu Obane, Mitsuko Kato, Masahiro Kaneko, Satoshi Kawabata, Kiyofumi Kawabata, Takako Kojima, Miho Saito, Hitomi Sasaki, Yoko Sugino, Asako Sugimoto, Seiichiro Suda, Ryosuke Taiji, Yu Tamura, Yuko Chiba, Mika Tojo, Ayaka Nakajima, Naomi Tanaka, Toru Nishikawa, Shigehiko Nishimura, Katsuyoshi Hayashi, Takeshi Hisa, Toshiko Hirai, Midori Hirayama, Megumi Honma, Nagaaki Marugami, Michie Miura, Masanori Miyashita, Yukiko Morimoto, Satoshi Yamauchi, Toshiki Yamamoto, Hiroka Yoshinaga, Satoshi Wakasugi, Noriko Watanabe, Yukinobu Watanabe

**Affiliations:** 1 Department of Gastroenterology, Iida Municipal Hospital, Iida, Nagano Japan; 2 Department of General Diagnostic Imaging Center, Nara Medical University, Nara, Japan; 3 Division of Gastroenterology and Hepatology, Department of Medicine, Nihon University School of Medicine, Tokyo, Japan; 4 Osaka Center for Cancer and Cardiovascular Disease Prevention, Osaka, Japan; 5 Department of Gastroenterology, Yamashita Hospital, Aichi, Japan; 6 Health Science, The Jikei University Graduate School of Medicine, Tokyo, Japan; 7 Department of Surgery, Sumitomo Hospital, Osaka, Japan; 8 Clinical Inspection Department, Hokkaido Industrial Health Management Fund, Hokkaido, Japan; 9 Cancer Detection Center, Miyagi Cancer Society, Miyagi, Japan; 10 Department of Nursing, Gifu Kyoritsu University, Gifu, Japan; 11 Medical Examination Center, Urasoe General Hospital, Okinawa, Japan; 12 Nakagaya Clinic, Nagano, Japan; 13 Department of Internal Medicine, Higashi Kobe Hospital, Hyogo, Japan; 14 Japanese Red Cross Society Kumamoto Health Care Center, Kumamoto, Japan; 15 Department of Clinical Laboratory, Fujita Health University Hospital, Aichi, Japan; 16 Mihara Life Care Clinic, Kumamoto, Japan; 17 Health Care Center, Yamanashi Koseiren Health Care Center, Yamanashi, Japan; 18 Health Management Center, Saitama Medical University Hospital, Saitama, Japan; 19 Medical Check-Up Center, JA Hokkaido P.W.F.A.C. Obihiro Kosei General Hospital, Hokkaido, Japan

**Keywords:** Abdominal ultrasonography, Cancer screening, Heath checkup, Ultrasound screening, Category, Ultrasound finding

## Introduction

This manual is a revised version of the first edition [1–3] published jointly by the Japanese Society of Gastrointestinal Cancer Screening (JSGCS), the Japan Society of Ultrasonics in Medicine (JSUM), and Japan Society of Ningen Dock (JSND) in 2014.

Abdominal ultrasonography is distinct in that it is used to examine many organs, and it is applicable various kinds of disease including malignant neoplasm. Moreover, a procedure for describing examination findings had not been standardized. Therefore, its objective accuracy and efficacy as a screening modality could not be evaluated. In addition, there were no clear rules regarding examination procedures at any of the societies. Against this backdrop, the Guideline for Abdominal Ultrasound Cancer Screening [4, 5]—which was comprised of practice standards aimed at improving the quality of abdominal ultrasound cancer screening, and assessment criteria to allow for accurate assessment as a cancer screening modality—was published in 2011, led by the JSGCS Ultrasonic Screening Committee (former Ultrasonography Working Group). The Abdominal Ultrasound Screening Assessment Manual was subsequently created through a joint effort by the above-mentioned three societies by partially revising the Guideline and adding items and assessments. The aim was to improve the quality of and homogenize abdominal ultrasound screening, standardize examination results, and evaluate accuracy and efficacy through the widespread adoption of the Guideline by creating a manual shared by the three societies.

This revision had been planned to take place about 5 years after the original publication. The manual was revised by working groups at each of the three societies, with the additional participation of the Japanese Society of Sonographers (JSS), Japan Society of Health Evaluation and Promotion (JSHEP), and Japanese Association for Cancer Detection and Diagnosis (JACDD) as observer societies, based on current experience. The manual was created with the following structure: practice standards and specific ultrasound findings and categories, tables with ultrasound findings in Report Forms and assessments presented 1:1 and cautionary notes, and representative ultrasound images of the respective findings.

It is a well-known fact that the accuracy of ultrasonography differs depending on the status of the device, examiner, and examinee. In modern medicine, standards are required in terms of ergonomics and device conditions (including periodic inspections) to improve examination quality; the practice standards in this manual were revised based on the guidelines of each society as well as other sources. Although evidence is lacking in some areas and they are not required, we hope that institutions will refer to this manual as a rule of thumb for the current examination environment.

The biggest shortcoming of ultrasonography is a lack of objectivity, which it true of not only ultrasound images but also ultrasound findings and assessments. When we consider such factors as examinee/examiner transfers and diversification of secondary examination facilities, we predict a dramatic improvement in objectivity with the permeation of this manual, and we look forward to its widespread adoption going forward.

## Practice standards

**Fig. 1:**
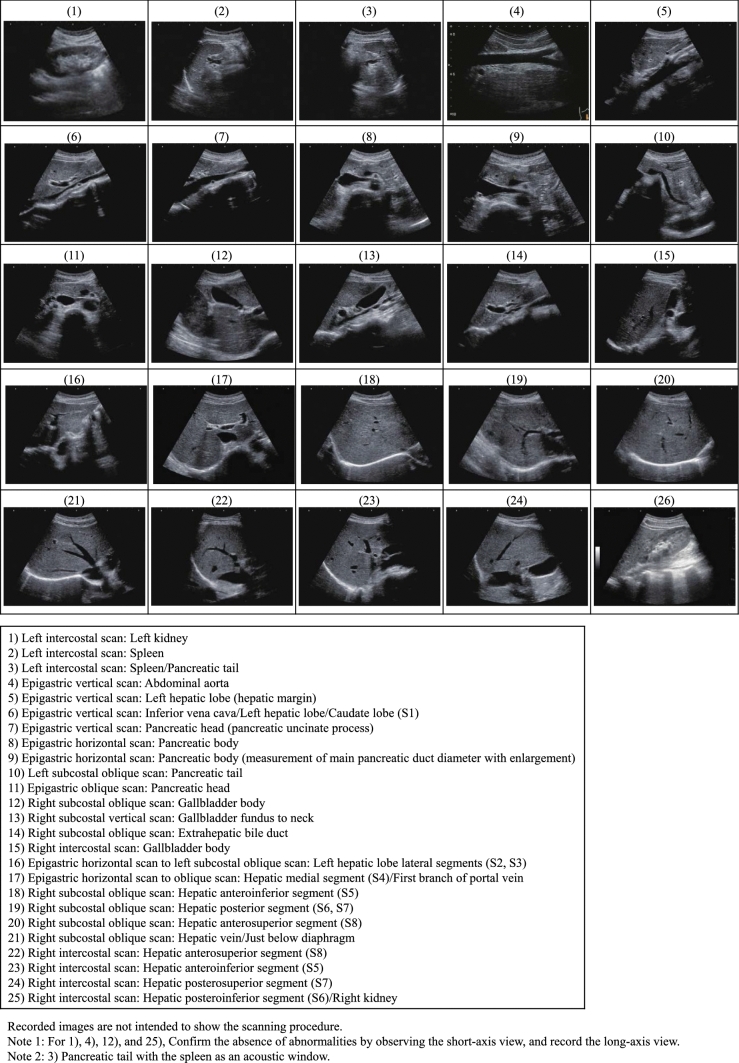
Twenty-five recommended sections to record

**Fig. 2:**
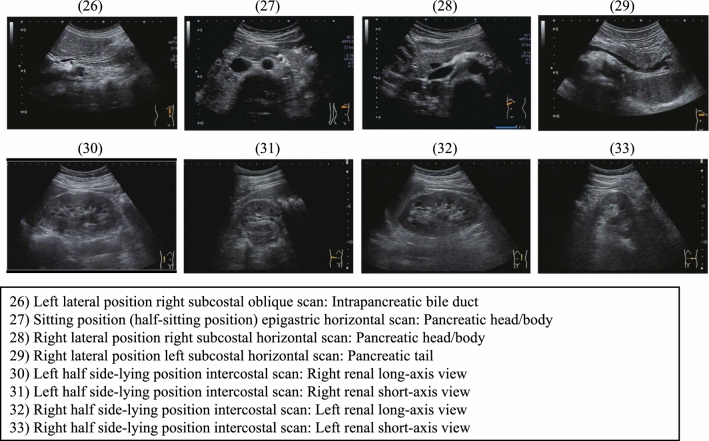
Images by position change

1. Before beginning an examination
  In terms of screening techniques performed primarily on people without symptoms, we have the "medical checkup," which is used to check and learn the level of an individual's health or check for risk of future disease, and "screening," the purpose of which is to check for the presence or absence of a specific disease. It should be noted that there are examinations where the two cannot be clearly distinguished based on the examination items [6]. Cancer screening, which is a typical screening, has two types: a population-based type carried out by municipalities and an opportunistic type carried out in other situations. In the case of population-based cancer screening, the Japanese government has proposed screening for five major cancers by modalities confirmed to lower the mortality rate. In addition to opportunistic cancer screening for hepatic cancer, renal cancer, and other cancers, this Abdominal Ultrasound Screening Assessment Manual also covers "screenings" to check for the presence or absence of disorders such as gallstones, as well as "medical checkups" to check for future disease risk such as hepatic steatosis and arteriosclerosis.
  There are merits and demerits with any cancer screening, not just ultrasonography, but the merits must outweigh the demerits. Therefore, the examiner needs to understand the merits and demerits of cancer screening and improve the quality of the screening, and the examiner must obtain proper informed consent from the examinee. It is also important to understand that there are two distinct reasons for performing a thorough examination in response to an abnormal finding detected by screening: one is to make a definitive diagnosis of cancer and the other is to rule out suspected cancer.
  Merits
  Early treatment based on early detection of cancer is possible.
  Psychological sense of relief when no abnormalities are found.
  Demerits
  Possibility of false-negative tests (ultrasonography does not detect 100% of cancers).
  Possibility of false-positive tests (as a result, there is a possibility of examinee complications associated with unnecessary invasive procedures and examinations, and being subjected to psychological and economic strain).
  Possibility of overdiagnosis (detection of cancer that does not affect the vital prognosis).
2. Target organs
  The target organs covered in this Manual are the liver, biliary tract, pancreas, spleen, kidneys, and abdominal aorta.
  Tell the examinee the target organs in advance, and explain that there may be some cases and sites where each organ may be difficult to observe.
  The adrenal gland and lower abdominal region (e.g., bladder, uterus, ovaries, and prostate) are not formally regarded as target organs, but any findings observed during the scanning process should be recorded. In addition, in cases where a target organ will be added with a voluntary contract with the client, clearly communicate the details of the examination to the examinee.
3. Examination environment
  1. Diagnostic unit
    Use a convex probe with a frequency of 3.5-7.0 MHz.
    Add other probes such as a high-frequency probe (7.5 MHz or higher), linear probe, or microconvex probe as necessary according to the examinee's situation.
    Use of a unit capable of tissue harmonic imaging and color Doppler is recommended.
    Periodically perform appropriate maintenance/management of the diagnostic unit, and avoid using a unit beyond its service life (normally 7 years).
  2. Operators
    A JSGCS gastrointestinal cancer screening general certified physician or gastrointestinal cancer screening certified physician (hepatobiliary system and pancreas), JSUM specialist or technician with an ultrasonographer qualification in the medical checkup domain or gastrointestinal domain certified by JSUM, Board Certified Physician of JSND, Health Evaluation and Promotion Specialist of JSHEP/JSND, Japan Radiological Society (JRS) radiology specialist, or Japanese Society of Laboratory Medicine (JSLM) clinical laboratory test specialist should be in charge of the examination.
  3. Advanced preparation
    Examinees with an examination scheduled for the morning should not eat solid foods or dairy products after 10 p.m. the night before.
    Examinees with an examination scheduled for the afternoon should not eat solid foods or dairy products within 6 h before the examination.
    Drinking liquids (e.g., cold/hot water) to prevent dehydration is allowed up to about 200 ml per time up to 2 h before the examination.
    In cases where a gastrointestinal tract examination will be performed the same day, perform ultrasonography first, with the exception of upper endoscopy using carbon dioxide [7].
  4. Peripheral facilities
    A proper examination environment is said to not only lessen examiner fatigue but also prevent misdiagnosis, making it an important factor.
    In addition to a private room to protect the examinee's privacy or equivalent examination room, there is an ergonomically recommended environment that encompasses everything including the heights of the examining table, chair, and monitor; therefore, one should aim to perform examinations under the correct environment (Refer to "Proposal from The Japan Society of Ultrasonics in Medicine for ultrasonographers to work safely, comfortably, and healthily: Equipment and work environment to prevent work-related musculoskeletal disorders and eye disorders" [8]).
  5. Precautions during epidemics
    The COVID-19 pandemic completely changed how screenings are performed. Examiners will need to familiarize themselves with ultrasonography-related precautions during epidemics in preparation for new infectious diseases in the future based on this experience.
    Ultrasonography has distinct examination procedures, sanitization procedures, and so forth that differ from those of other medical care.
    Take into account ultrasound screening methods during epidemics by referring to such sources as the Ultrasound Equipment and Safety Committee's "Recommendations and resources related to handling and safety of ultrasonic diagnostic equipment" [9–11] published on the JSUM website.
  6. Recording and scanning procedures
    There are a variety of image recording/storage methods depending on the institution, such as storage using a thermal printer, storage as DICOM data, and video storage on videotape/DVD. However, storage of DICOM data on electronic media is recommended from the viewpoint of secondary image interpretation and sending referral attachments to facilities for thorough examination.
    Perform measurements with the image adequately enlarged on the monitor, and denote measurements in millimeters, rounding off to the nearest integer. (However, evaluation of an entire organ can be denoted in centimeters.)
    For Category 3 or severer lesions and focal lesions, always record images from multiple directions, and also record and save the maximum diameter/scan site at the same time.
    With respect to the scanning procedure, carefully and thoroughly observing the entirety of each organ as a basic ultrasound scan, and properly evaluating not only focal lesions but also diffuse lesions throughout an entire organ, are fundamental.
    With respect to standards for sections to store during examinations, there is no nationwide uniform method. However, fundamental sections should be established to properly deal with situations, such as demonstration of the examination region, quality control, double interpretation, comparison of changes over time, referral to other institutions, education, and transfer of examiners/examinees.
    With respect to the position of the examinee, scans are almost always performed with the examinee in the supine position, but since the visualization capability of ultrasonography changes with a change of position, appropriate position changes (e.g., right and left half side-lying position, right and left lateral position, semi-sitting position, and sitting position) should be utilized in cases where observation in the supine position is insufficient.
    The time required for examinations will differ depending on the examination environment, but about 5-6 people without findings can be screened per hour (Ample time should be allocated as examination duration correlates with examination accuracy and the ability of the examiner.)
    Twenty-five recommended sections to record [12] and images by position change are presented here (Fig. 1, Fig. 2).
  7. Examination results/image interpretation/ultrasound image findings/assessments/post-examination management
    For examination results, record the results using the categories described later together with a record of the ultrasonography findings (Table 1-1).
    With respect to examinations performed by a technician, a JSGCS gastrointestinal cancer screening general certified physician or gastrointestinal cancer screening certified physician (hepatobiliary system and pancreatic), JSUM specialist, Board Certified Physician of JSND, Health Evaluation and Promotion Specialist of JSHEP/JSND, JRS radiology specialist, or JSLM clinical laboratory test specialist should make the final interpretation/diagnosis and complete a report.

**Table 1:** Categories

Category 0	Unable to visualize	Assessment is impossible due to device malfunction or examiner/examinee factors, etc
Category 1	Normal	There are no abnormal findings
Category 2	Benign	A definite benign lesion is present. Including normal variations
Category 3	Difficult to assess malignancy	Lesions difficult to assess for benignancy/malignancy, or indirect findings indicating a possible malignant lesion are present including high-risk group
Category 4	Possibly malignant	A lesion likely to be malignant is present
Category 5	Malignant	A definite malignant lesion is present

(4) Examination interval
  Recommend annual screening even if there are no abnormal findings.
(5) Selection of institution for thorough examination
  Referral to an institution for thorough examination is an important factor for examinees; therefore, examinees should be instructed to visit and referred to an appropriate medical institution according to the screening results. In addition, it is important to maintain a line of communication with the receiving institution, so that requests can be made for feedback on the results of thorough examinations as it will serve as important information at annual screenings.
  When writing a referral, include not only the test results but also clearly state the details of the request for thorough examination, and also attach images from the entire examination (DICOM digital images are recommended).
(6) Quality control
  Controlling the screening process as a whole so that it is always properly performed is important in order for ultrasound screening to continue to be effective as a cancer screening modality going forward. In addition to internal quality control, it is recommended that the process also undergoes periodic outside evaluation.
  In addition to control of the examination environment including the diagnostic unit, quality control includes but is not limited to tabulation and management of examination results (e.g., percentage of examinees who required thorough examination, percentage of examinees who underwent thorough examination by category assessment, and cancer detection rate), ascertainment and tabulation of post-examination guidance (e.g., ascertainment and tracking of examinees who did and did not undergo thorough examination, screening recommendation, and ascertainment of screening sensitivity/specificity), and cooperation with and registration in nationwide tabulation.
(7) Education
  Continued education for physicians, clinical technologists, radiology technicians, nurses, and others involved in screening is important to update knowledge and improve skills.
  In addition to holding in-house review meetings to iron out differences of opinion and help improve diagnostic accuracy, an institution should create a system that allows team members to periodically participate in conferences, workshops, and training courses. Furthermore, providing support and cooperation aimed at team members acquiring JSGCS, JSUM, JSND, JSLM, and other qualifications is also important.
(8) Recommended images to record (Figs. 1, 2).

## Categories and assessments

**Fig. 3:**
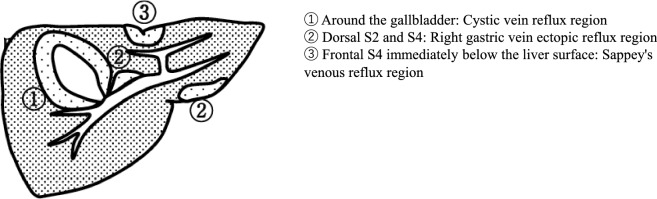
Commonly focal spared area in fatty liver. If it is an irregular hypoechoic area in a commonly focal spared area without a disturbed speckle pattern, and no deviation in blood flow is detected on color Doppler, it is not considered a solid lesion (Figs. Liver-6 to 8)

1. Ultrasound imaging findings
  Operators should consider in detail to which ultrasound imaging finding item in the Manual the abnormal findings noted in observations of the liver, biliary tract, pancreas, spleen, kidneys, abdominal aorta, and other target organs correspond and select the applicable item.
  Although observation of organs other than the target organs is not essential, describe any findings that are noted.
  If an organ cannot be visualized at all, it should be assessed as "Unable to visualize." If part of an organ cannot be visualized, it should be assessed as "Poorly visualized" and regarded as equivalent to "Difficult to visualize" and "Inadequately visualized," and cases where the border is indistinct after partial resection, etc., should be included in this category; clearly describe poorly visualized sites and use findings from sites that could be visualized.

(2) (Categories (Tables 1, 2)
  The cancer-related category, ultrasound findings (described in Report Form), and assessment are determined in accordance with the ultrasound imaging findings selected.
  Categories are criteria for cancer detection and also summaries of findings noted during ultrasonography.
  For each organ, the highest category should be recorded as the category for the organ. However, in cases where the highest category and highest assessment differ, both should be recorded (e.g., Category 2/Assessment D2, Category 3/Assessment C).
  For a lesion that can be compared with a past image, record comments on any chronological changes.
  If a lesion has findings corresponding to Assessment D2 or higher on ultrasound images but has been deemed to be benign as a result of thorough examination, the relevant category is indicated with an apostrophe [e.g., 0', 2', 3', 4'], and the assessment is C. (Note that the high-risk group is not included in this category.) (Fig. 3)

**Table 2:** Category table (example)

Organ	Category assessment	Poorly visualized site
Liver	0 / 1 / 2 / 3 / 4 / 5	Yes□
Biliary tract		
Gallbladder	0 / 1 / 2 / 3 / 4 / 5	Yes□
Extrahepatic bile duct	0 / 1 / 2 / 3 / 4 / 5	Yes□
Pancreas	0 / 1 / 2 / 3 / 4 / 5	Yes□
Spleen	0 / 1 / 2 / 3 / 4 / 5	Yes□
Kidneys	0 / 1 / 2 / 3 / 4 / 5	Yes□
Abdominal aorta	0 / 1 / 2 / 3 / 4 / 5	–
Other		–

Shaded cells are filled in only if there are applicable findings

(3) Ultrasound findings (described in Report Form)
  It consists of simplified terms for notification of ultrasound imaging findings to examinees. Ultrasound findings are described in the Report Form. Categories 4 and 5 are described as “Tumor” and Category 3 focal lesions as “Mass,” including suspected ones.
(4) Assessment (Table 3)

**Table 3:** Assessment

A	Normal
B	Mild abnormality
C	Reexamination (3/6/12 months)/lifestyle improvement needed
D (Medical care needed)	
D1	Treatment needed
D1P	Treatment needed (urgent)
D2	Thorough examination needed
D2P	Thorough examination needed (urgent)
E	Under treatment

Cases deemed to be urgent, such as abdominal aortic aneurysm with high likelihood of rupture and aortic dissection, should be assessed as D1P or D2P (P: panic finding)

As a rule, the final assessment is determined by the physician in charge of assessment in accordance with the Manual based on abnormal findings on ultrasound images. However, the physician in charge of assessment can change the assessment as necessary based on test results other than ultrasonography and comparison with previous findings.

Notes

- P: Panic finding, Promptly report Category 5 lesions to the physician in charge of assessment.
- Promptly report it to the physician in charge of assessment in the case of findings indicative of transition to an emergency situation such as biliary stone.
- For Assessment C, the term "Follow-up observation needed" had been used until now, but the timing of follow-up observation was unclear; therefore, "Reexamination needed" will be used instead going forward to standardize the terminology at institutions, and the specific timing will be entered.
- The reexamination timing selected was 3/6/12 months, but it can be changed at the direction of the physician in charge of assessment.
- Reexaminations will be performed at a medical institution as necessary, but in the case of the 12-month reexamination, the examinee should be strongly recommended to undergo screening the following year.
- In the case of Category 2', 3', or 4' with Assessment C, ultrasonography at the annual screening 12 months later may be used as a substitute for reexamination.
- If "Reexamination needed" is selected, the examinee should be given specific instructions regarding the institution to visit for reexamination.
- If "Thorough examination needed" is selected, the examinee should be given specific instructions regarding the institution to visit for thorough examination and examination procedures, etc.
- When the examinee undergoes reexamination (e.g., during dietary therapy for hepatic steatosis, or main pancreatic duct dilatation/pancreatic cyst) either in-house or at another institution (every 3/6/12 months), the assessment should be C, not E.
- Assessment C or E may be selected if the examinee has undergone thorough examination at another medical institution and continues to be followed up by the institution. However, for an examinee in the group at high risk for cancer, Assessment D2 may be selected after hearing the details of examinations performed at the medical institution.
- A Category 3 lesion may be assessed as C if there are no chronological changes compared with at least the past 2 results.
- Assessment D2 may be selected as necessary if the diameter of the focal lesion or lumen definitely increases compared to the previous result.
- If organ atrophy is found, refer to the past medical history, history of present illness, or treatment history when making the assessment.
- In the case of total resection, partial resection, or detection of treatment scar, refer to the past medical history or history of present illness when making the assessment.
- Make use of color Doppler ultrasound as appropriate to aid assessment.
- Assessment D2 may be selected as necessary for a focal lesion in the liver if chronic hepatic disease is suspected based on clinical biochemistry data such as infection with HBV or HCV or presence of thrombocytopenia (<15,000/μL).
- Assessment D2 may be selected if a biliary tract enzyme abnormality is found in a case with a poorly visualized extrahepatic bile duct.

(5) Category and assessment table for each organ (Tables 4, 5, 6, 7, 8, 9, 10)

**Table 4:** Liver

Liver				
Ultrasound imaging finding	Category	Ultrasound finding (described in Report Form)	Assessment	Figure number
Post-resection^*1)^ Post-transplantation	2	Post-partial liver resection Post-liver transplantation	B	
Post-local treatment	3	Post-local liver treatment	C	Figure 4
Congenital deformity^*2)^	2	Liver deformity	B	
Unable to visualize	0	Unable to visualize liver	D2	
Diffuse lesion				
Any one of bright liver, liver-kidney (spleen) contrast, deep attenuation, or intrahepatic vascular blurring is present^*3)^	2	Fatty liver	C	Figures 5, 6, 7, 8
Dull hepatic edge /rough parenchymal echo pattern or nodular rugged liver surface is present (either one)^*4)^	3	Chronic liver disease suspected	C	Figures 9, 10
Dull hepatic edge/rough parenchymal echo pattern and nodular rugged liver surface are present (all)^*4)^	3	Chronic liver disease	D2	Figures 11, 12, 13
Solid lesion				
A solid lesion is present	3	Liver mass	C	Figure 14
Solid lesion with involvement of Category 3/Assessment D2 diffuse lesion	4	Liver tumor suspected	D2	Figure 15
Maximum diameter ≥ 15 mm	4	Liver tumor suspected	D2	Figure 16
Hepatic neoplastic lesion				
Any one of marginal strong echo, chameleon sign, wax and wane sign, or disappearing sign is present^*5)^	2	Liver hemangioma	C	Figures 17, 18, 19, 20
Any one of peripheral hypoechoic zone, posterior echo enhancement, or multiple lesions is present	4	Liver tumor suspected	D2	Figures 21, 22
Peripheral bile duct dilatation	4	Liver tumor suspected	D2	Figure 23
Any one of mosaic pattern, bright loop appearance, or hump sign^*6)^ is present	5	Liver tumor	D1	Figures 24, 25, 26
Either cluster sign or bull's eye pattern^*7)^ is present	5	Liver tumor	D1	Figures 27, 28
Disruption /tumor embolism of either intrahepatic bile duct or blood vessel is present	5	Liver tumor	D1	Figure 29
Cystic lesion				
Cystic lesion (without the following findings regardless of the size)	2	Liver cyst	B	
Solid component (mural nodules/wall thickening/septal thickening) and change in internal fluid (e.g., internal echogenic spots) are present^*8)^	4	Hepatic cystic tumor suspected	D2	Figures 30, 31
Peripheral duct dilatation^*9)^	3	Liver cyst with intrahepatic bile duct dilatation	D2	Figure 32
Other findings				
Calcified lesion^*10)^	2	Intrahepatic calcification/intrahepatic stone	C	Figure 33
Pneumobilia	2	Pneumobilia	B	Figure 34
Intrahepatic bile duct dilatation maximum diameter ≥ 4 mm (≥ 6 mm after cholecystectomy) ^*11)^	3	Intrahepatic bile duct dilatation	D2	
Without abnormal findings in the bile duct up to the near-papillary region	2	Bile duct dilatation	C	
Vascular abnormality^*12)^	2	Liver vascular abnormality	D2	Figure 35
No abnormal findings	1	Normal liver	A	

^*1)^ Those without findings of recurrence after local treatment are not regarded as neoplastic lesions. In the case of partial resection, document the resection site if known, and evaluate the ultrasound imaging findings of the remaining portion

^*2)^ A congenital deformity (e.g., partial atrophy) is regarded as Category 2 and Assessment B, and parts other than the deformed part are evaluated using the same method as others

^*3)^ Compare the brightness of the liver parenchyma with the healthy kidney at the same depth (compare with the spleen in cases with chronic renal failure). Check the spleen-kidney contrast and evaluate the liver–kidney contrast if there is no difference in brightness between the spleen and kidney. If it is an irregular hypoechoic area in a commonly focal spared area without a disturbed speckle pattern, and no deviation of blood vessel is detected on color Doppler, it is not considered a solid lesion (Fig. 3)

^*4)^ During evaluation of the liver parenchyma, if the flag sign or bamboo blind sign is found, include it in rough parenchymal echo pattern

^*5)^ Lesions in which an internal change is detected such as the "sludge worm" sign are placed in this category

^*6)^ Mosaic pattern (same as nodule in nodule): An echo pattern formed by a mosaic array of nodules within a mass. Characteristically seen in hepatocellular carcinoma. Bright loop appearance: This term refers to the state after dedifferentiation of hepatocellular carcinoma in which hypoechoic nodules appear in hyperechoic nodules. Hump sign: This refers to observation of a protrusion on the surface of a solid organ made by a tumor, etc.

^*7)^ Cluster sign: Visualized as a cluster of many aggregated masses, it is characteristic of metastatic liver tumors. Bull's eye pattern (same as target sign): An echo pattern where the internal echo of a mass, etc., exhibits a concentric circular structure

^*8)^ All cystic lesions with definite wall thickness should be regarded as wall thickening. Changes in internal fluid (e.g., intracystic hemorrhage/infection) are also subject to thorough examination as the possibility of neoplasticity cannot be ruled out. In addition, neoplastic cysts, the generic term for cysts covered with cells showing neoplastic proliferation, are also included in this category

^*9)^ If peripheral bile duct dilatation due to a hepatic cyst is present, thorough examination is required as there is a possibility of cystic tumor involvement and cases in which treatment is indicated are included

^*10)^ The comet-like echo found in biliary hamartoma, etc., is included. Pneumobilia and calcification/lithiasis should be differentiated by movement status at position change or while breathing

^*11)^ The threshold for dilatation of the intrahepatic bile duct is ≥ 4 mm (round off to the nearest integer) (right and left hepatic ducts are extrahepatic hepatic ducts). Localized bile duct dilatation without a mass lesion and post-bile duct surgery are also included

^*12)^ In addition to portal-venous shunts, arterial-portal shunts, and arterial-venous shunts, vascular abnormalities include but are not limited to portal hypertension findings including extrahepatic collateral flow, aneurysms, and portal aneurysms. However, mild portal aneurysms or portal-venous shunts deemed to have no effect on the pathologic condition should be regarded as Category 2 and Assessment C. In addition, mass lesion-related vascular abnormalities should be evaluated in the same manner as mass lesions (Fig. 36)

**Fig. 4:**
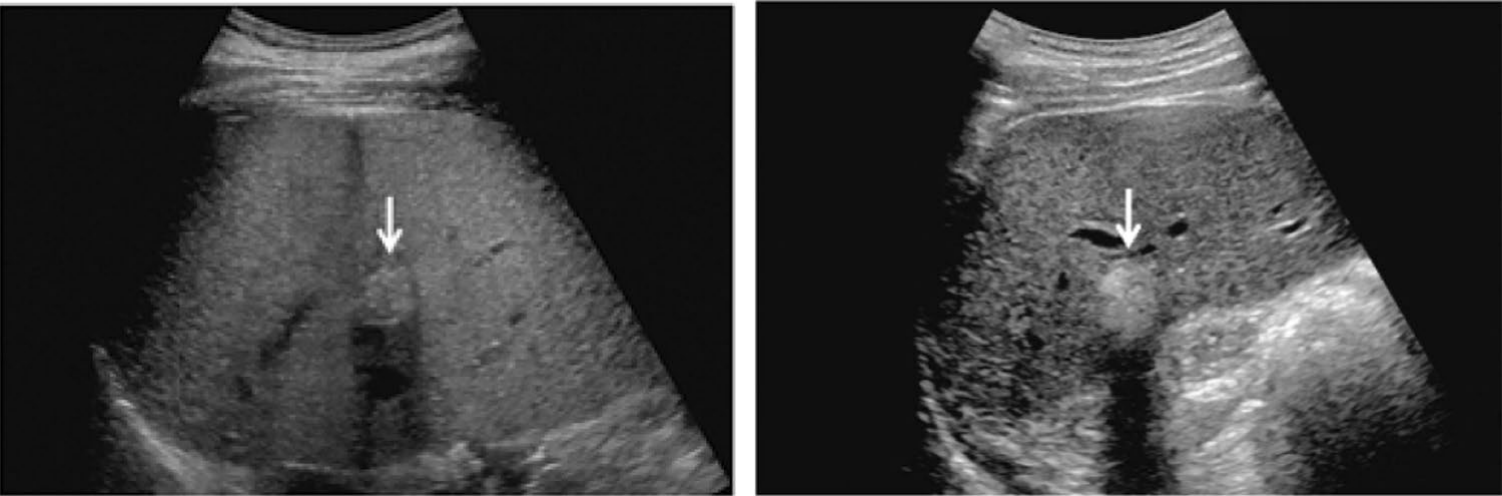
Post-local treatment. Left: post-radiofrequency ablation; right: post-hepatic arterial embolization. Category 3, Assessment C  Please align the size of images(8,13-16, 21-26, 28-30, 32-34)

**Fig. 5:**
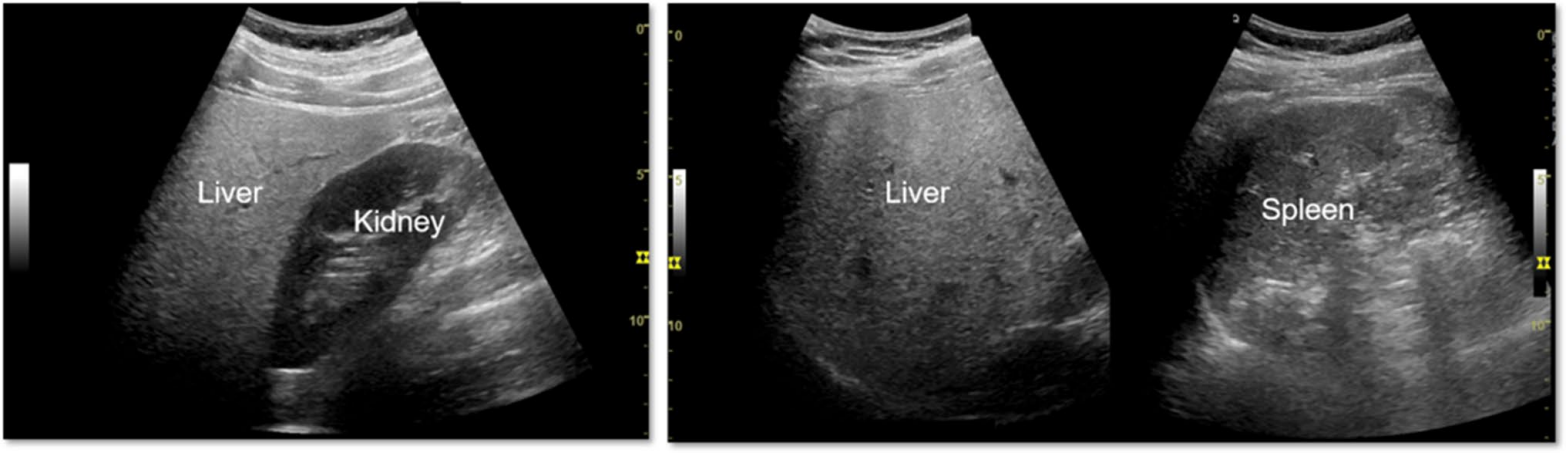
Diffuse lesion. Any one of bright liver, liver-kidney (spleen) contrast, deep attenuation, or intrahepatic vascular blurring is present. Left: liver–kidney contrast; right: liver–spleen contrast. Category 2, Assessment C

**Fig. 6:**
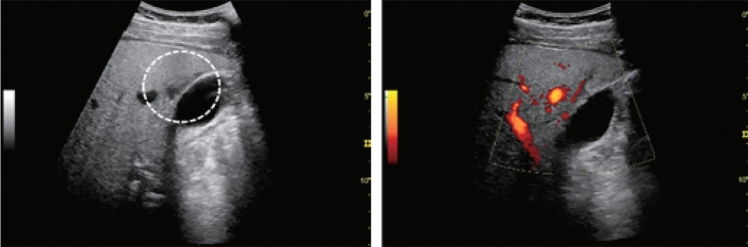
Diffuse lesion. Commonly focal spared area in fatty liver (Around the gallbladder: Cystic vein reflux region). Left: B-mode, Right: Power Doppler. Category 2, Assessment C

**Fig. 7:**
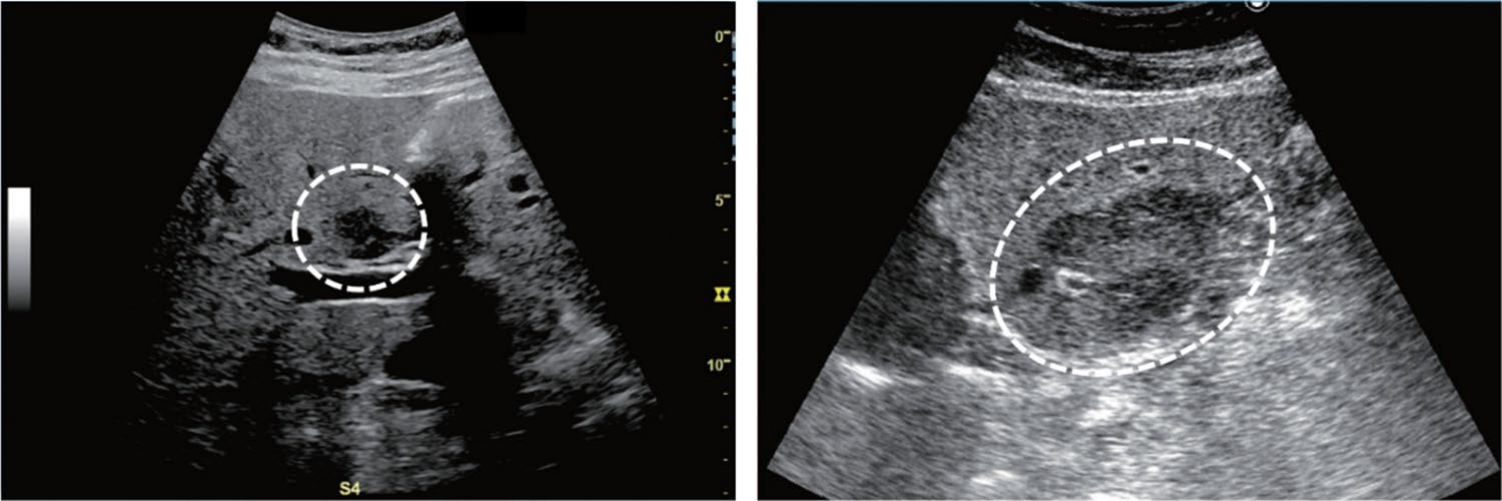
Diffuse lesion. Commonly focal spared area in fatty liver (Right gastric vein ectopic reflux region). Left: Dorsal S4, Right: Dorsal S2. Category 2, Assessment C

**Fig. 8:**
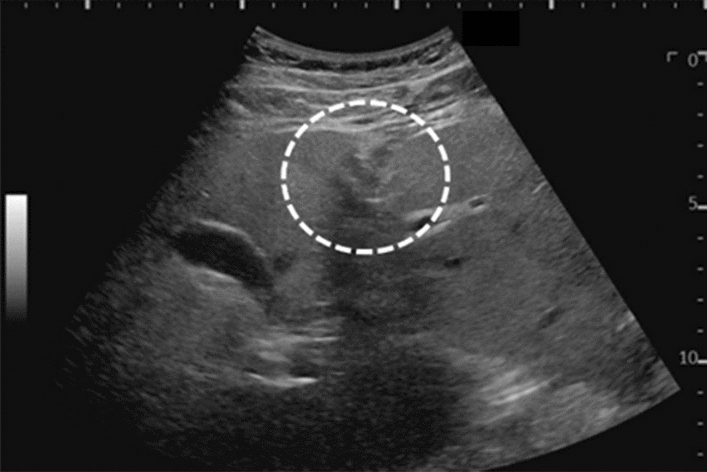
Diffuse lesion. Commonly focal spared area in fatty liver (Frontal S4 immediately below the liver surface: Sappey's venous reflux region). Category 2, Assessment C

**Fig. 9:**
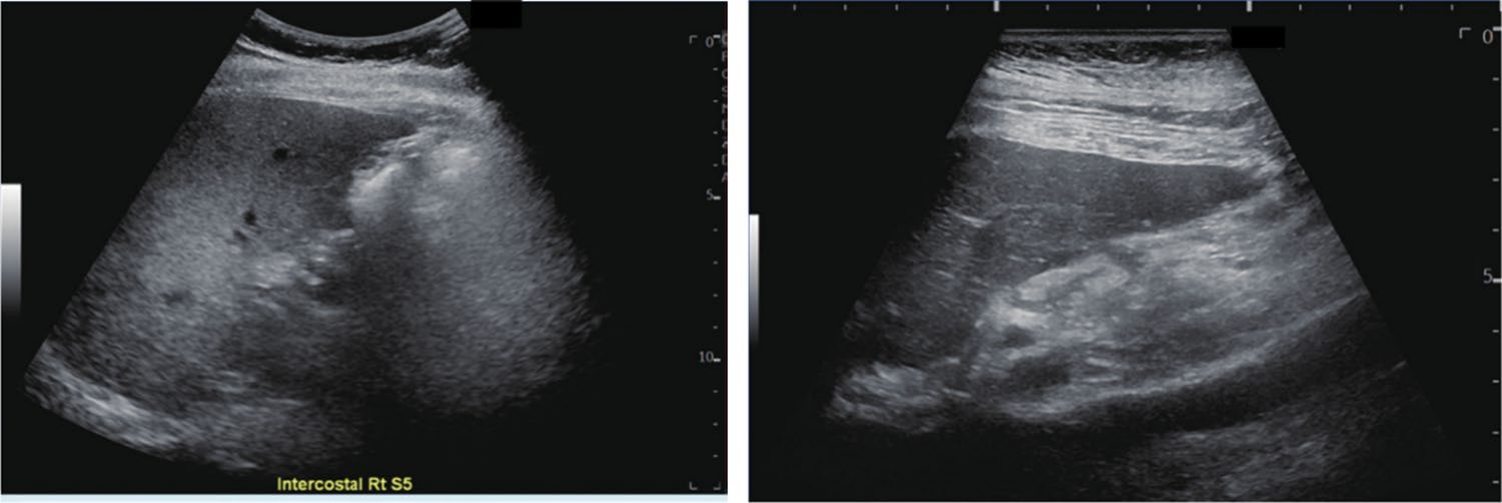
Diffuse lesion. Dull hepatic edge/rough parenchymal echo pattern or nodular rugged liver surface is present (either one) (only dull hepatic margin is noted). Left: right intercostal scan (right hepatic lobe) with convex probe; right: midline vertical scan (left hepatic lobe) with high-frequency probe. Category 3, Assessment C

**Fig. 10:**
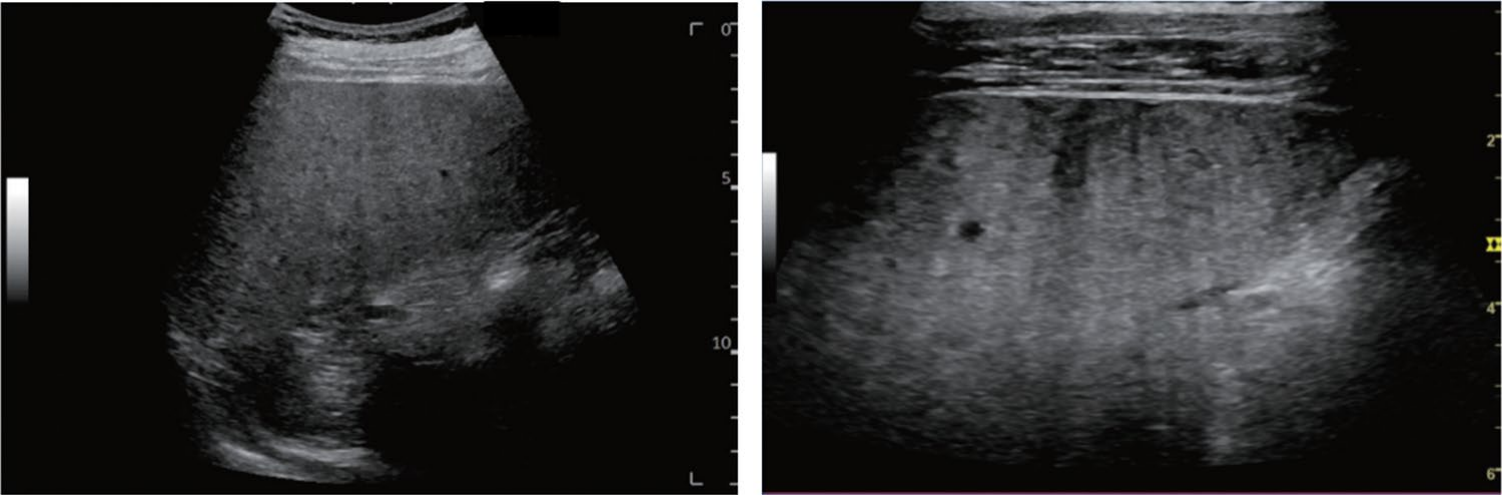
Diffuse lesion. Dull hepatic edge/rough parenchymal echo pattern or nodular rugged liver surface is present (either one). Bamboo blind sign (include in rough echo pattern). Left: right intercostal scan with convex probe; right: right intercostal scan with high-frequency probe. Category 3, Assessment C

**Fig. 11:**
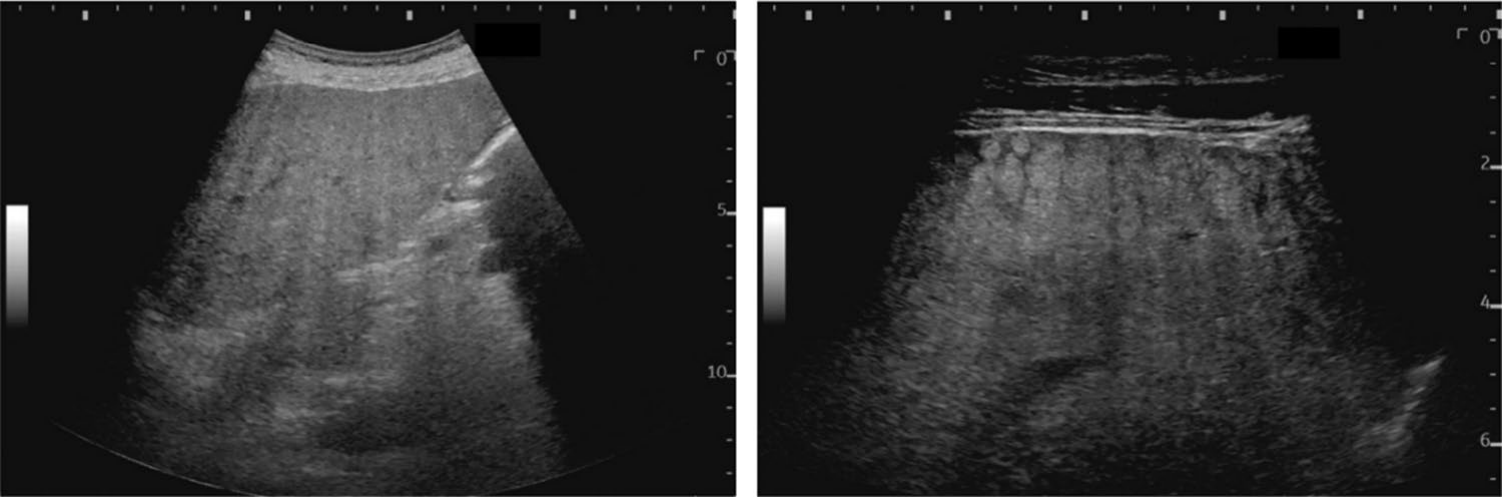
Diffuse lesion. Dull hepatic edge/rough parenchymal echo pattern and nodular rugged liver surface are present (all) (flag sign). Left: midline vertical scan with convex probe, right: midline vertical scan with high-frequency probe. Category 3, Assessment D2

**Fig. 12:**
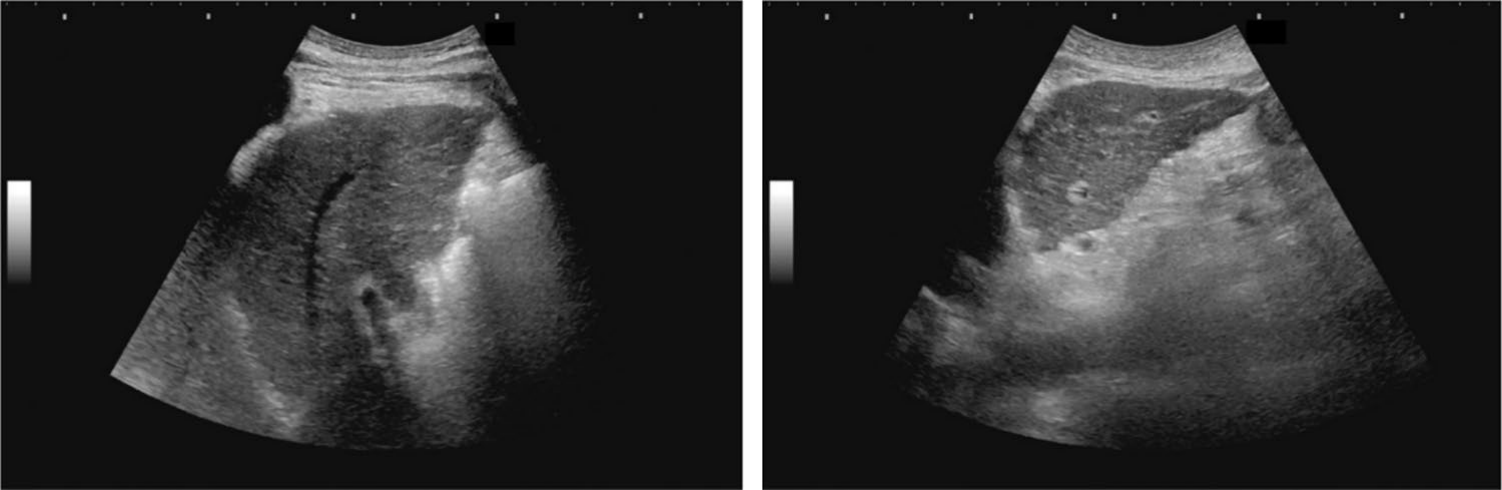
Diffuse lesion. Dull hepatic edge/rough parenchymal echo pattern and nodular rugged liver surface are present (all). Left: right intercostal scan (right hepatic lobe), right: midline vertical scan (left hepatic lobe). Category 3, Assessment D2

**Fig. 13:**
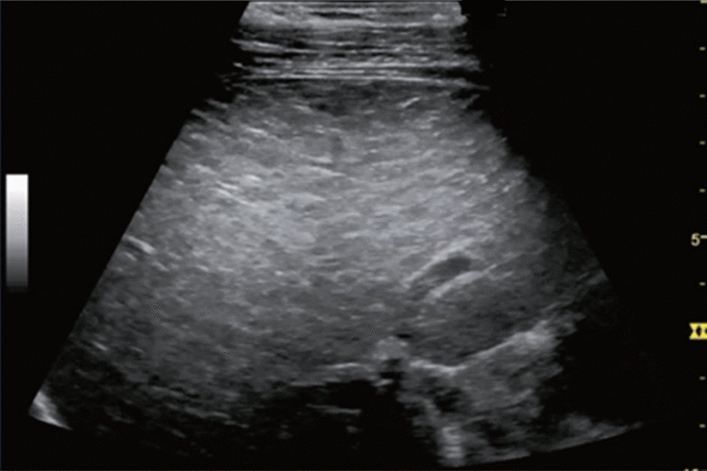
Diffuse lesion. Dull hepatic edge/rough parenchymal echo pattern and nodular rugged liver surface are present (all) (high-frequency probe). Category 3, Assessment D2

**Fig. 14:**
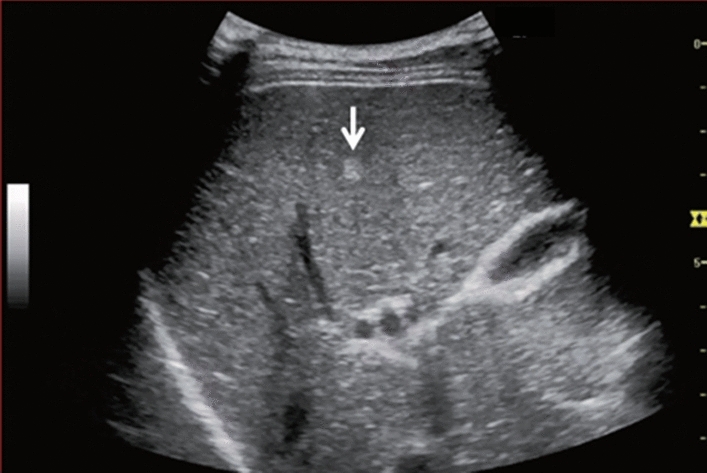
Solid lesion. A solid hepatic lesion is present (maximum diameter < 15 mm). Category 3, Assessment C

**Fig. 15:**
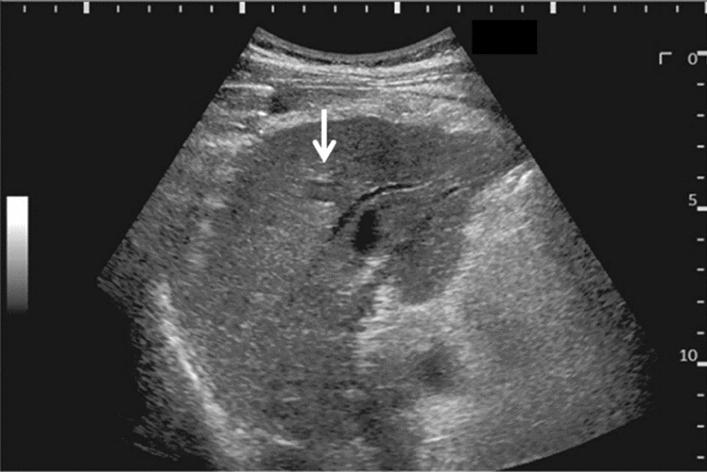
Solid lesion. Solid lesion with involvement of Category 3 diffuse lesion. Category 4, Assessment D2

**Fig. 16:**
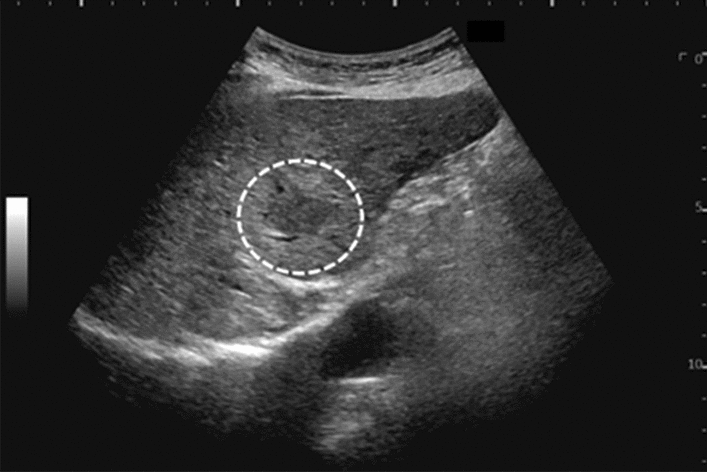
Solid lesion. A solid lesion is present. Maximum diameter ≥ 15 mm. Category 4, Assessment D2

**Fig. 17:**
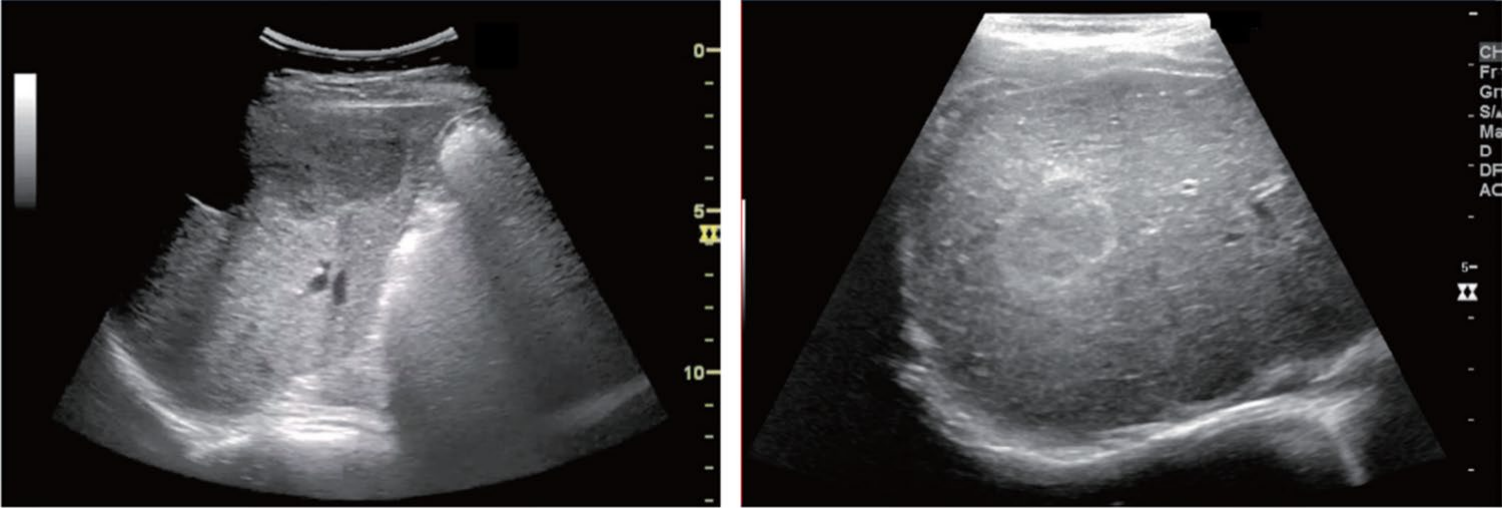
Hepatic neoplastic lesion. Marginal strong echo. Left: Convex probe, Right: High-frequency probe. Category 2, Assessment C

**Fig. 18:**
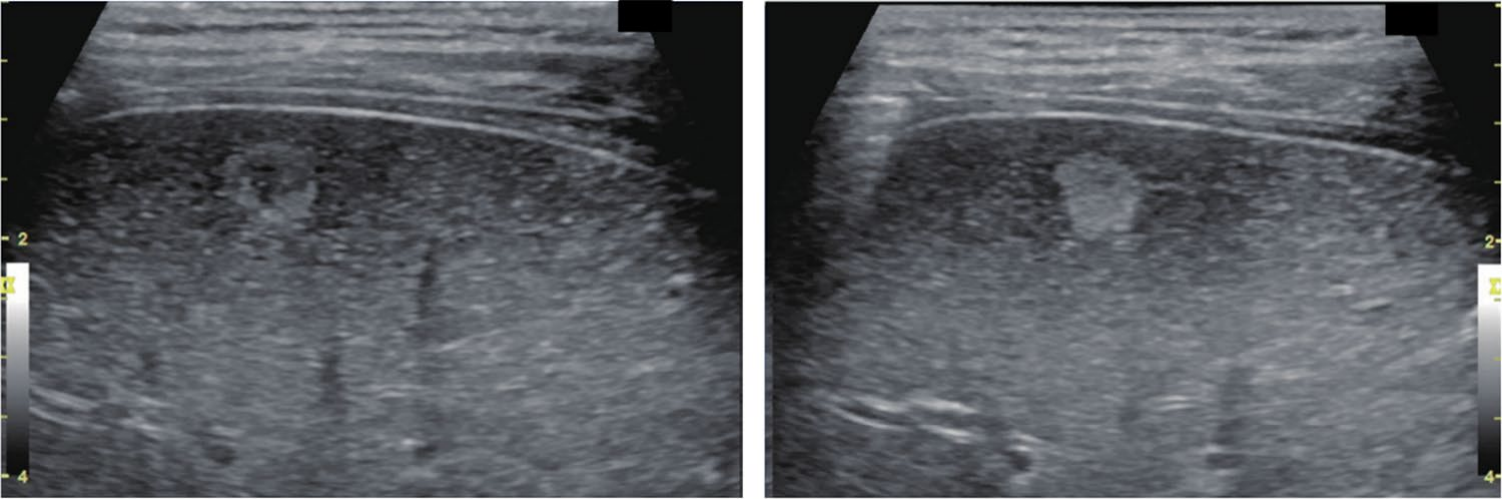
Hepatic neoplastic lesion. Chameleon sign/wax and wane sign (change in internal echo detected). Category 2, Assessment C

**Fig. 19:**
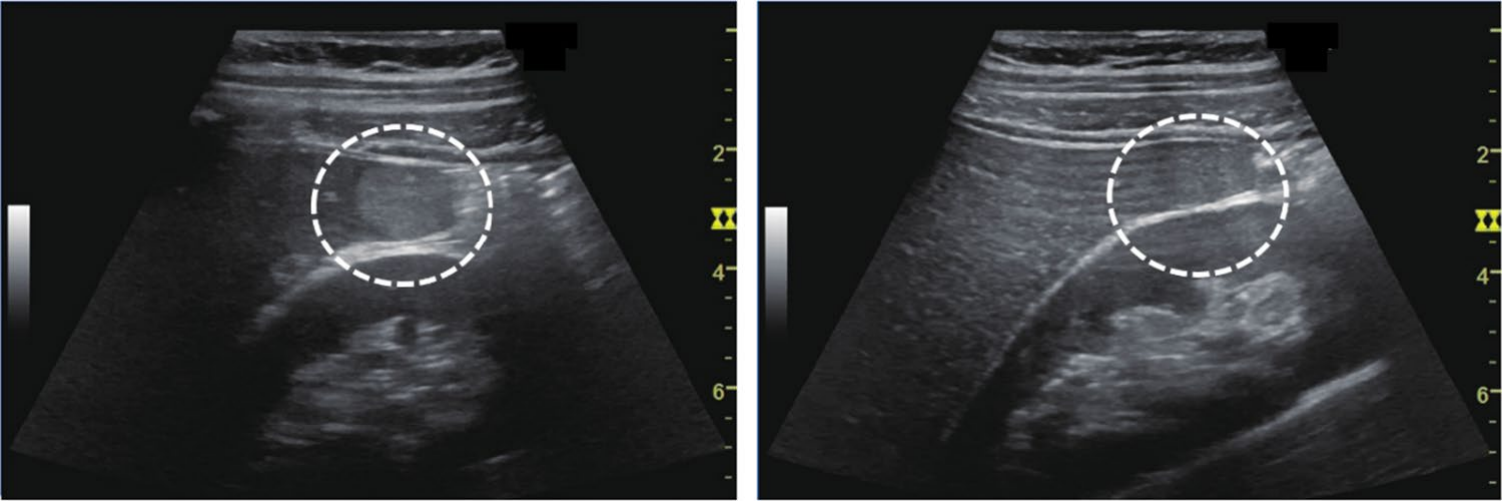
Hepatic neoplastic lesion. Disappearing sign. Left: Before compression, Right: After compression. Category 2, Assessment C

**Fig. 20:**
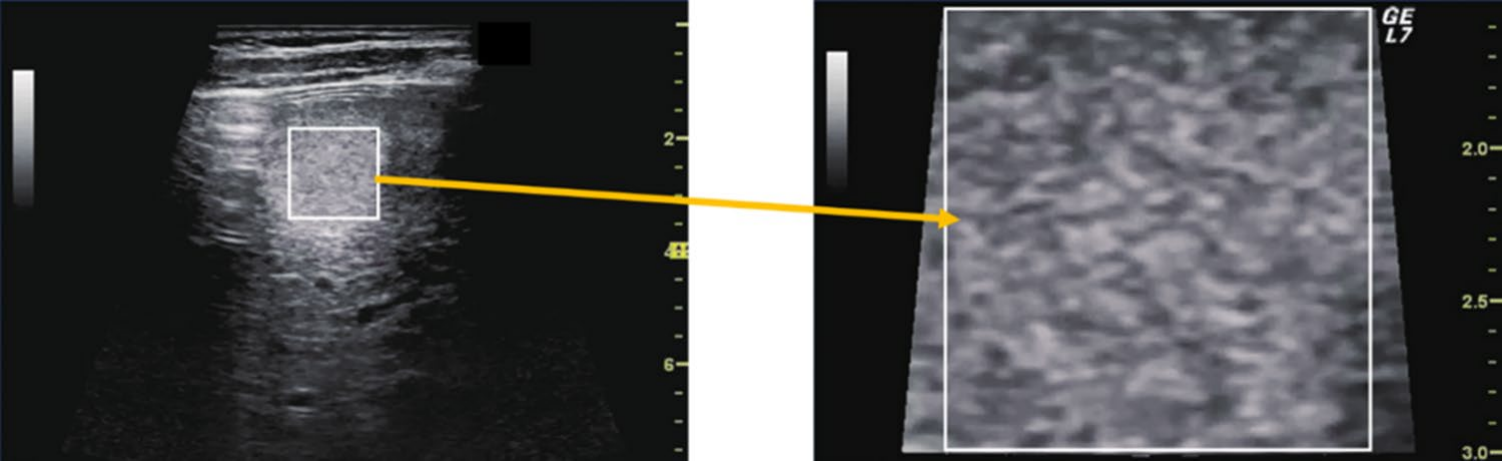
Hepatic neoplastic lesion. Sludge worm sign. Left: High-frequency probe, Right: Zoom image. Category 2, Assessment C

**Fig. 21:**
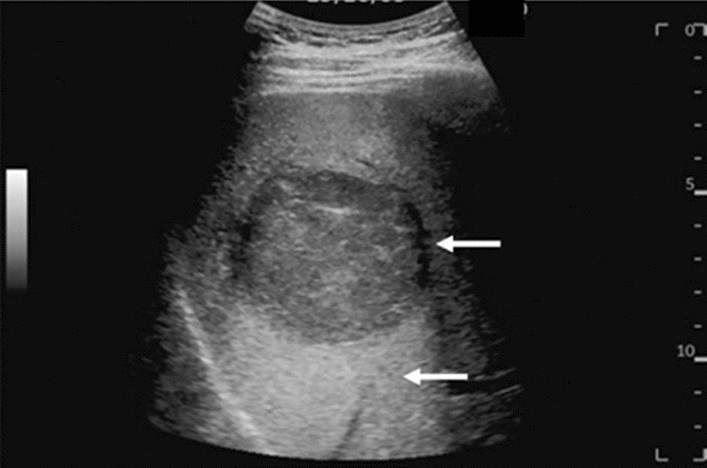
Hepatic neoplastic lesion. Peripheral hypoechoic zone/posterior echo enhancement. Category 4, Assessment D2

**Fig. 22:**
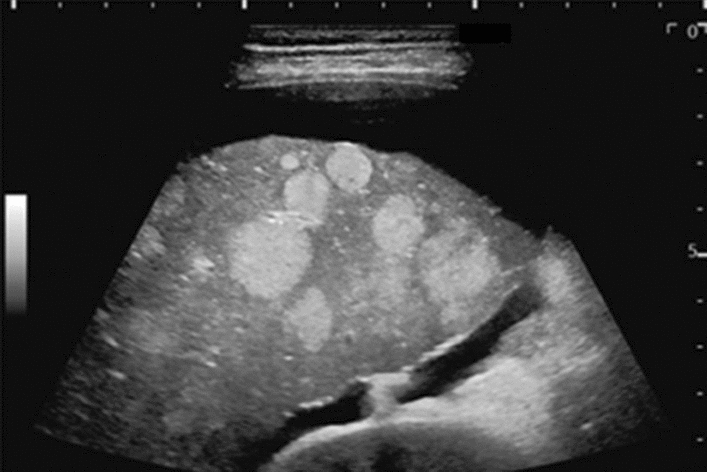
Hepatic neoplastic lesion. Multiple neoplastic lesions. Category 4, Assessment D2

**Fig. 23:**
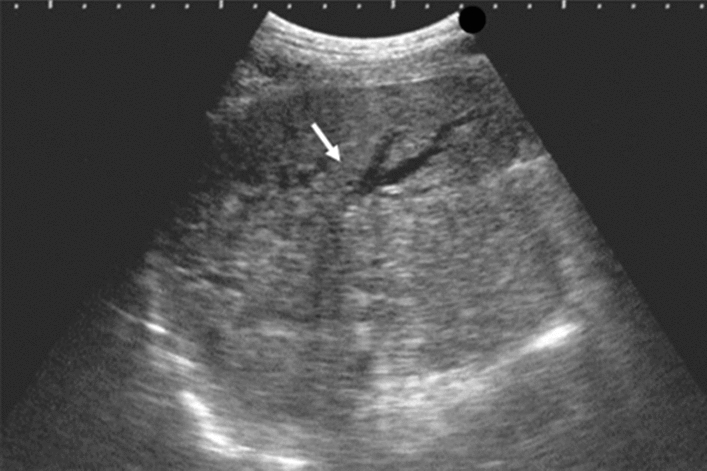
Hepatic neoplastic lesion. Distal bile duct dilatation. Category 4, Assessment D2

**Fig. 24:**
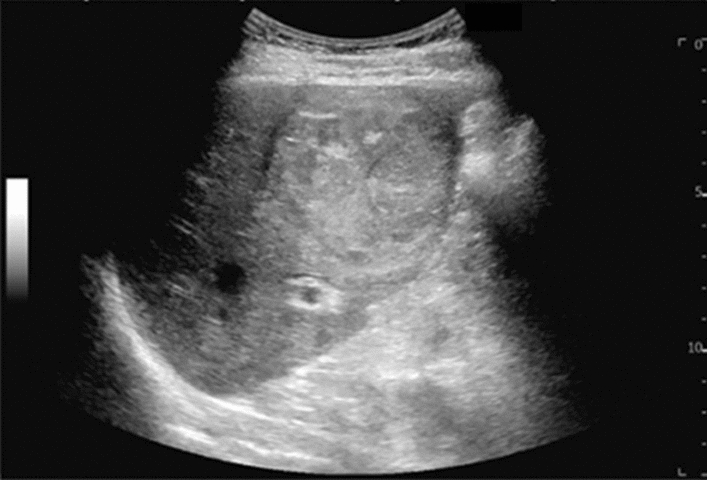
Hepatic neoplastic lesion. Mosaic pattern. Category 5, Assessment D1

**Fig. 25:**
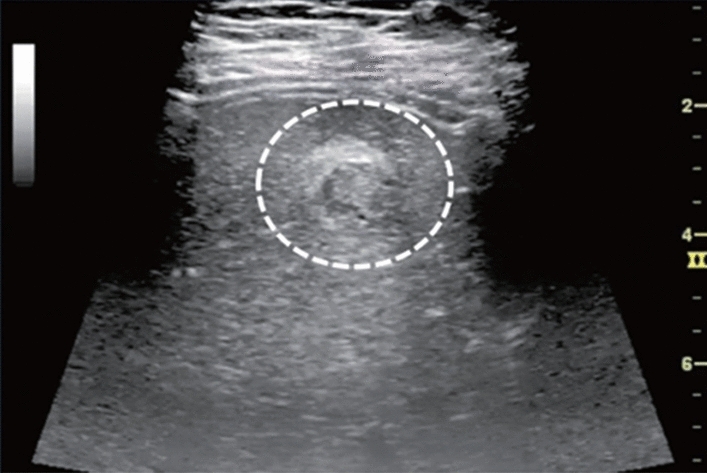
Hepatic neoplastic lesion. Bright loop appearance. Category 5, Assessment D1

**Fig. 26:**
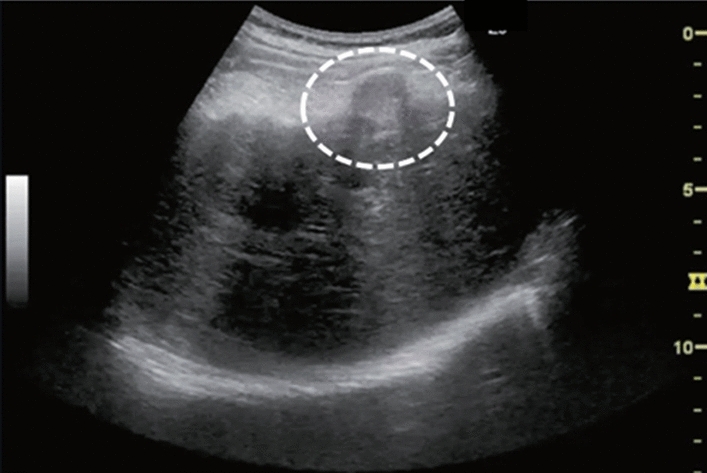
Hepatic neoplastic lesion. Hump sign. Category 5, Assessment D1

**Fig. 27:**
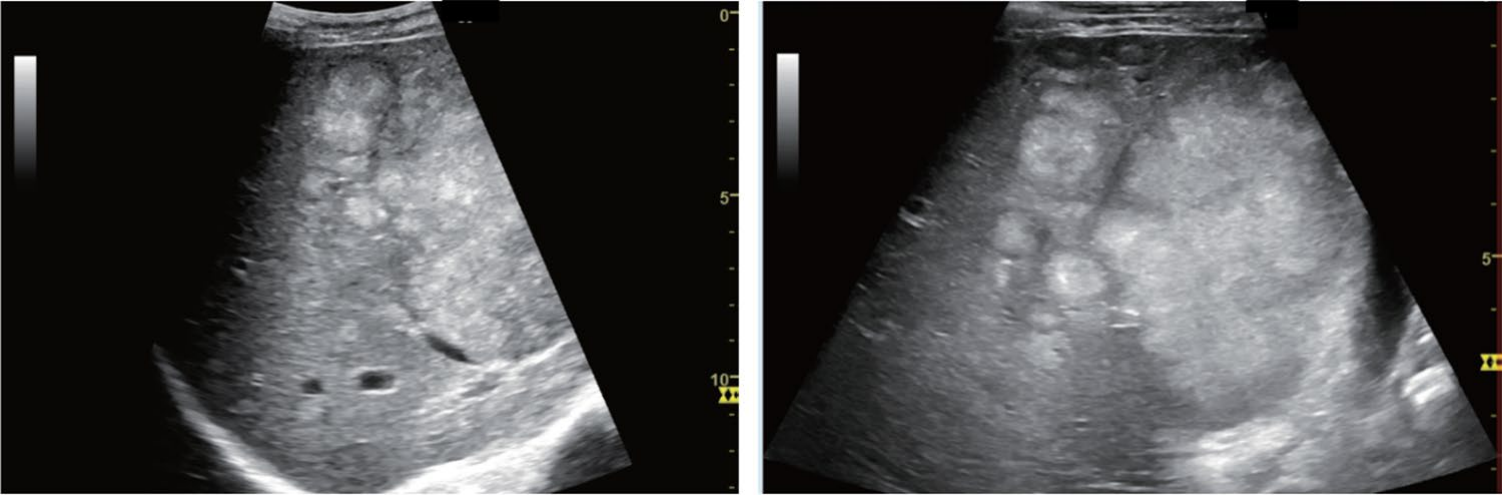
Hepatic neoplastic lesion. Cluster sign. Left: Convex probe, Right: High-frequency probe. Category 5, Assessment D1

**Fig. 28:**
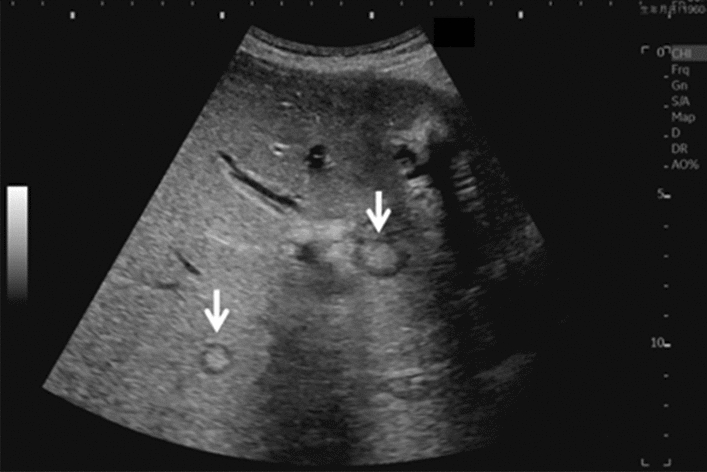
Hepatic neoplastic lesion. Bull's eye pattern (target sign). Category 5, Assessment D1

**Fig. 29:**
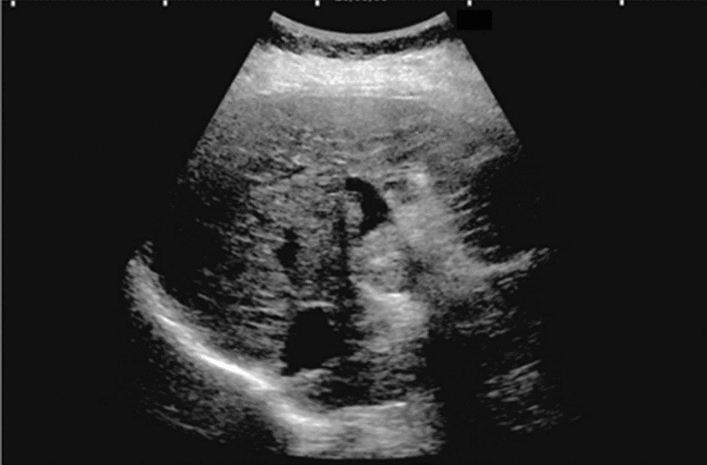
Hepatic neoplastic lesion. Intraportal tumor embolism is present. Category 5, Assessment D1

**Fig. 30:**
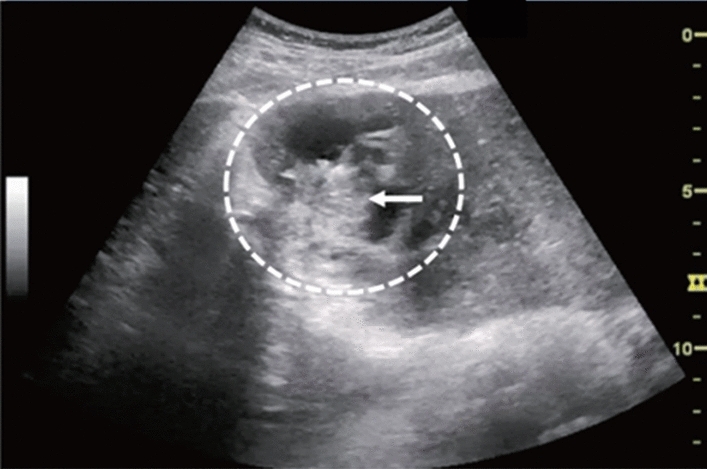
Cystic lesion. Solid component (intracystic nodules/wall thickening/septal thickening) is present. Intracystic nodules are present. Category 4, Assessment D2

**Fig. 31:**
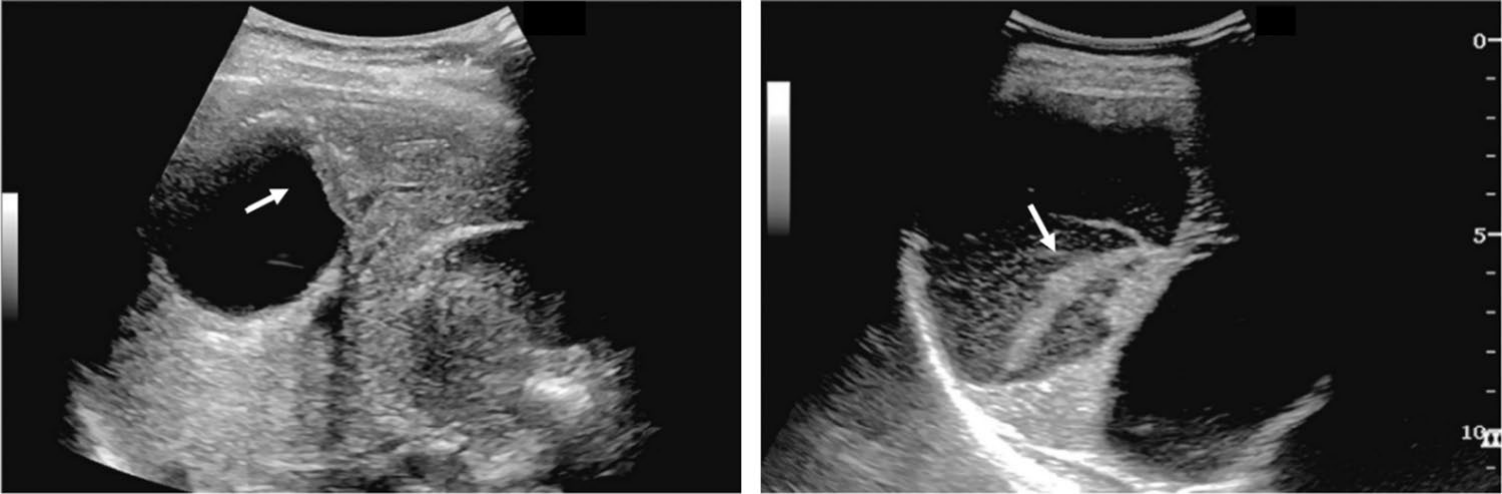
Cystic lesion. Solid component (intracystic nodules/wall thickening/septal thickening) and change in internal fluid (e.g., internal echogenic spots) are present. Left: cyst wall thickening; right: echogenic spots in internal fluid. Category 4, Assessment D2

**Fig. 32:**
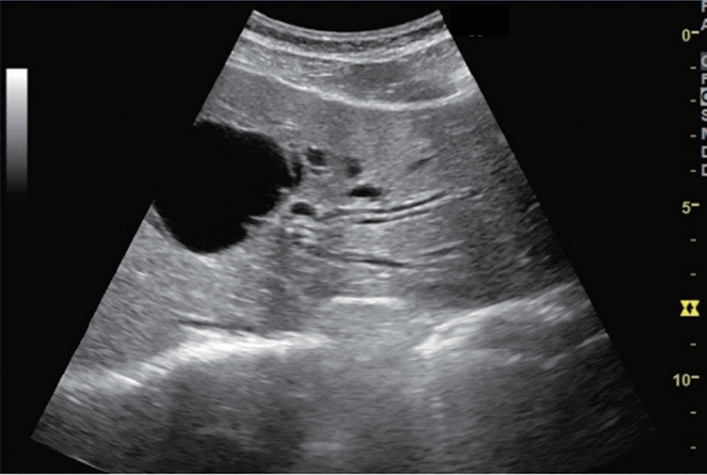
Cystic lesion. Distal bile duct dilatation. Category 3, Assessment D2

**Fig. 33:**
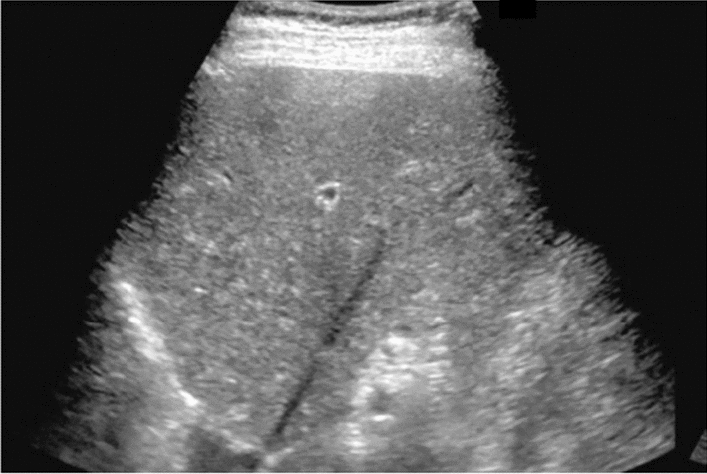
Other findings. Calcified opacity. Comet-like echo. Category 2, Assessment C

**Fig. 34:**
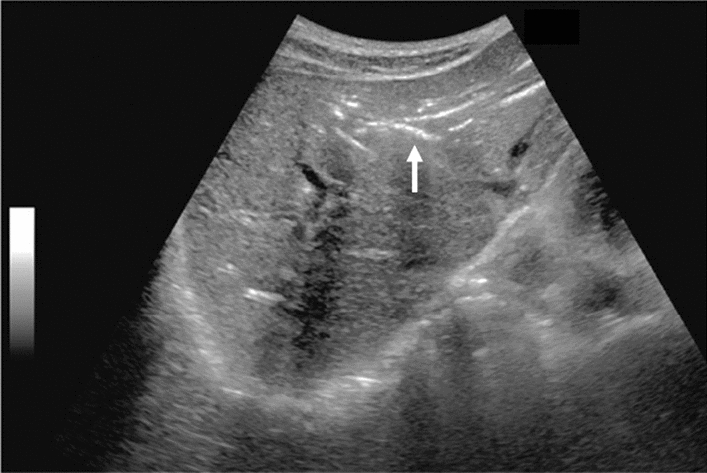
Other findings. Pneumobilia. Category 2, Assessment B

**Fig. 35:**
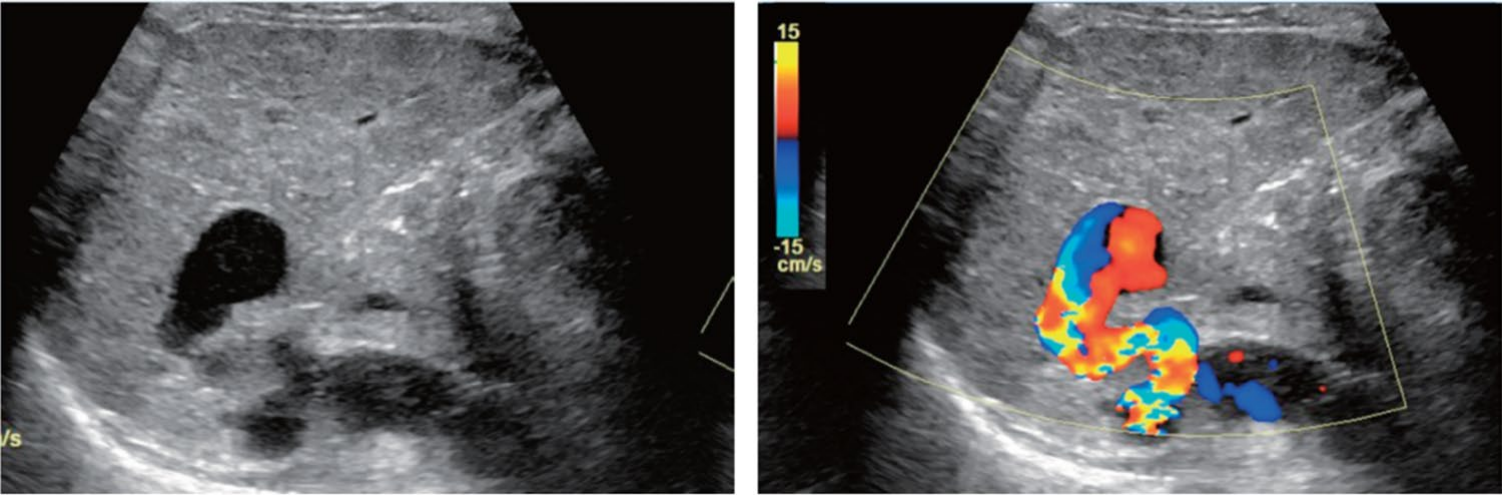
Other findings. Vascular abnormality (portal-venous shunt). Left: B-mode; right: color Doppler. Category 2, Assessment D2

**Table 5:** Gallbladder/extrahepatic bile duct

Gallbladder				
Ultrasound imaging finding	Category	Ultrasound finding (described in Report Form)	Assessment	Figure number
Biliary tract				
Post-resection^*1)^	0	Post-cholecystectomy	B	
Unable to visualize	0	Unable to visualize gallbladder	D2	
Wall inevaluable^*2)^	3	Gallbladder wall inevaluable	D2	Figures 37, 38
Morphological abnormality				
Maximum short diameter ≥ 36 mm^*3)^	3	Gallbladder distension	D2	Figure 39
Without abnormal findings in the bile duct up to the near-papillary region	2	Gallbladder distension	C	
Wall thickening^*4), 5)^				
Diffuse wall thickening (maximum wall thickness ≥ 4 mm at liver bed side of gallbladder body)	3	Diffuse gallbladder wall thickening	D2	Figure 40
If small cystic structure or comet-like echo is present	2	Gallbladder adenomyomatosis	C	Figure 41
With irregularity or disruption of the layered structure of the wall	4	Gallbladder tumor suspected	D 2	Figure 42
Localized wall thickening (internal hypoechoic layer is present in a part of the wall)	4	Gallbladder tumor suspected	D2	Figures 43, 44, 45
If small cystic structure or comet-like echo is present	2	Gallbladder adenomyomatosis	C	Figure 46
Protruded or mass lesion (polyp)				
Pedunculated				
Maximum diameter < 5 mm	2	Gallbladder polyp	B	Figure 47
Maximum diameter ≥ 5 mm, < 10 mm	3	Gallbladder mass lesion	C	
If mulberry-like echo or hyperechoic spot is present	2	Gallbladder polyp	B	Figures 48, 49
Maximum diameter ≥ 10 mm	4	Gallbladder tumor suspected	D2	Figure 50
Sessile (broad-based)	4	Gallbladder tumor suspected	D2	Figure 51
If small cystic structure or comet-like echo is present	2	Gallbladder adenomyomatosis	C	Figure 52
With irregularity or disruption of the layered structure of the attached wall	5	Gallbladder tumor	D1	Figure 53
Other findings				
Stone image (including calcified lesion) Pneumobilia^*6)^	2	Cholecystolithiasis pneumobilia	C	Figures 54, 55
Debris echo (describe separately from stone image) ^*7)^	3	Biliary sludge	D2	Figures 56, 57
No abnormal findings	1	Normal gallbladder	A	
Extrahepatic bile duct				
Post-resection^*8)^	0	Post-extrahepatic bile duct resection	B	
Unable to visualize	0	Unable to visualize extrahepatic bile duct	D2	
Morphological abnormality				
Maximum diameter ≥ 8 mm, or ≥ 11 mm after cholecystectomy^*9)^	3	Bile duct dilatation	D2	
Without abnormal findings in the bile duct up to the near-papillary region	2	Bile duct dilatation	C	Figure 58
Cystic or fusiform shape	4	Pancreaticobiliary maljunction suspected	D2	Figures 59, 60
Wall thickening				
Maximum wall thickness ≥ 3 mm or internal hypoechoic layer is present in a part of the wall	3	Bile duct wall thickening	D2	Figure 61
Irregular mucosal surface	4	Bile duct tumor suspected	D2	
Irregular layered structure	5	Bile duct tumor	D1	Figure 62
Protruded or mass lesion (polyp)				
Protruded or mass lesion is present	4	Bile duct tumor suspected	D2	Figure 63
With irregularity or disruption of the layered structure of the attached wall	5	Bile duct tumor	D1	Figure 64 (Fig. 57)
Other findings				
Stone image (including calcified lesion)	2	Bile duct stone	D1	Figures 65, 66
Pneumobilia^*6)^	2	Pneumobilia	B	
Debris echo (describe separately from stone image) ^*10)^	3	Extrahepatic biliary sludge	D2	Figure 67
No abnormal findings	1	Normal extrahepatic bile duct	A	

^*1)^ If there is a remaining portion (e.g., gallbladder/bile duct), evaluate the ultrasound imaging findings of the remaining portion

^*2)^ Including cases where the wall cannot be evaluated due to atrophy or gallstones, and cases where the postprandial gallbladder wall cannot be evaluated

^*3)^ Confirm the absence of a cause of obstruction in the distal bile duct and pancreatic head

^*4)^ Pay attention to the presence of protruded lesions in cases with wall thickening with a small cystic structure or comet-like echo

^*5)^ Note that measured values are not what is assessed in the case of localized wall thickening

^*6)^ Pneumobilia and calcification/lithiasis should be differentiated by movement status at position change or while breathing

^*7)^ Confirm the absence of a cause of obstruction in the distal bile duct and pancreatic head

^*8)^ Document the resection site if known and evaluate the ultrasound imaging findings of the remaining portion

^*9)^ Measure from the beginning of the anterior wall echo to the beginning of the posterior wall echo of the bile duct on an enlarged image, and display in millimeters after rounding off to the nearest integer (Fig. 36)

^*10)^ Confirm the absence of a cause of obstruction in the distal bile duct and pancreas head (Fig. 68)

**Fig. 36:**
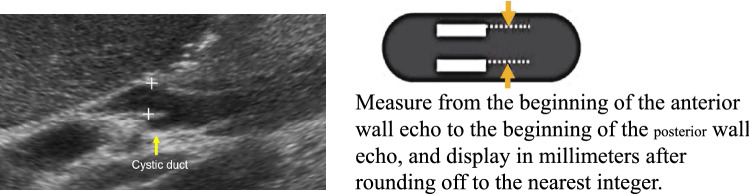
Measurement of bile duct diameter

**Fig. 37:**
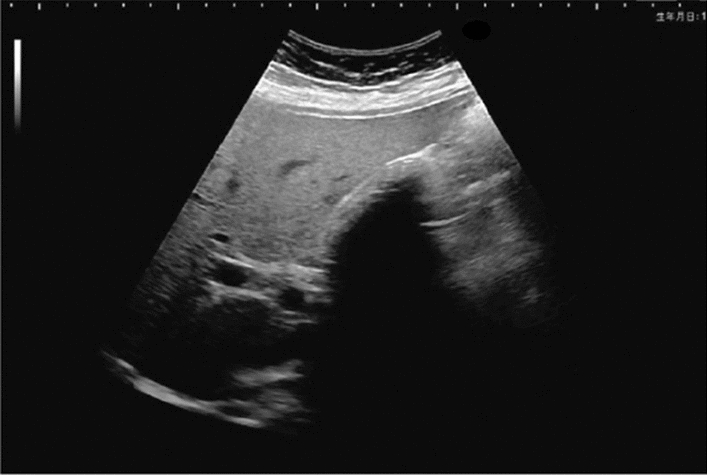
The gallbladder wall is inevaluable (the wall is inevaluable due to being filled with stones). Category 3, Assessment D2

**Fig. 38:**
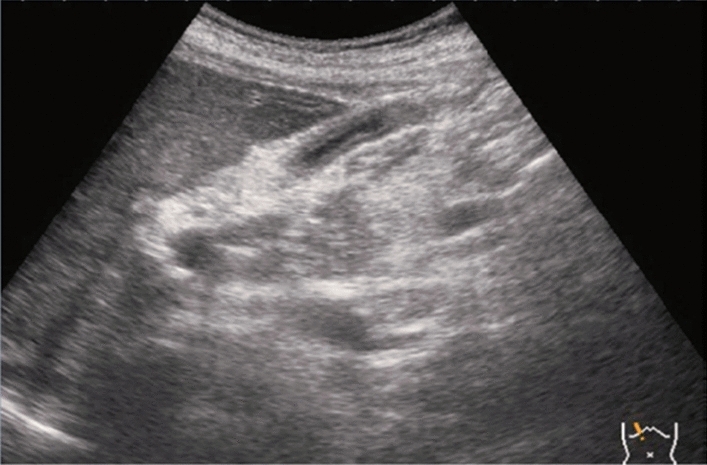
The gallbladder wall is inevaluable (the wall is inevaluable due to food intake). Category 3, Assessment D2

**Fig. 39:**
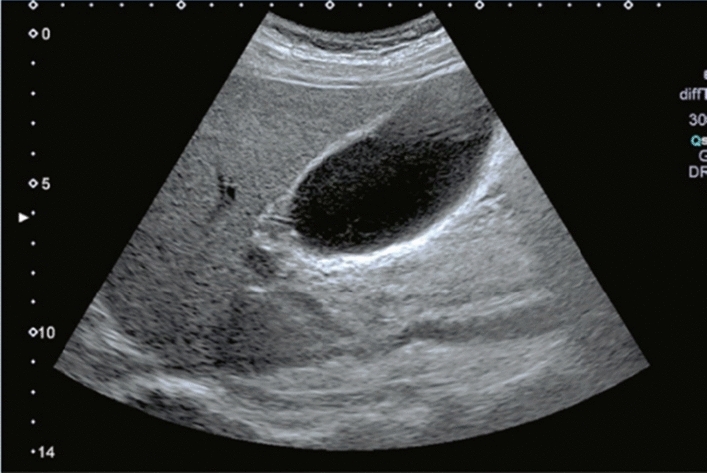
Gallbladder distension. Maximum short diameter ≥ 36 mm. Category 3, Assessment D2

**Fig. 40:**
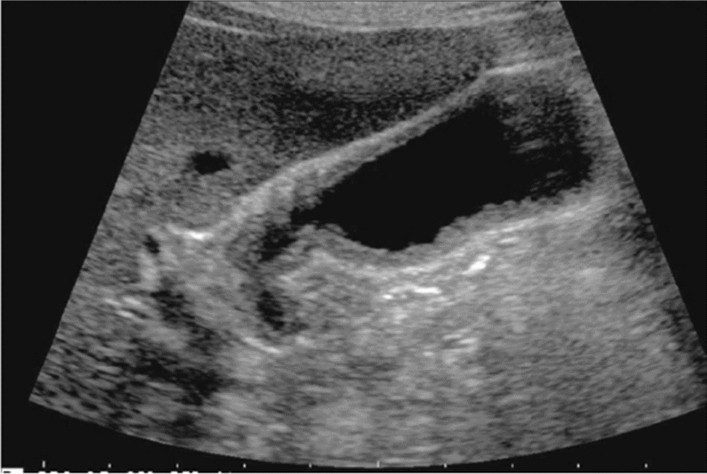
Wall thickening. Diffuse wall thickening (without small cystic structure or comet-like echo). Category 3, Assessment D2

**Fig. 41:**
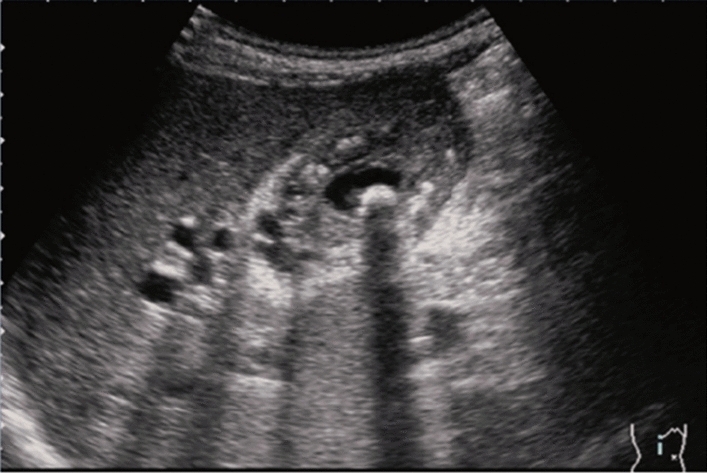
Wall thickening. Diffuse wall thickening (with cholecystolithiasis and small cystic structure or comet-like echo). Category 2, Assessment C

**Fig. 42:**
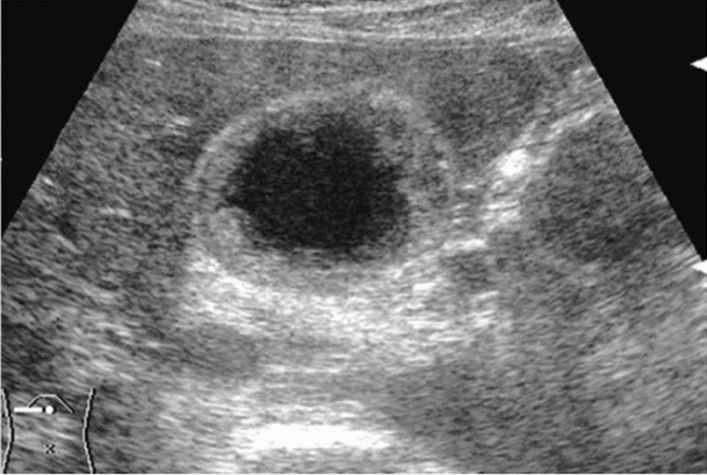
Wall thickening. Diffuse wall thickening (with irregularity of the layered structure of the wall and disruption of the outermost hyperechoic layer). Category 4, Assessment D2

**Fig. 43:**
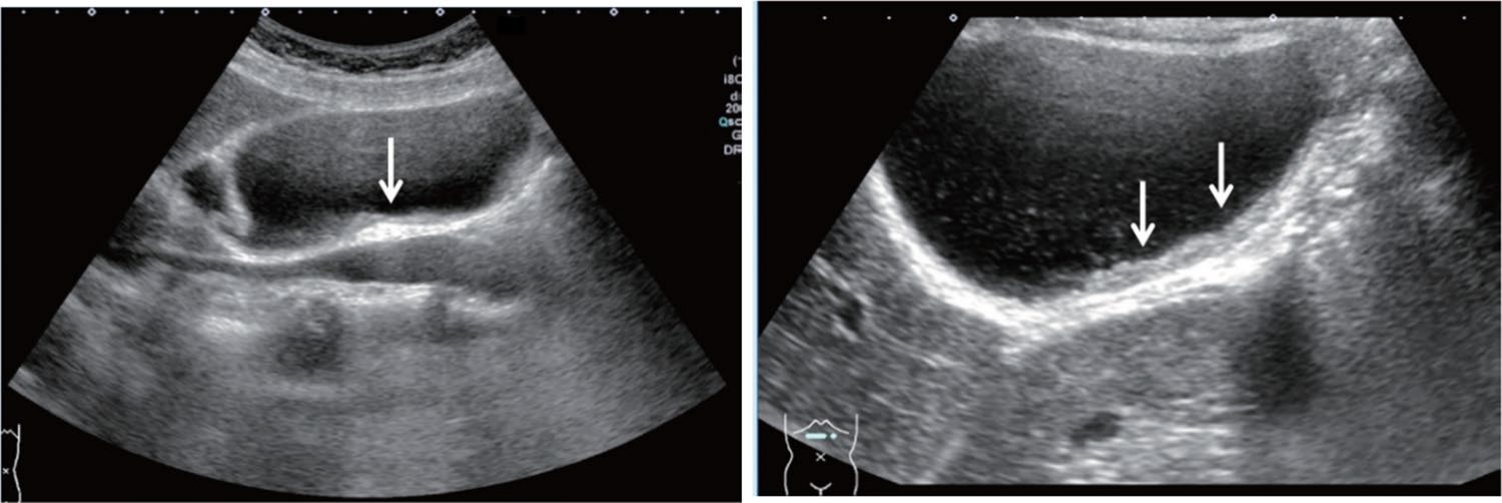
Wall thickening. Localized wall thickening (without small cystic structure or comet-like echo). Left: convex probe; right: high-frequency probe. Category 4, Assessment D2

**Fig. 44:**
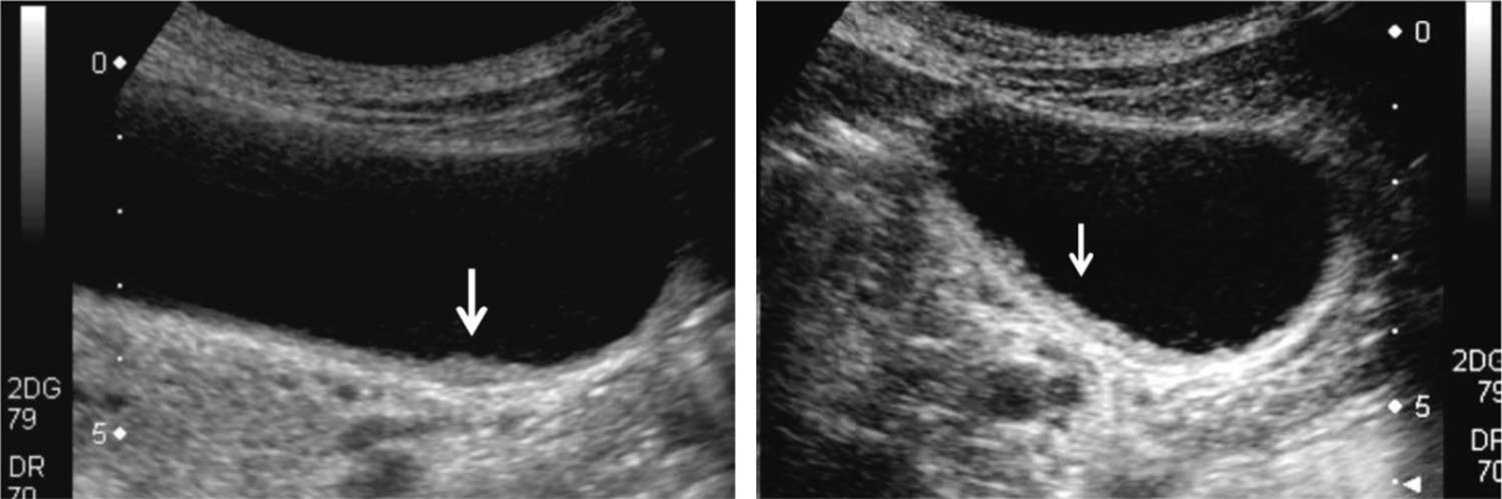
Wall thickening. Localized wall thickening (mimicking debris echo without small cystic structure or comet-like echo). Left: long-axis view; right: short-axis view. Category 4, Assessment D2

**Fig. 45:**
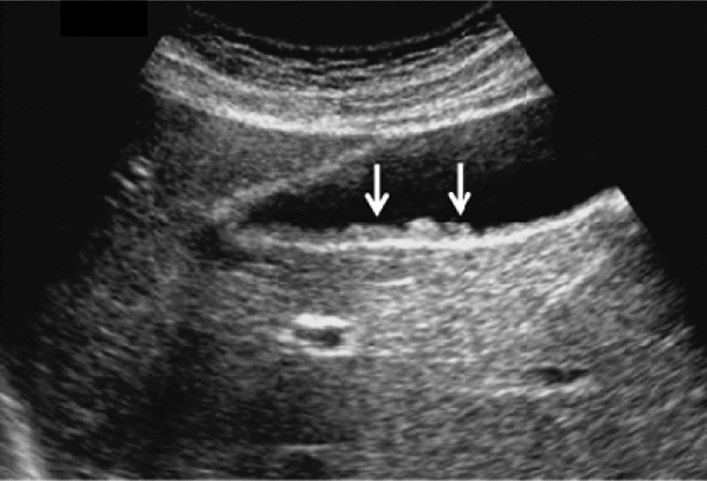
Wall thickening. Localized wall thickening (papillary without small cystic structure or comet-like echo). Category 4, Assessment D2

**Fig. 46:**
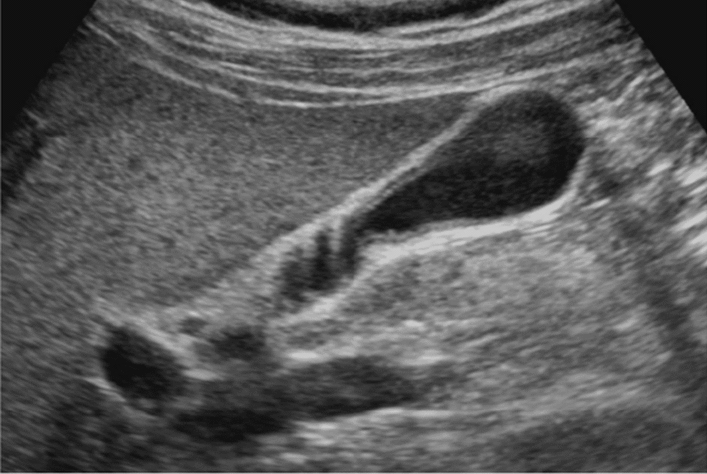
Wall thickening. Localized wall thickening (with comet-like echo). Category 2, Assessment C

**Fig. 47:**
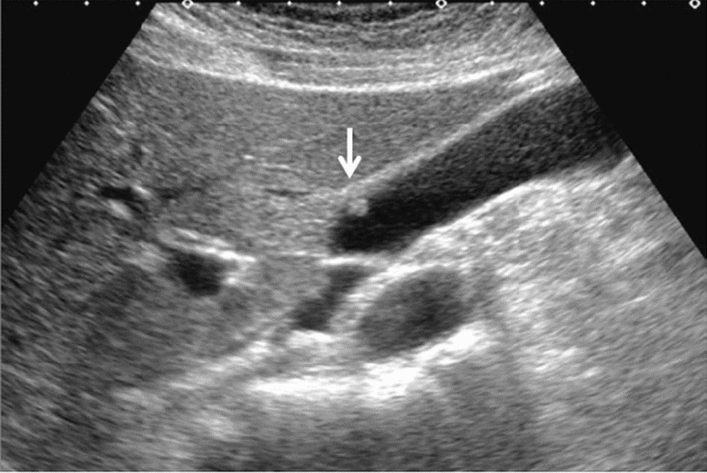
Protruded or mass lesion (polyp). Pedunculated (maximum diameter < 5 mm). Category 2, Assessment B

**Fig. 48:**
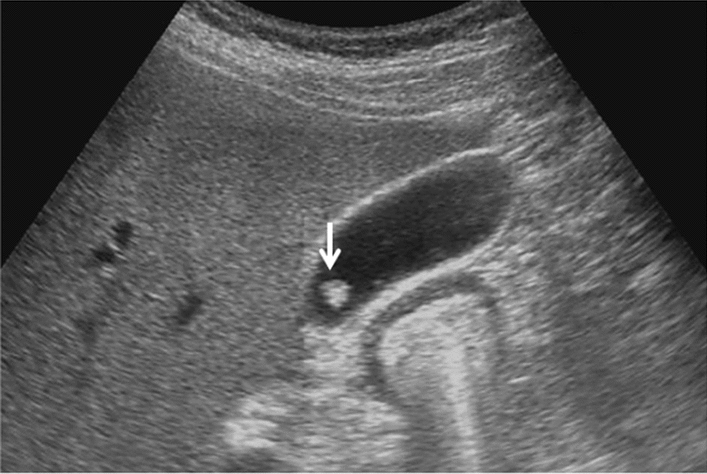
Protruded or mass lesion (polyp). Pedunculated [maximum diameter ≥ 5 mm, < 10 mm (mulberry-like)]. Category 2, Assessment B

**Fig. 49:**
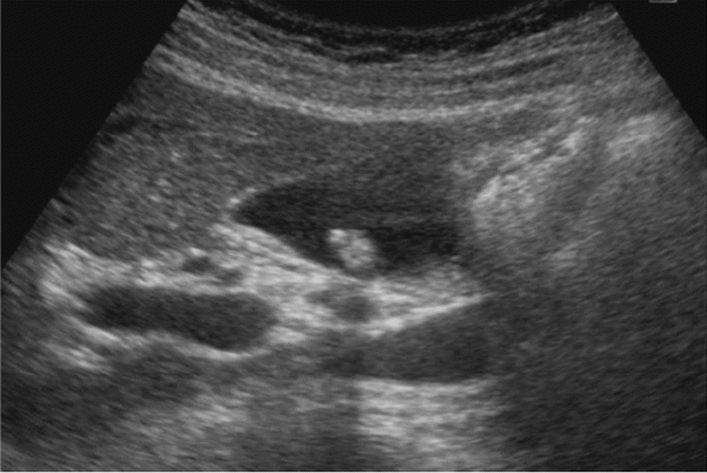
Protruded or mass lesion (polyp). Pedunculated [maximum diameter ≥ 5 mm, < 10 mm (with hyperechoic spot)]. Category 2, Assessment B

**Fig. 50:**
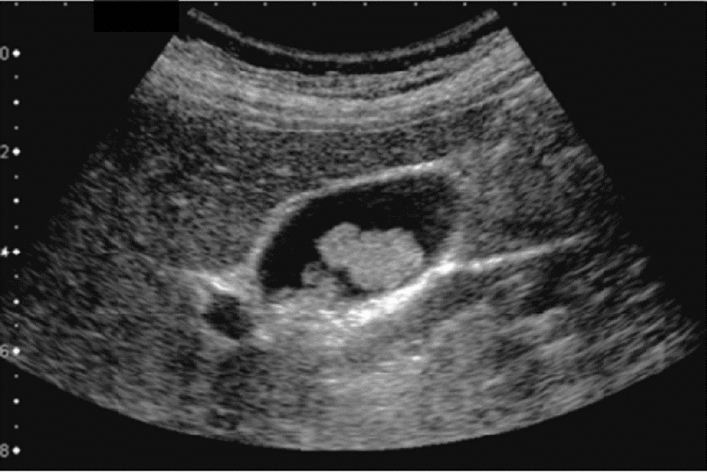
Protruded or mass lesion (polyp). Pedunculated (maximum diameter ≥ 10 mm). Category 4, Assessment D2

**Fig. 51:**
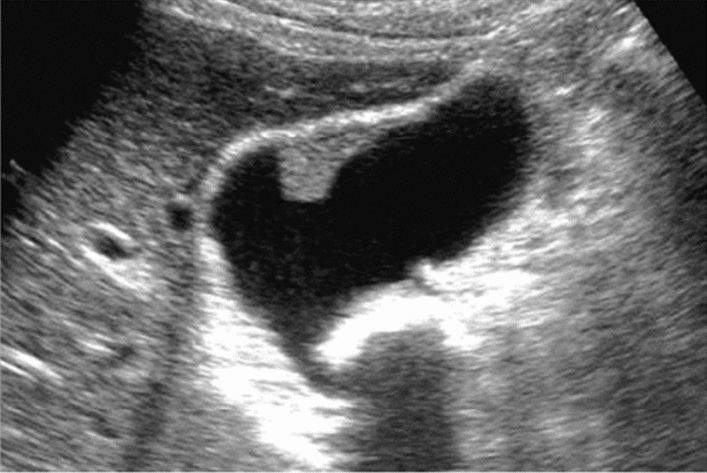
Protruded or mass lesion (polyp). Sessile (non-pedunculated) (without tear of the layered structure of the attached wall). Category 4, Assessment D2

**Fig. 52:**
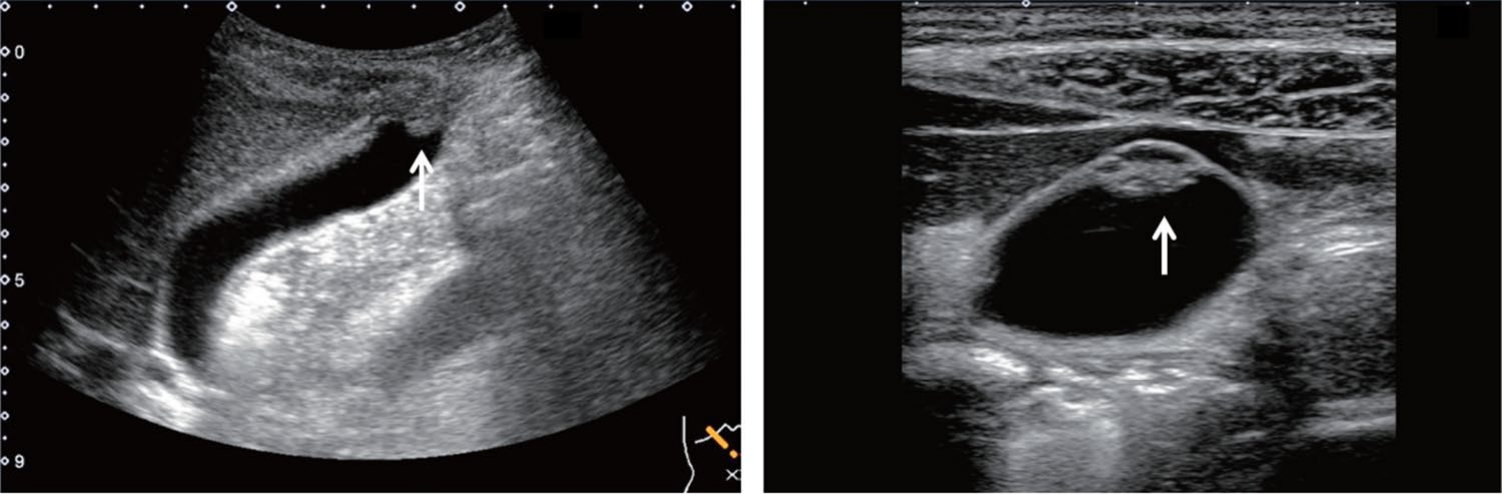
Protruded or mass lesion (polyp). Sessile (non-pedunculated) (with small cystic structures). Left: convex probe; right: high-frequency probe. Category 2, Assessment C

**Fig. 53:**
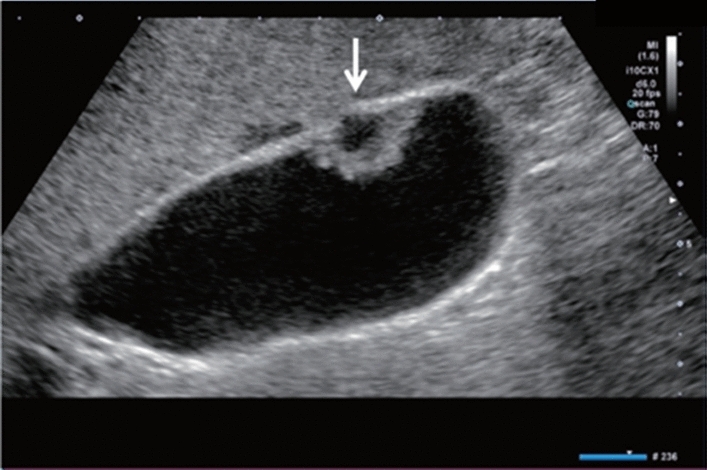
Protruded or mass lesion (polyp). Sessile (broad-based) (with tear of the layered structure of the attached wall). Category 5, Assessment D1

**Fig. 54:**
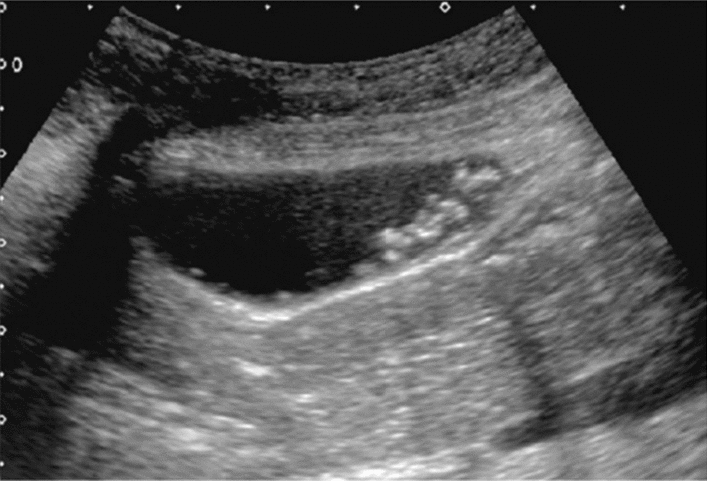
Other findings. Stone image (strong echo without wall thickening). Category 2, Assessment C

**Fig. 55:**
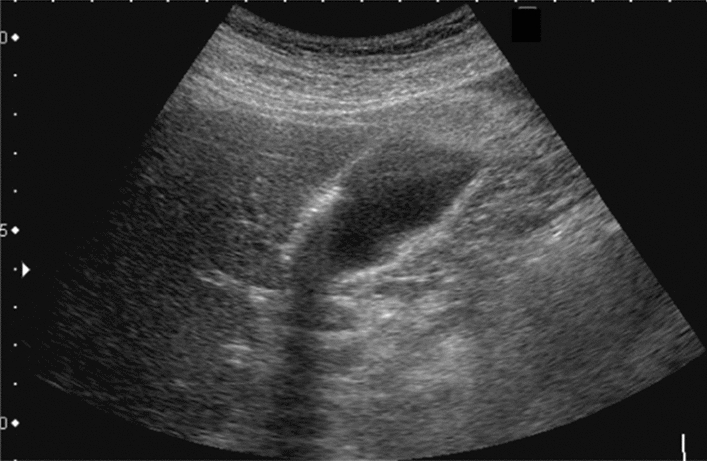
Other findings. Pneumobilia (movable linear strong echo with position change). Category 2, Assessment C

**Fig. 56:**
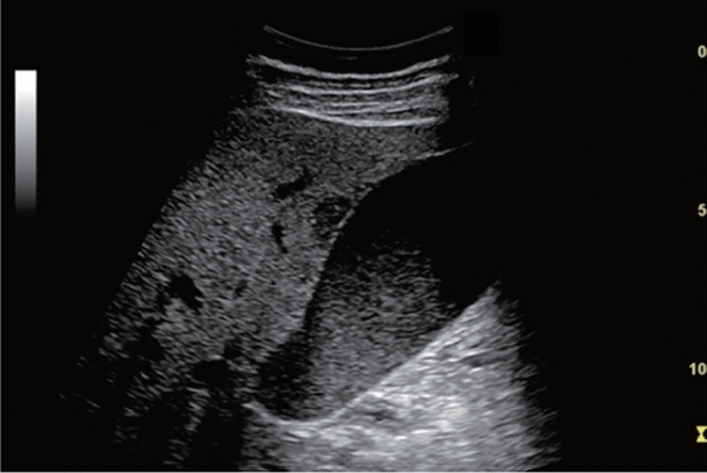
Other findings. Debris echo (gallbladder). Category 3, Assessment D2

**Fig. 57:**
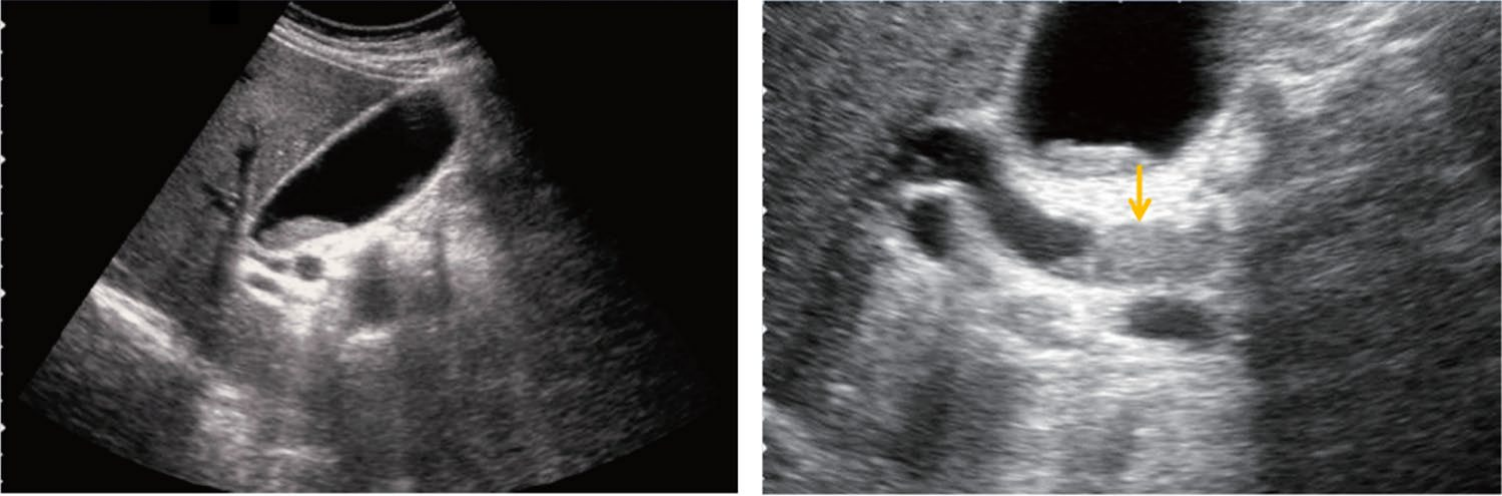
Other findings. Debris echo (mass lesion with the irregular outermost hyperechoic layer of the attached wall of the extrahepatic bile duct) (left: gallbladder, right: extrahepatic bile duct). Gallbladder is Category 3 and Assessment D2, but extrahepatic bile duct is Category 5 and Assessment D1

**Fig. 58:**
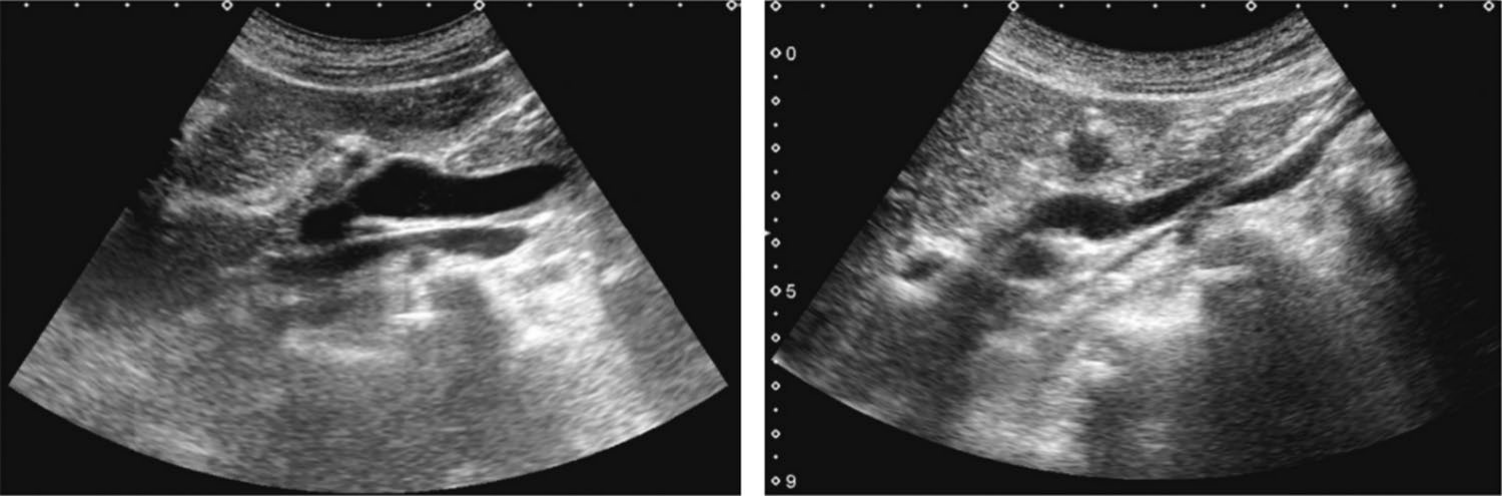
Extrahepatic bile duct. Morphological abnormality. (Dilatation of the perihilar extrahepatic bile duct is present, but abnormal findings up to the distal bile duct near the papillary edge are not present.) Left: Perihilar extrahepatic bile duct, Right: Distal extrahepatic bile duct. Category 2, Assessment C

**Fig. 59:**
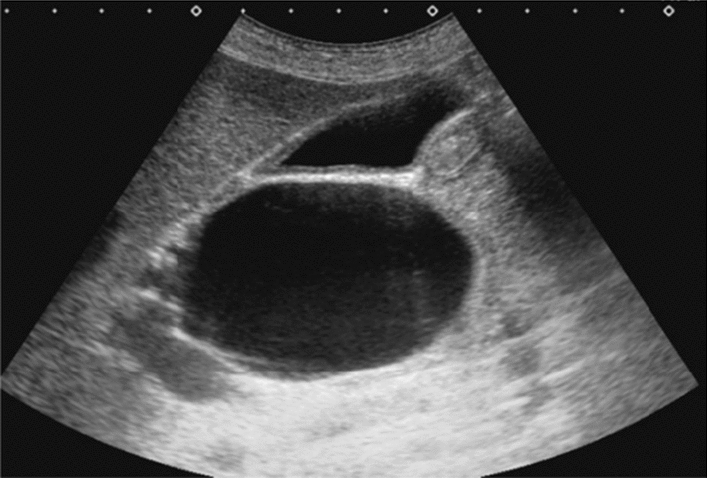
Extrahepatic bile duct. Morphological abnormality. Extrahepatic bile duct dilatation with cystic shape. Category 4, Assessment D2

**Fig. 60:**
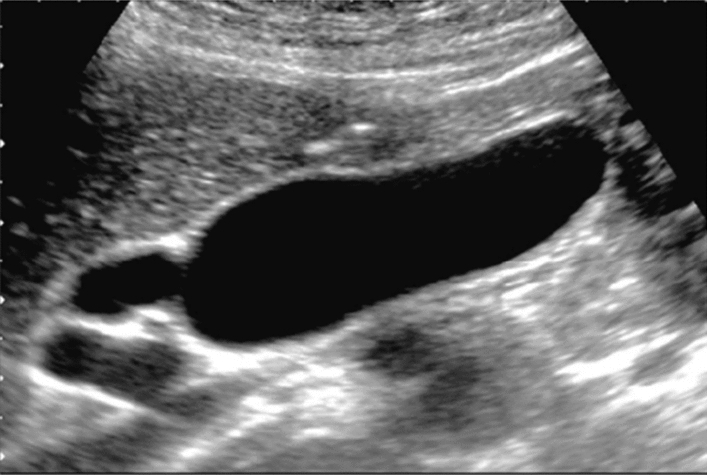
Extrahepatic bile duct. Morphological abnormality. Extrahepatic bile duct dilatation with fusiform shape. Category 4, Assessment D2

**Fig. 61:**
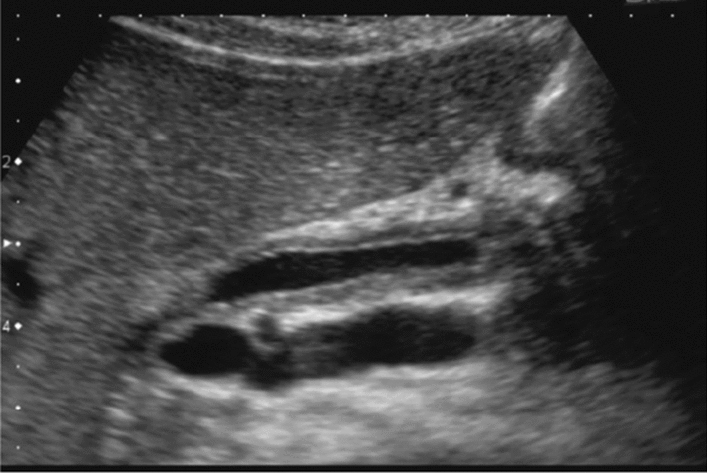
Wall thickening. Maximum wall thickness ≥ 3 mm (without irregularity of the mucosal surface and layered structure). Category 3, Assessment D2

**Fig. 62:**
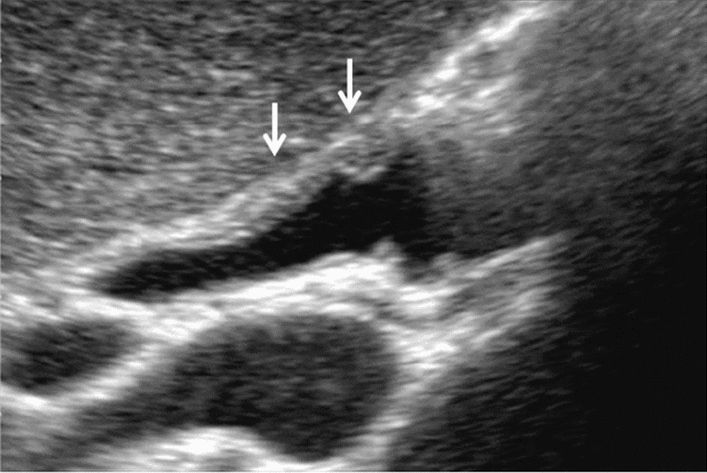
Wall thickening. Irregular layered structure (with irregularity of the mucosal surface and layered structure). Category 5, Assessment D1

**Fig. 63:**
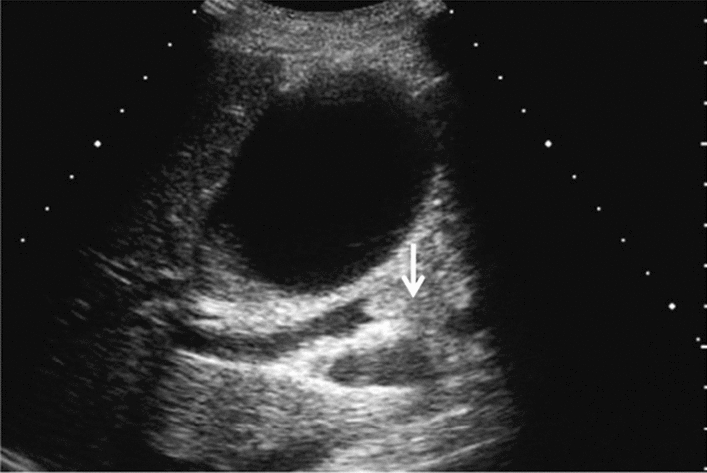
Protruded or mass lesion (polyp). Mass lesion (without disruption of the layered structure of the attached wall). Category 4, Assessment D2

**Fig. 64:**
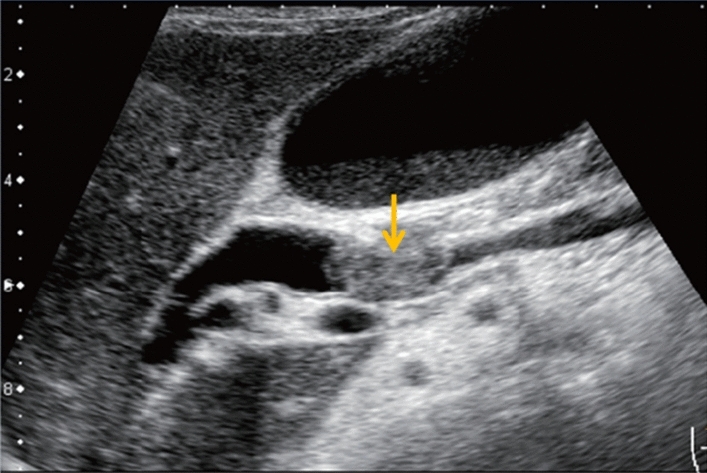
Protruded or mass lesion (polyp). Mass lesion (with disruption of the layered structure of the attached wall). Category 5, Assessment D1

**Fig. 65:**
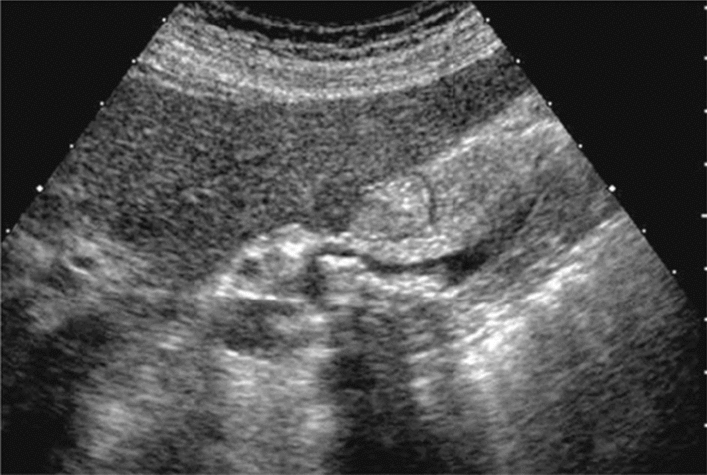
Other findings. Stone image (with acoustic shadow). Category 2, Assessment D1

**Fig. 66:**
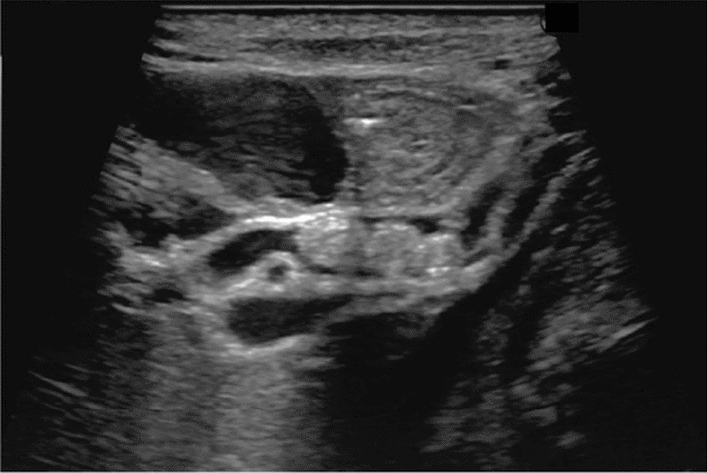
Other findings. Stone image (without acoustic shadow). Category 2, Assessment D1

**Fig. 67:**
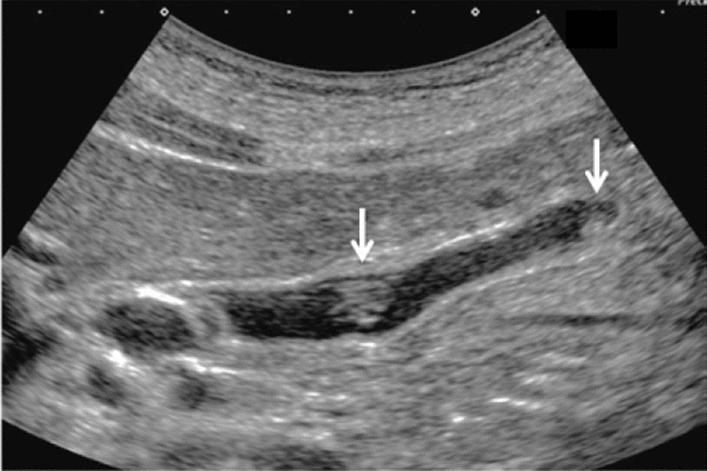
Other findings. Debris echo (with dilated extrahepatic bile duct). Category 3, Assessment D2

**Table 6:** Pancreas

Pancreas				
Ultrasound imaging finding	Category	Ultrasound finding (described in Report Form)	Assessment	Figure number
Post-resection^*1)^	0	Post-pancreatectomy	B	
Post-partial resection	2	Post-partial pancreatectomy	C	
Unable to visualize	0	Unable to visualize pancreas	D2	
Morphological abnormality				
Congenital deformity^*2)^	2	Pancreas deformity	B	
Maximum short diameter < 10 mm	2	Pancreatic atrophy	D2	Figure 69
Maximum short diameter ≥ 30 mm	2	Pancreatic enlargement	D2	Figure 70
Localized swelling^*3)^	2	Pancreas deformity	B	
Any one of decreased echo level, rough parenchymal echo pattern, or blurring of main pancreatic duct or vascular is present	4	Pancreatic tumor suspected	D2	Figures 71, 72
Main pancreatic duct dilatation				
Maximum diameter ≥ 3 mm at pancreatic body^*4)^	3	Pancreatic duct dilatation	D2	Figures 73, 74
Nodule present in the main pancreatic duct	4	Pancreatic tumor suspected	D2	Figure 75
Downstream (duodenal side) stenosis is present	4	Pancreatic tumor suspected	D2	Figure 76
Solid lesion^*5)^				Figures 77, 78
Hyperechoic mass lesion	2	Pancreatic mass	C	Figure 79
Maximum diameter ≥ 15 mm	3	Pancreatic mass	D2	Figure 80
Hypo(iso)echoic or mixed hyper- and hypoechoic mass lesion	4	Pancreatic tumor suspected	D2	Figures 81, 82
With disruption in any of the main pancreatic duct, extrahepatic bile duct, or peripancreatic blood vessels	5	Pancreatic tumor	D1	Figure 83
Cystic lesion (including branch dilatation)^*5)^				Figures 77, 78
Maximum diameter < 5 mm	2	Pancreatic cyst	B	
Maximum diameter ≥ 5 mm	3	Pancreatic cyst	D2	Figure 84
Solid component (mural nodules/wall thickening/septal thickening) and/or change in internal fluid (e.g., internal echogenic spots) is present^*6)^	4	Pancreatic cystic tumor	D2	Figures 85, 86, 87, 88
Other findings				
Calcified lesion^*7)^	2	Pancreatic stone or intrapancreatic calcification	C	Figures 89, 90
Vascular abnormality^*8)^	2	Pancreatic vascular abnormality	D2	Figure 91
No abnormal findings	1	Normal pancreas	A	

^*1)^ In the case of partial resection, document the resection site if known, and evaluate the ultrasound imaging findings of the remaining portion

^*2)^ For congenital deformities (e.g., pancreatic tail missing), evaluate ultrasound imaging findings at the remaining portion, and if there is no abnormality, regard as Category 2 and Assessment B

^*3)^ Lesions with an irregular contour are regarded as solid lesions, and only lesions with a smooth contour are regarded as localized swelling

^*4)^ Measure from the beginning of the anterior wall echo to the beginning of the posterior wall echo of the main pancreatic duct on a magnified image, and record in millimeters after rounding off to the nearest integer (Fig. 68)

^*5)^ Lesions with a mixture of solid and cystic components are included in solid or cystic lesions, depending on which component accounts for a greater proportion of the lesion

^*6)^ Changes in internal fluid (e.g., intracystic hemorrhage/infection) are also subject to thorough examination as the possibility of neoplasticity cannot be ruled out. In addition, neoplastic cysts, the generic term for cysts covered with cells showing neoplastic proliferation, are also included in this category

^*7)^ Calcification in the dilatated main pancreatic duct is Category 3 and Assessment D2, and calcified spot in a solid lesion is Category 4 and Assessment D2

^*8)^ Vascular abnormalities include vascular aneurysms, arterial-venous shunts (including arteriovenous malformation), venous emboli (thrombi), collateral flow, and so on. However, vascular abnormalities associated with solid lesions should be evaluated according to the criteria for solid lesion (Fig. 92)

**Fig. 68:**
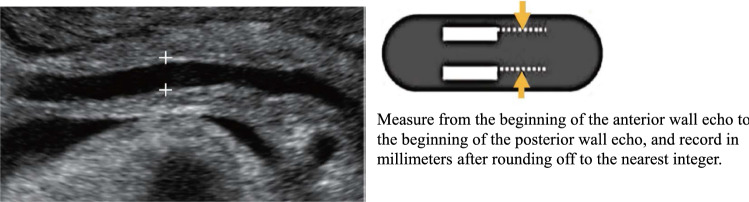
Measurement of main pancreatic duct diameter

**Fig. 69:**
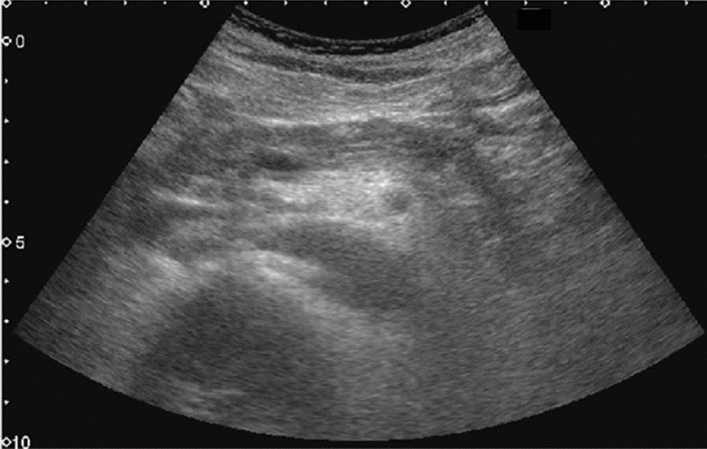
Morphological abnormality. Maximum short diameter < 10 mm. Category 2, Assessment D2

**Fig. 70:**
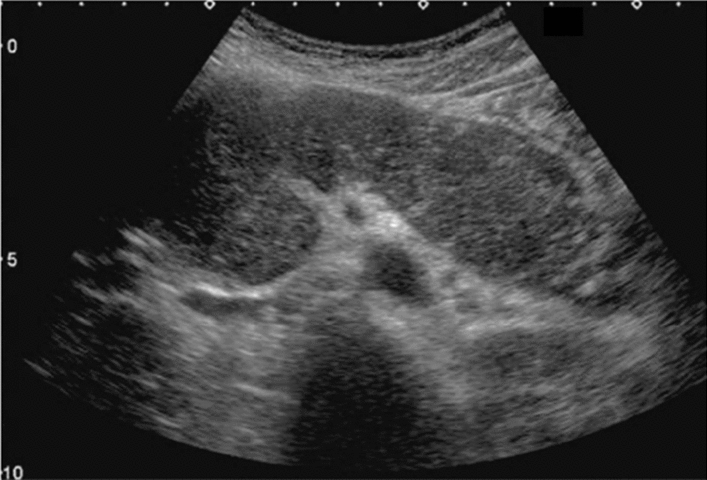
Morphological abnormality. Maximum short diameter ≥ 30 mm. Category 2, Assessment D2

**Fig. 71:**
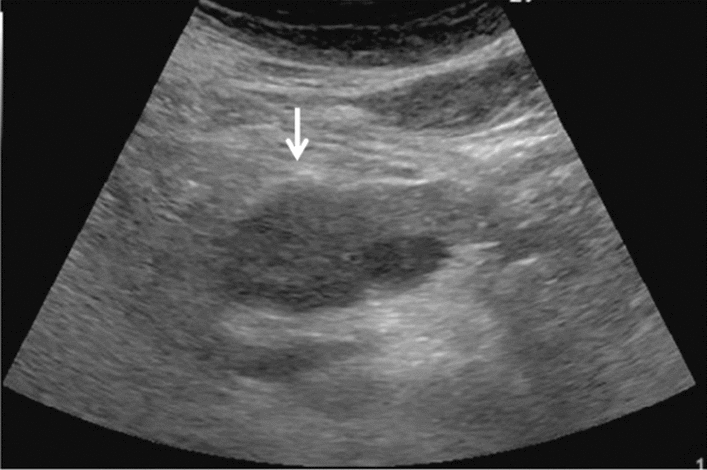
Morphological abnormality. Localized swelling (pancreatic head) (with decreased echo level). Category 4, Assessment D2

**Fig. 72:**
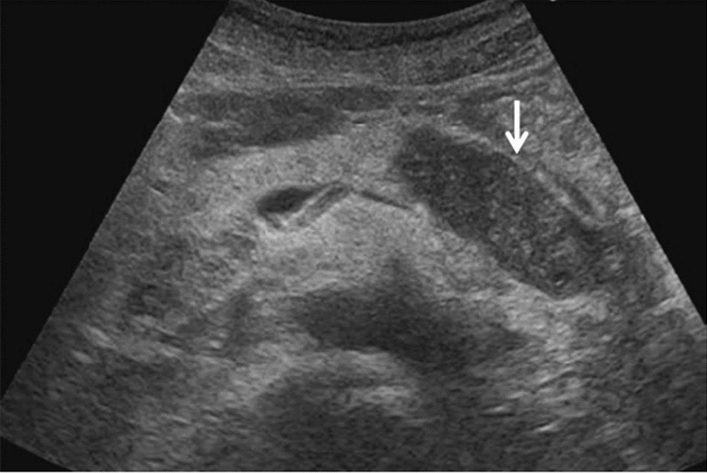
Morphological abnormality. Localized swelling (pancreatic tail) (with decreased echo level). Category 4, Assessment D2

**Fig. 73:**
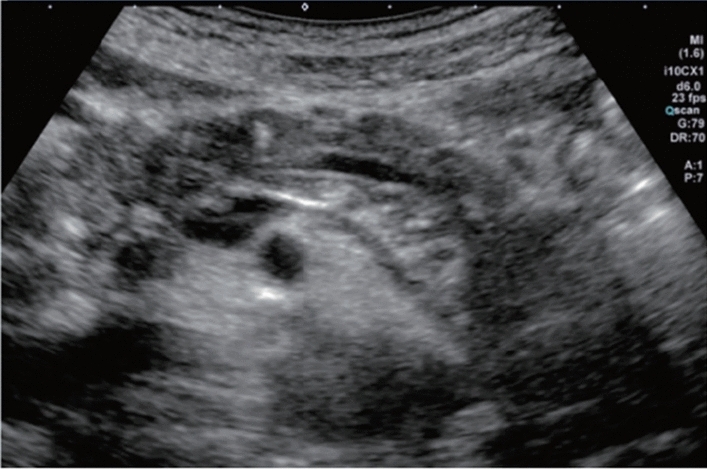
Main pancreatic duct dilatation. Maximum diameter ≥ 3 mm at pancreatic body (with calcification of pancreatic parenchyma). Category 3, Assessment D2

**Fig. 74:**
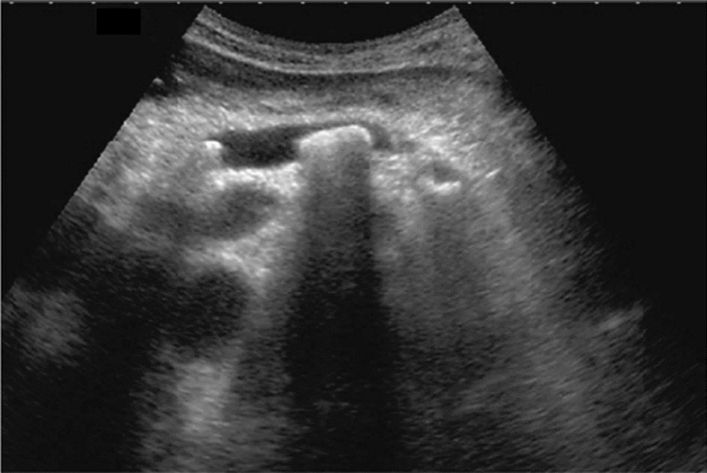
Main pancreatic duct dilatation. Maximum diameter ≥ 3 mm at pancreatic body (with calcification in the main pancreatic duct). Category 3, Assessment D2

**Fig. 75:**
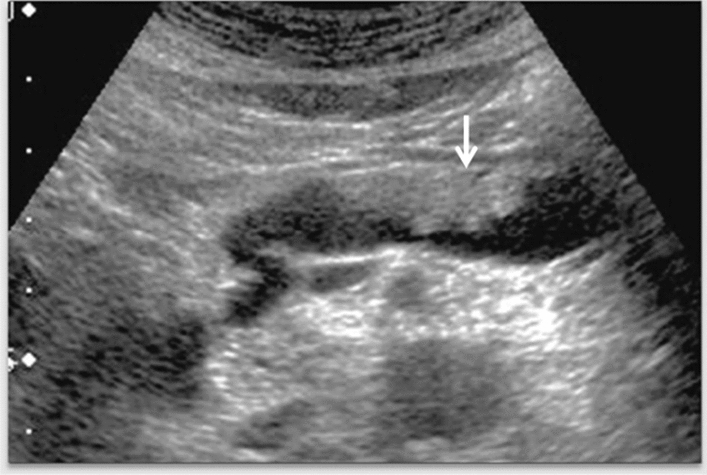
Main pancreatic duct dilatation. Maximum diameter ≥ 3 mm at pancreatic body (with papillary nodule in the main pancreatic duct). Category 4, Assessment D2

**Fig. 76:**
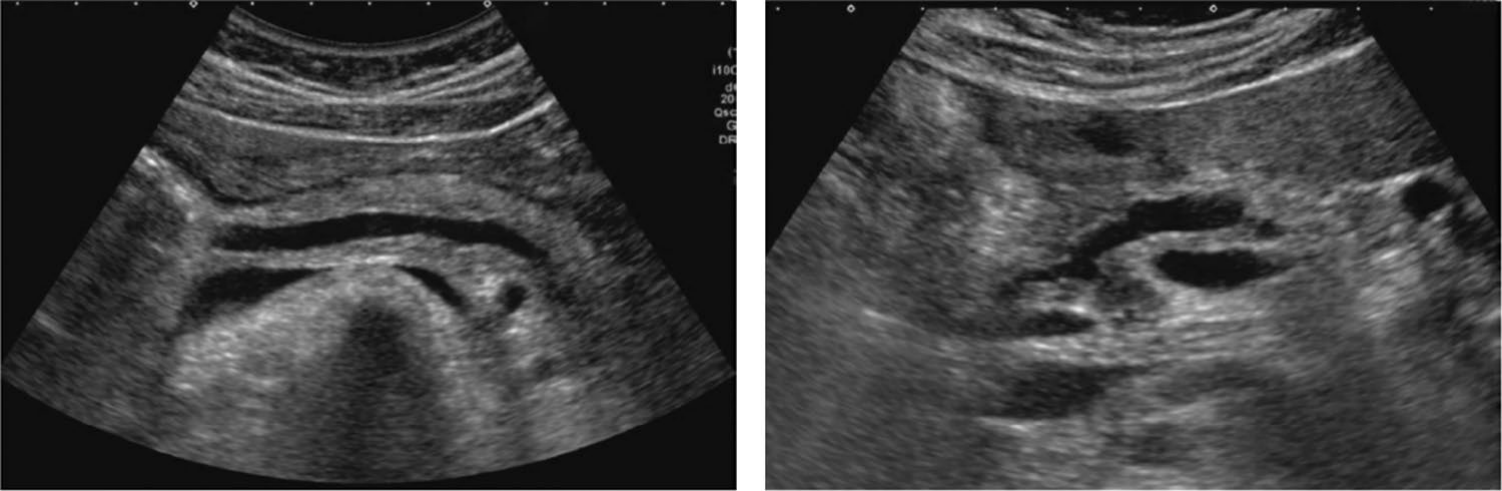
Main pancreatic duct dilatation. Maximum diameter ≥ 3 mm at pancreatic body (with downstream (duodenal side) stenosis). Left: pancreatic body; right: main pancreatic duct stenosis in the pancreatic head. Category 4, Assessment D2

**Fig. 77:**
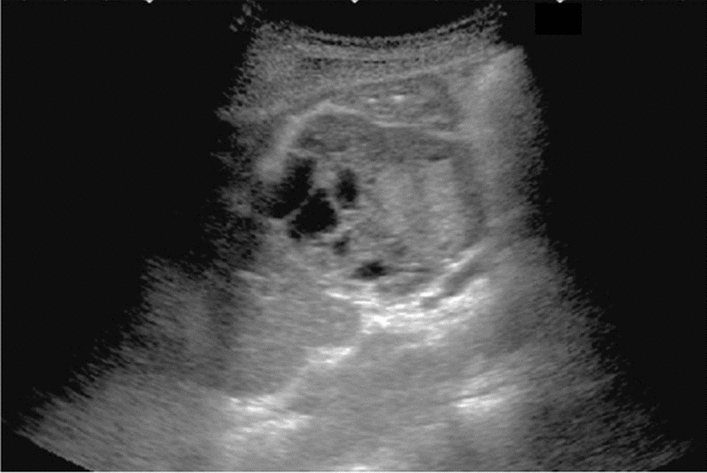
Solid lesion. Hypo(iso)echoic or mixed hyper- and hypoechoic mass lesion (lesion presenting a mixed pattern with a mixture of solid and cystic components). Category 4, Assessment D2

**Fig. 78:**
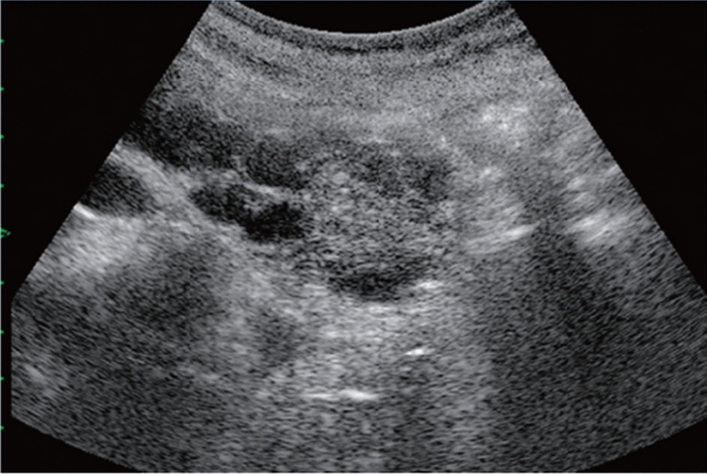
Cystic lesion. A solid component (mural nodules/wall thickening) is present (lesion presenting a mixed pattern with a mixture of solid and cystic components). Category 4, Assessment D2

**Fig. 79:**
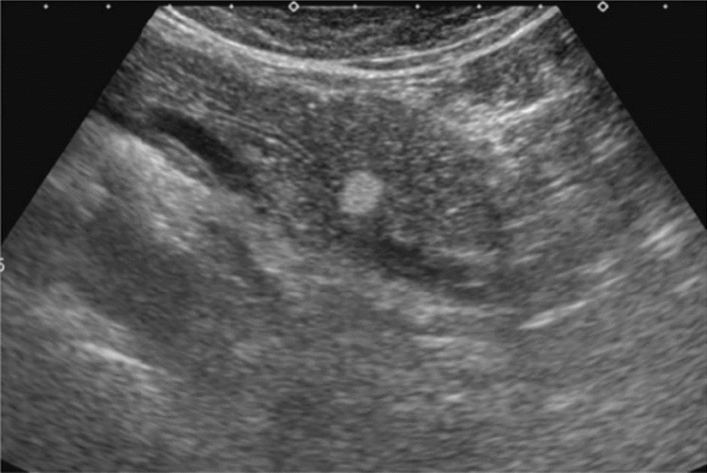
Solid lesion. Hyperechoic mass lesion (maximum diameter < 15 mm). Category 2, Assessment C

**Fig. 80:**
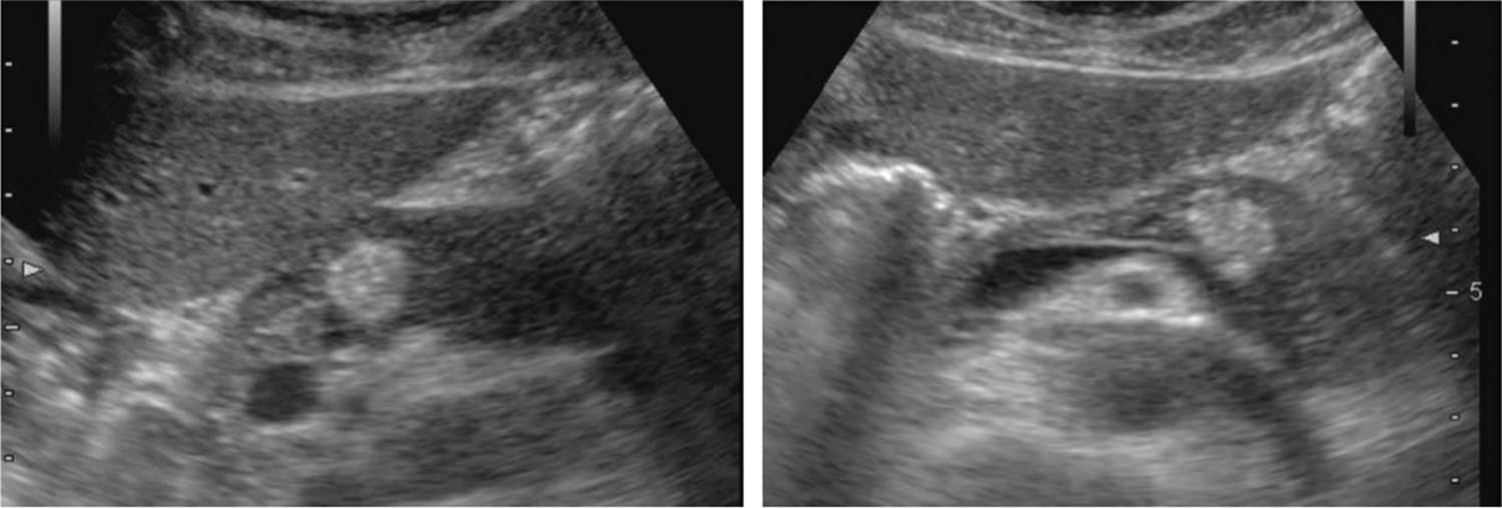
Solid lesion. Hyperechoic mass lesion (maximum diameter ≥ 15 mm). Category 3, Assessment D2

**Fig. 81:**
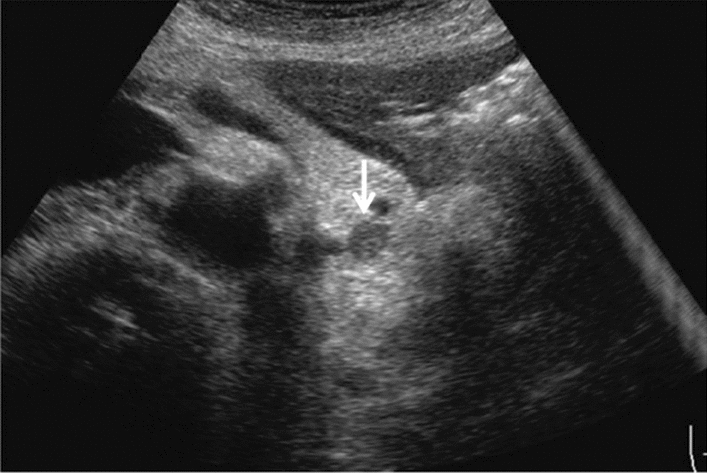
Solid lesion. Hypoechoic mass lesion. Category 4, Assessment D2

**Fig. 82:**
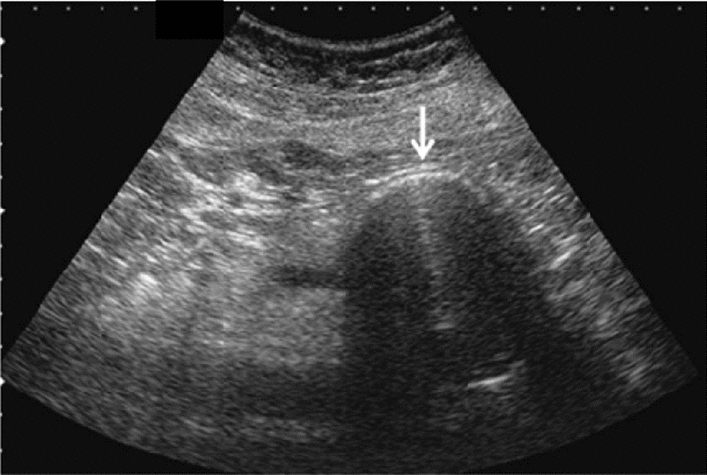
Solid lesion. Hypoechoic mass lesion (with eggshell-like calcification). Category 4, Assessment D2

**Fig. 83:**
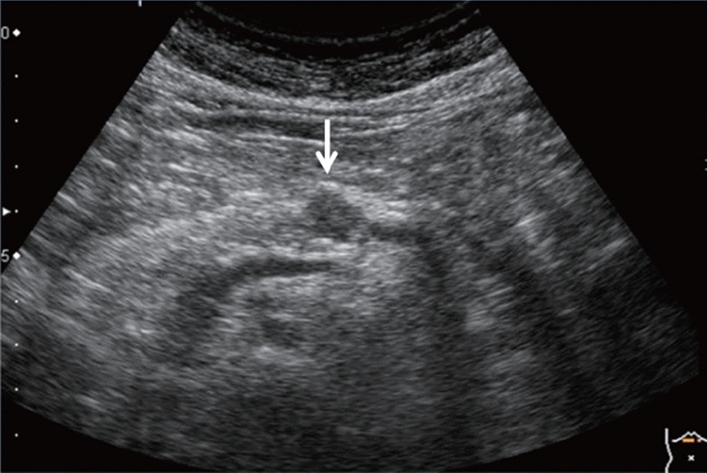
Solid lesion. Hypoechoic mass lesion with the disrupted main pancreatic duct (mass lesion with dilatation of caudal pancreatic duct). Category 5, Assessment D1

**Fig. 84:**
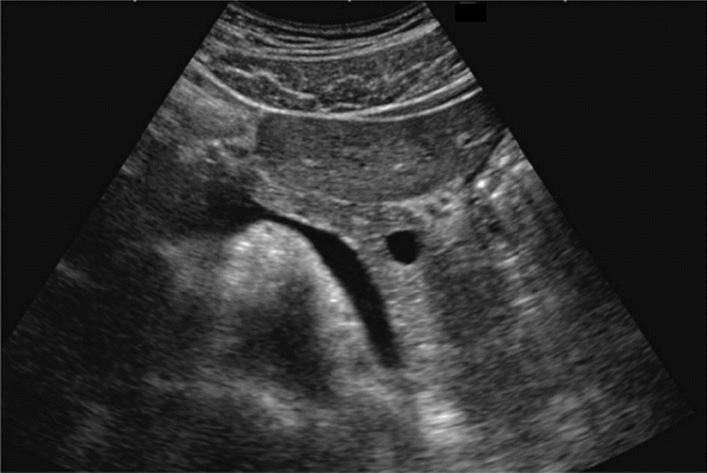
Cystic lesion (including branch dilatation). Maximum diameter ≥ 5 mm. Category 3, Assessment D2

**Fig. 85:**
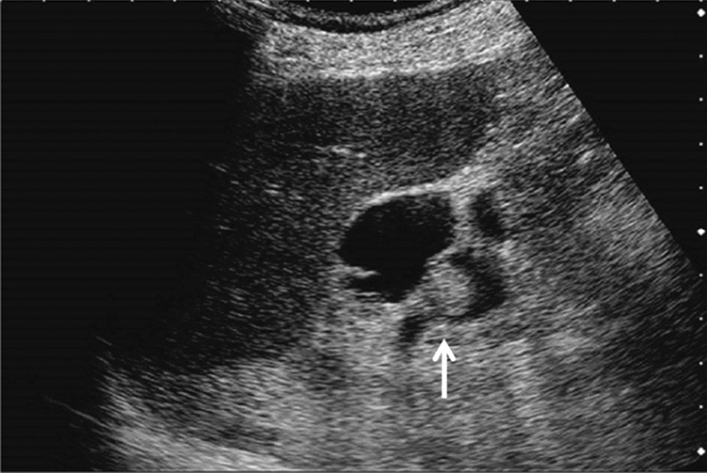
Cystic lesion (including branch dilatation). A solid component (mural nodules) is present. Category 4, Assessment D2

**Fig. 86:**
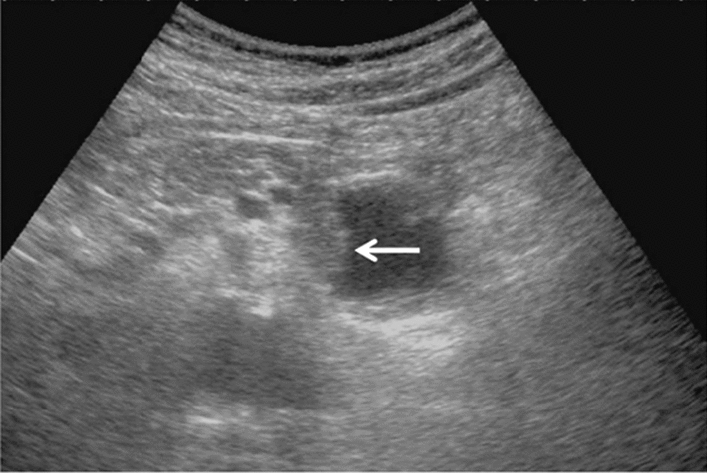
Cystic lesion (including branch dilatation). A solid component (wall thickening) is present. Category 4, Assessment D2

**Fig. 87:**
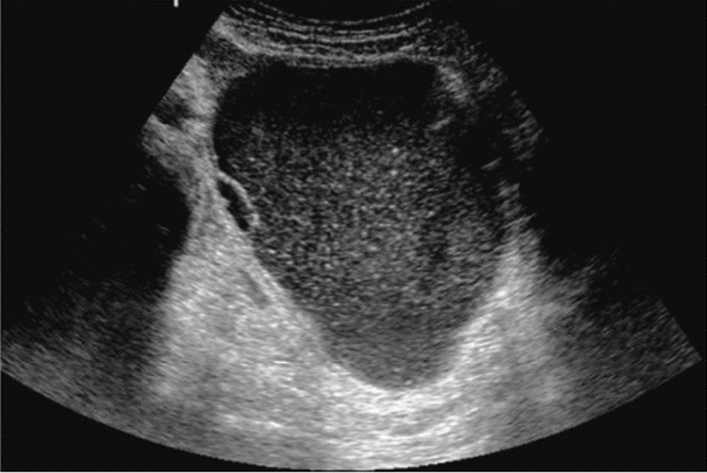
Cystic lesion (including branch dilatation). A change in internal fluid (internal echogenic spots) is present. Category 4, Assessment D2

**Fig. 88:**
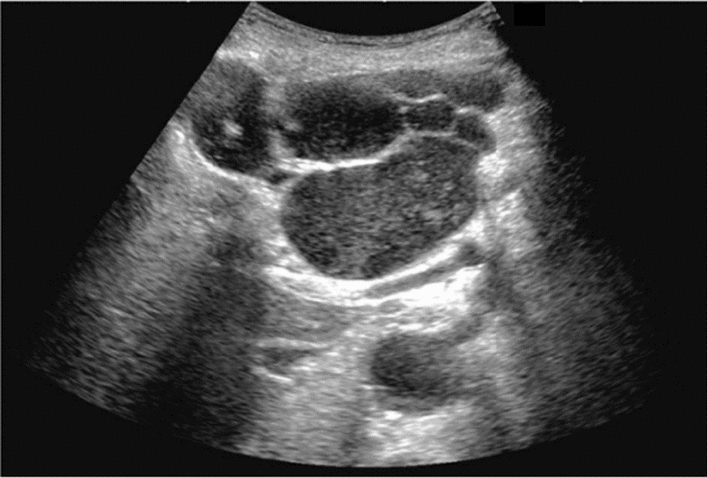
Cystic lesion (including branch dilatation). A change in internal fluid (internal echogenic spots) is present. Category 4, Assessment D2

**Fig. 89:**
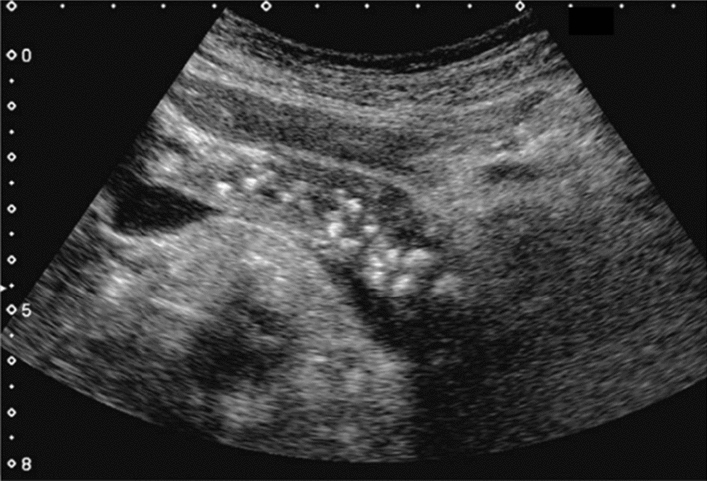
Other findings. Calcified lesion (calcification in the pancreatic body and tail). Category 2, Assessment C

**Fig. 90:**
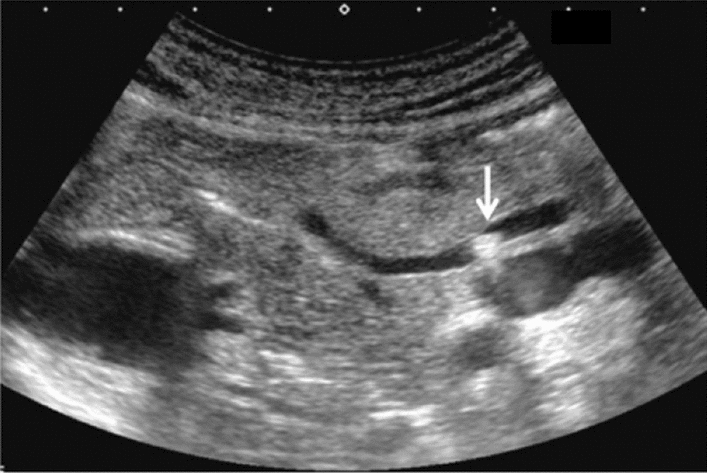
Other findings. Calcification lesion (calcification in the main pancreatic duct in the pancreatic head (without main pancreatic duct dilatation in the pancreatic body)). Category 2, Assessment C

**Fig. 91:**
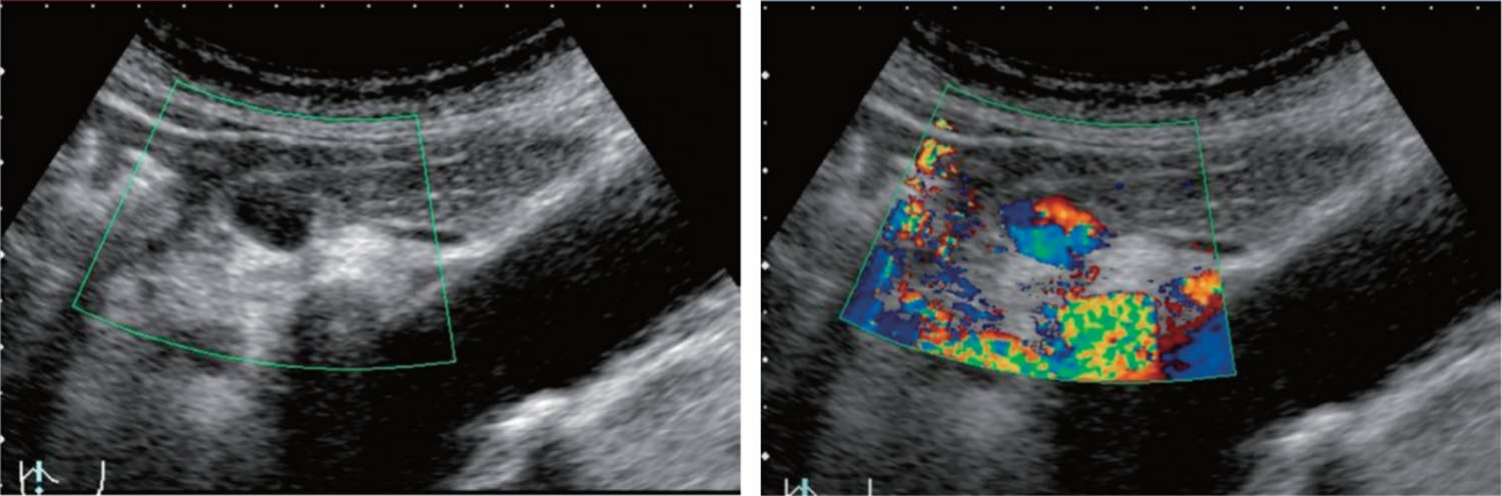
Other findings. Vascular abnormality (A cystic lesion is observed in the pancreas body by the B-mode, and an internal blood flow signal is present on color Doppler.). Left: B-mode; right: color Doppler. Category 2, Assessment D2

**Table 7:** Spleen

Spleen				
Ultrasound imaging finding	Category	Ultrasound finding (described in Report Form)	Assessment	Figure number
Post-resection^*1)^ Post-local treatment	0 2	Post-splenectomy Post-local spleen treatment	B C	Figure 93
Unable to visualize^*2)^	0	Unable to visualize spleen	B	
Morphological abnormality				
Congenital deformity^*3)^	2	Spleen deformity	B	
Maximum diameter ≥ 10 cm, < 15 cm^*4)^	2	Splenomegaly	B	
Maximum diameter ≥ 15 cm	3	Splenomegaly	D2	Figure 94
Solid lesion				
Hyperechoic mass image	3	Splenic mass	D2	Figure 95
Hypoechoic mass image	4	Splenic tumor suspected	D2	Figure 96
Hyper/hypoechoic mixed mass image	4	Splenic tumor suspected	D2	Figure 97
Central high echo	5	Splenic tumor	D1	Figure 98
Cystic lesion				
Cystic lesion (without the following findings regardless of the size)	2	Splenic cyst	B	
Solid component (mural nodules/wall thickening/septal thickening) and change in internal fluid (e.g., internal echogenic spots) are present^*5)^	4	Splenic cystic tumor suspected	D2	Figure 99
Other findings				
Calcified lesion	2	Intrasplenic calcification	B	Figure 100
Vascular abnormality^*6)^	2	Spleen vascular abnormality	D2	Figure 101
Solid lesion in the splenic hilum	3	Mass in the splenic hilum	D2	Figure 102
Image of oval mass with homogeneous internal echo at echo level equal to that of the spleen	2	Accessory spleen	B	Figure 103
No abnormal findings	1	Normal spleen	A	

^*1)^ In the case of partial resection, document the resection site if known, and evaluate the remaining portion using the same method as others

^*2)^ Confirm the presence or absence of excision, and regard as unable to visualize if the presence or absence of swelling cannot be assessed, but there is no need for thorough examination

^*3)^ Regard a congenital deformity (e.g., polysplenia) as Category 2 and Assessment B, and evaluate the remaining portion using the same method as others

^*4)^ With respect to the size of the spleen, the reference range differs depending on the age and build of the examinee

^*5)^ All cystic lesions with definite wall thickness should be regarded as wall thickening. In addition, changes in internal fluid (e.g., intracystic hemorrhage/infection) are regarded as Category 4 and Assessment D2 as the possibility of a cystic tumor cannot be ruled out (Fig. 104)

^*6)^ In addition to aneurysms, this includes abnormalities of the splenic hilum such as collateral flow of the splenic vein. However, mass lesion-related vascular abnormalities should be evaluated in the same manner as mass lesions. The measured value should be the maximum diameter (Fig. 92)

**Fig. 92:**
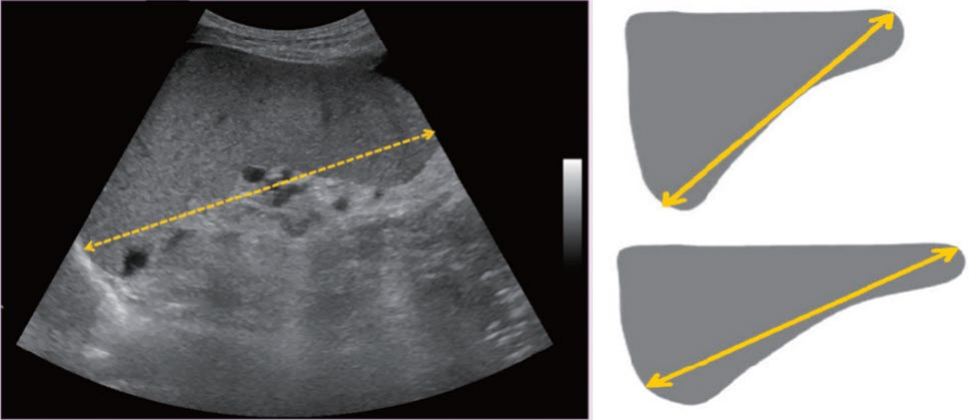
Measurement of spleen diameter. Visualize the spleen in the long-axis view and measure the maximum diameter

**Fig. 93:**
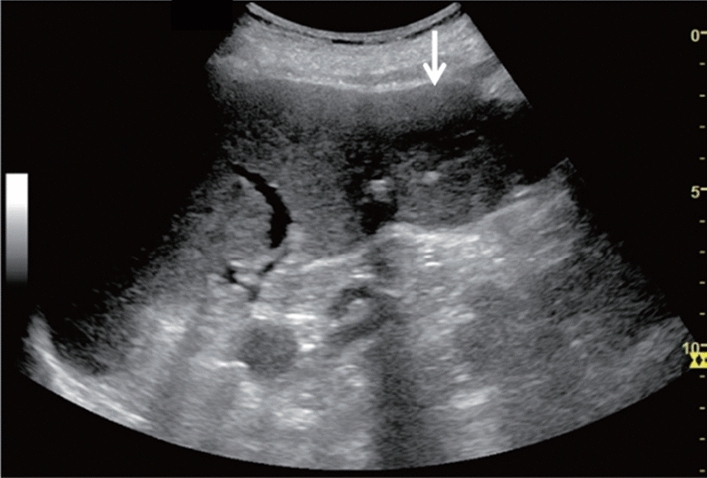
Post-local treatment (post-splenic embolization). Category 2, Assessment C

**Fig. 94:**
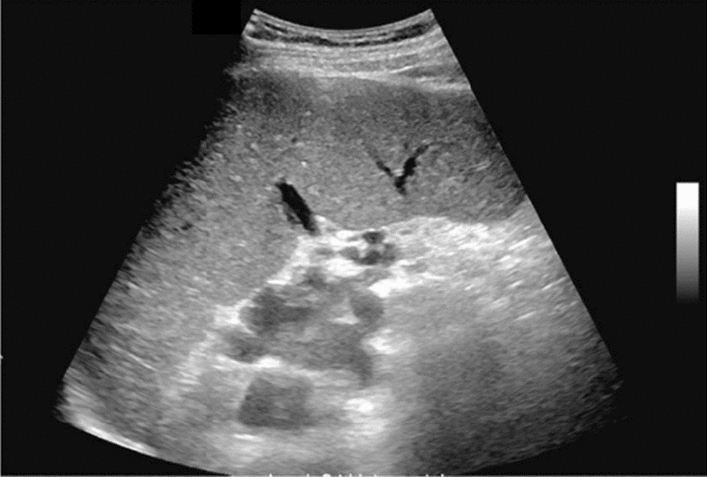
Malformation. Maximum diameter ≥ 15 cm. Category 3, Assessment D2

**Fig. 95:**
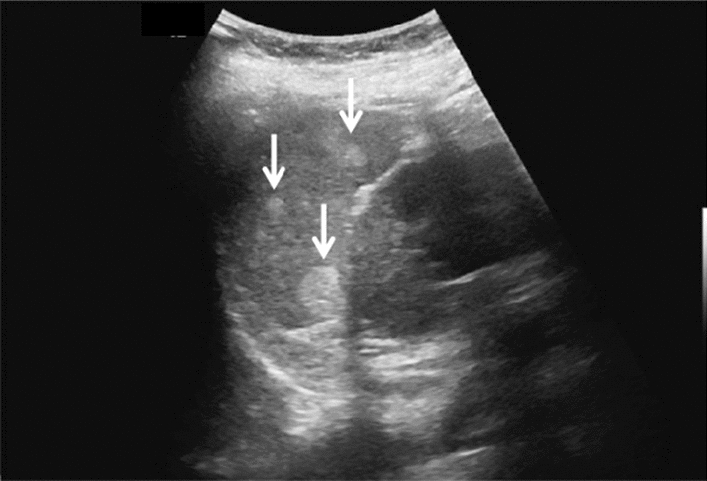
Solid lesion. Hyperechoic mass image. Category 3, Assessment D2

**Fig. 96:**
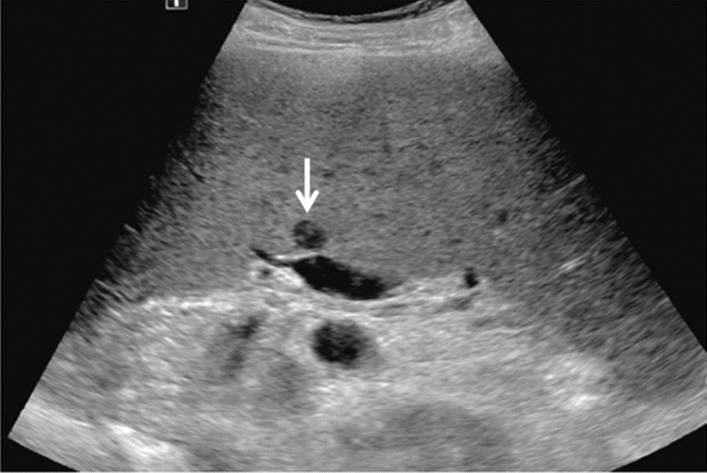
Solid lesion. Hypoechoic mass image. Category 4, Assessment D2

**Fig. 97:**
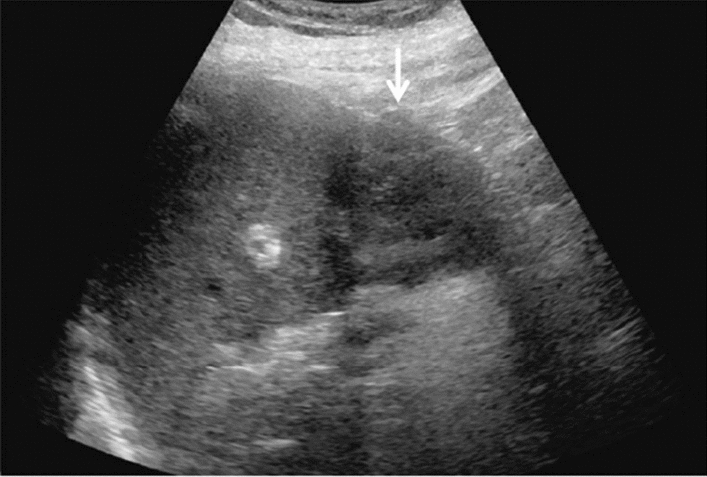
Solid lesion. Hyper/hypoechoic mixed mass image. Category 4, Assessment D2

**Fig. 98:**
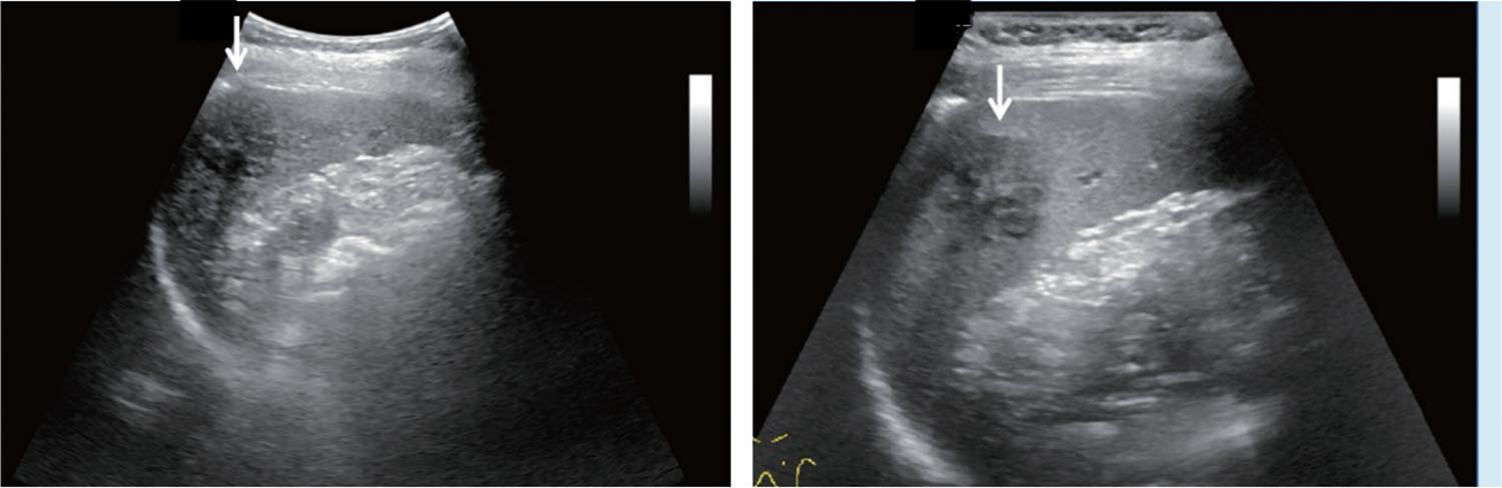
Solid lesion. Central hyperechoic mass image. Category 5, Assessment D1

**Fig. 99:**
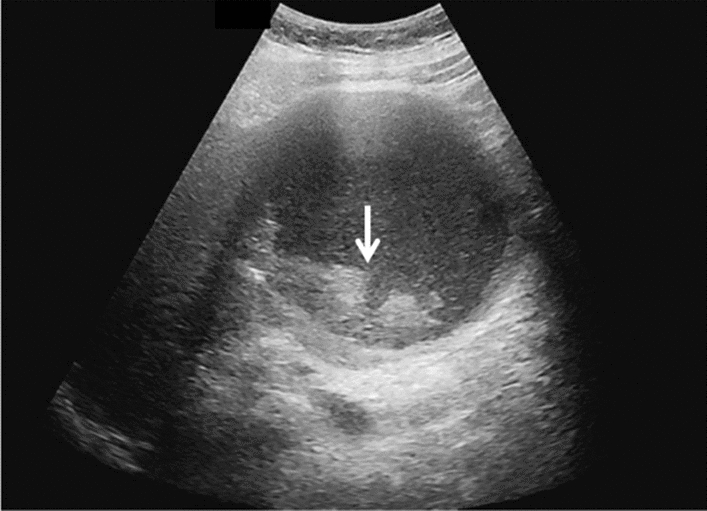
Cystic lesion. A solid component (intracystic nodules/wall thickening/internal echogenic spots) is present. Category 4, Assessment D2

**Fig. 100:**
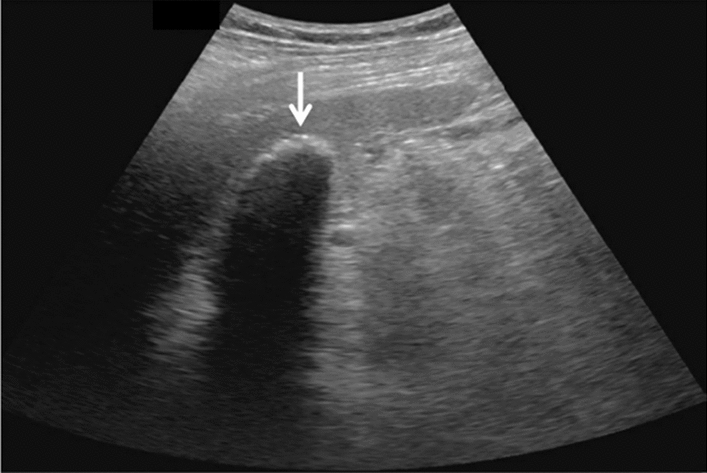
Other findings. Calcified opacity. Category 2, Assessment B

**Fig. 101:**
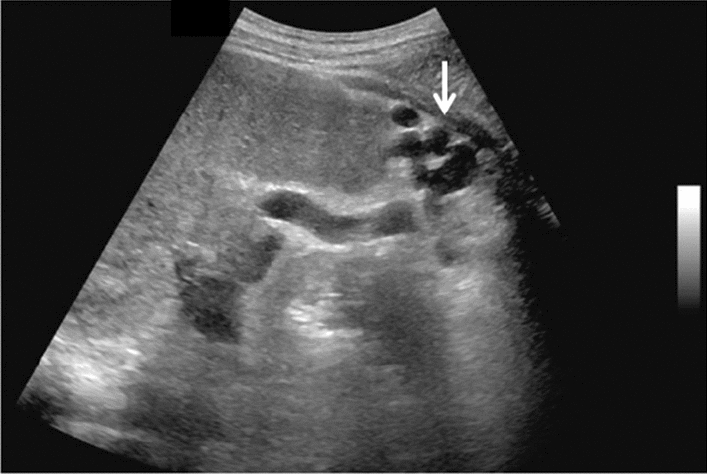
Other findings. Vascular abnormality (collateral flow of splenic vein). Category 2, Assessment D2

**Fig. 102:**
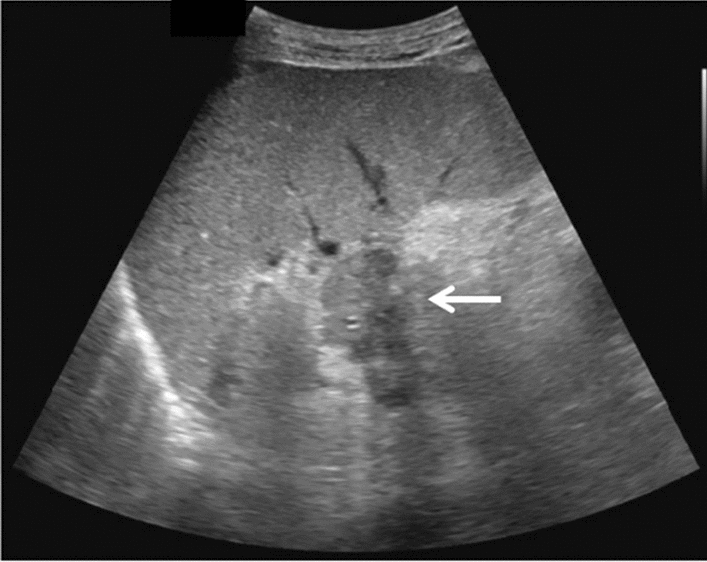
Other findings. Solid lesion in the splenic hilum. Category 3, Assessment D2

**Fig. 103:**
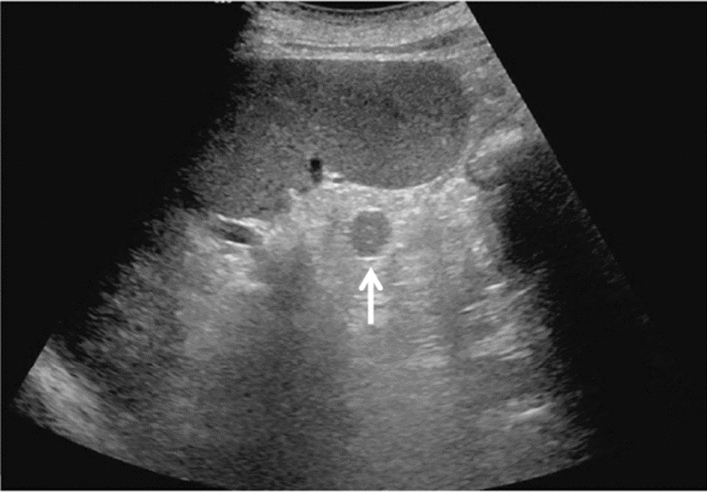
Other findings. Solid lesion in the splenic hilum (image of oval mass with homogeneous internal echo at echo level equal to that of the spleen). Category 2, Assessment B

**Table 8:** Kidneys

Kidneys				
Ultrasound imaging finding	Category	Ultrasound finding (described in Report Form)	Assessment	Figure number
Post-resection	0	Post-nephrectomy	B	
Post-partial resection/Post-renal transplantation^*1)^	2	Post-partial nephrectomy /Post-renal transplantation	B	Figures 105, 106
Unable to visualize	0	Unable to visualize kidneys	D2	
Morphological abnormality				
Maximum diameter ≥ 12 cm on both sides	3	Renal enlargement	D2	
Maximum diameter ≥ 8 cm on both sides	2	Renal atrophy	D2	
Different size between bilateral kidneys/congenital malformation, etc.^*2)^	2	Renal deformity	B	Figures 107, 108, 109
Rugged contour or splitting and deformation of central echo complex^*3)^	3	Renal mass	D2	Figures 110, 111, 112
Solid lesion^*4)^				Figure 113
A solid lesion is present	3	Renal mass	D2	Figure 114
Round lesion with distinct border and smooth contour	4	Renal tumor suspected	D2	
Any one of internal anechoic region, peripheral hypoechoic zone, or lateral shadow is present	4	Renal tumor suspected	D2	Figure 115
Splitting and deformation of central echo complex are present	4	Renal tumor suspected	D2	Figure 116
Internal anechoic region is present in round lesion with distinct border and smooth contour	5	Renal tumor	D1	
Internal anechoic region is present with either of peripheral hypoechoic zone or lateral shadow	5	Renal tumor	D1	Figure 117
Brightness equal to or higher than that of the central echo complex with irregular contour or comet image Maximum diameter < 40 mm^*5), *6)^	2	Renal angiomyolipoma	C	Figures 118, 119
Maximum diameter ≥ 40 mm	2	Renal angiomyolipoma	D2	Figure 120
Cystic lesion				
A cystic lesion is present^*7)^	2	Renal cyst	B	Figures 121, 122
Five or more cysts are present bilaterally^*8)^	2	Polycystic kidney disease	D2	Figures 123, 124
Multiple thin septi or nodular calcification are present	3	Renal cystic mass	C	Figures 125, 126
Solid component (mural nodules/wall thickening/septal thickening) and change in internal fluid (e.g., internal echogenic spots) are present^*9)^	4	Renal cystic tumor suspected	D2	Figures 127, 128
Other findings				
Calcified lesion				
In renal parenchyma^*10)^	2	Nephrocalcinosis	B	Figures 129, 130
Maximum diameter < 10 mm in pelviocaliceal system	2	Renal stone	C	Figure 131
Maximum diameter ≥ 10 mm in pelviocaliceal system	2	Renal stone	D2	Figure 132
Pyelectasis (unknown cause of occlusion)	3	Pyelectasis/hydronephrosis	D2	Figure 133
Mild pyelectasis (without caliectasis)	2	Pyelectasis	B	Figure 134
Calcified lesion in dilated region or occluded region	2	Renal pelvis stone or ureter stone^*11)^	D2	Figures 135, 136
Solid lesion in occluded region	4	Renal pelvic tumor or ureter tumor^*11)^	D2	Figure 137
Vascular abnormality^*12)^	2	Renal vascular abnormality	D2	Figure 138, 139
No abnormal findings	1	Normal kidneys	A	

^*1)^ In the case of partial resection, document the resection site if known, and evaluate the ultrasound imaging findings of the remaining portion

^*2)^ A congenital deformity (e.g., double renal pelvis, horseshoe kidney) is regarded as Category 2 and Assessment B, and parts other than the deformed part are evaluated using the same method as others

^*3)^ Irregularity/deformation of the renal contour or localized bulge into the central echo complex with echo level and speckle pattern similar to that of the renal parenchyma is regarded as a deformity and assessed as Category 2 and Assessment B. It is desirable to confirm a vascular structure similar to that of the normal renal parenchyma on color Doppler

^*4)^ Solid lesions < 10 mm can be regarded as Assessment C (because cases with a small tumor diameter show slow tumor growth, although some cases where it is difficult to differentiate from renal carcinoma are included)

^*5)^ Comet image refers to an ultrasound image where the echo of the posterior surface of a lesion becomes indistinct due to multiple reflection, making it look like an enlarged comet-like echo with deep echo attenuation

^*6)^ A renal angiomyolipoma < 40 mm showing signs or symptoms of enlargement can be regarded as Assessment D2 as there is a risk of rupture

^*7)^ Cysts with two or fewer thin septi and microcalcification are regarded as Category 2 and Assessment B

^*8)^ In cases where the longest diameter of either of the kidneys is ≤ 9 cm, the likelihood is high that it is a simple cyst rather than polycystic kidney disease, and it can be regarded as Category 2 and Assessment C (Fig. 104)

^*9)^ Changes in internal fluid (e.g., intracystic hemorrhage/infection) are also subject to thorough examination as the possibility of neoplasticity cannot be ruled out. In addition, neoplastic cysts, the generic term for cysts covered with cells showing neoplastic proliferation, are also included in this category

^*10)^ If it cannot be determined whether it is in the renal parenchyma or in the pelviocaliceal system, regard it as nephrocalcinosis or renal stone, and regard < 10 mm as Assessment C and ≥ 10 mm as Assessment D2

^*11)^ Document the occluded site if known

^*^12) Vascular abnormalities include vascular aneurysms, arterial-venous shunts (including arteriovenous malformation), venous emboli (thrombi), collateral flow and so on. However, vascular abnormalities associated with solid lesions should be evaluated according to the criteria for solid lesion (Fig. 140)

**Fig. 104:**
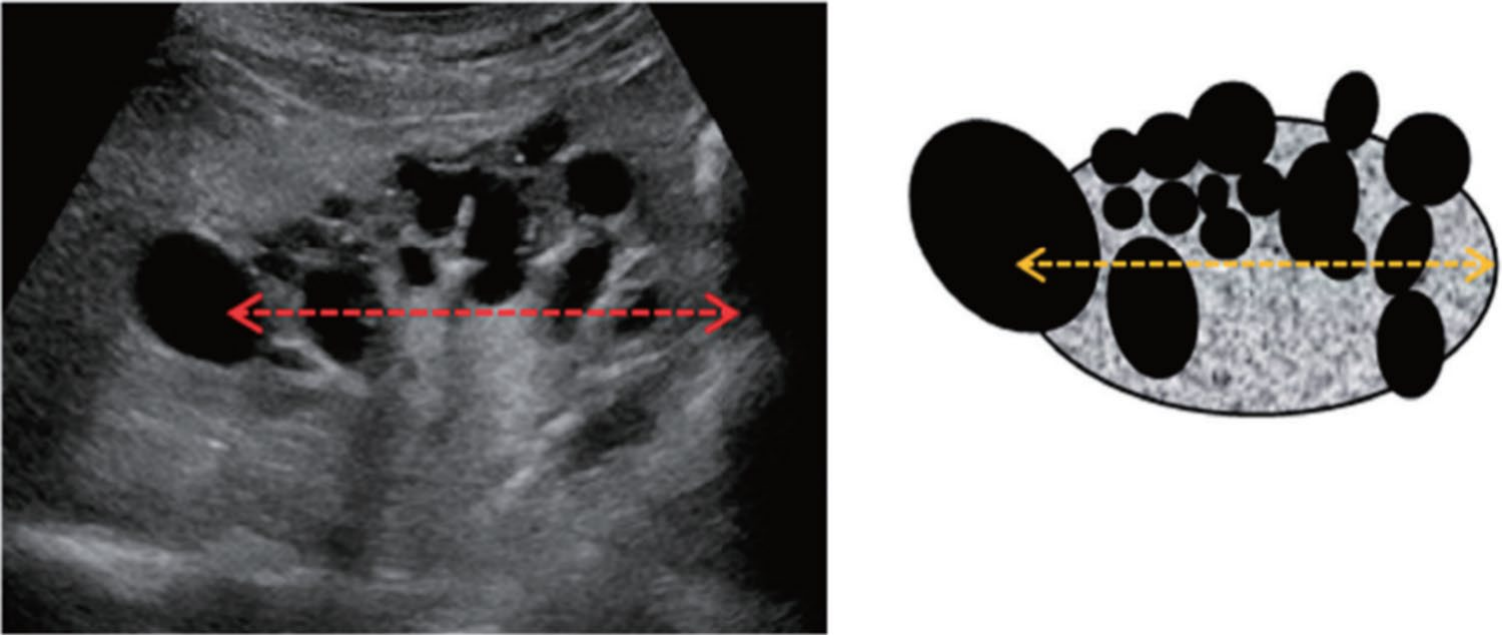
Measurement of kidney diameter. Measure the longest diameter where the renal parenchyma would normally be, without including cyst protruding outside the kidney

**Fig. 105:**
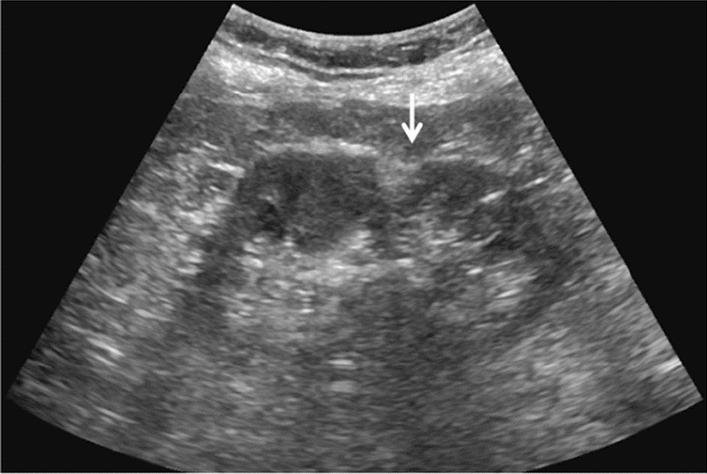
Post-partial resection/post-renal transplantation. Post-partial resection. Category 2, Assessment B

**Fig. 106:**
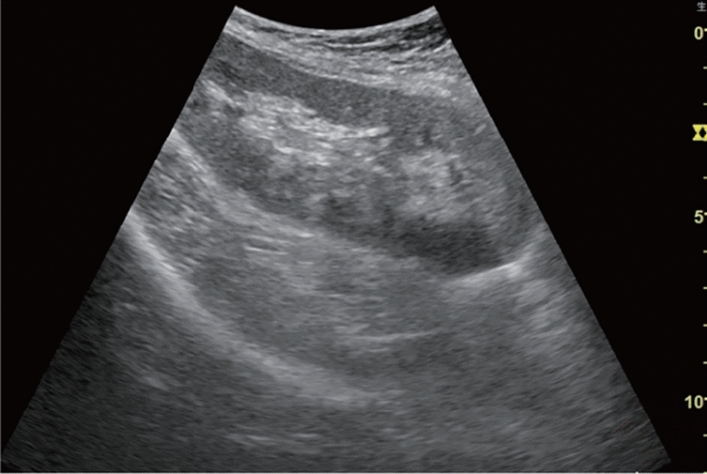
Post-partial resection/post-renal transplantation. Transplanted kidney (post-transplantation in pelvic cavity). Category 2, Assessment B

**Fig. 107:**
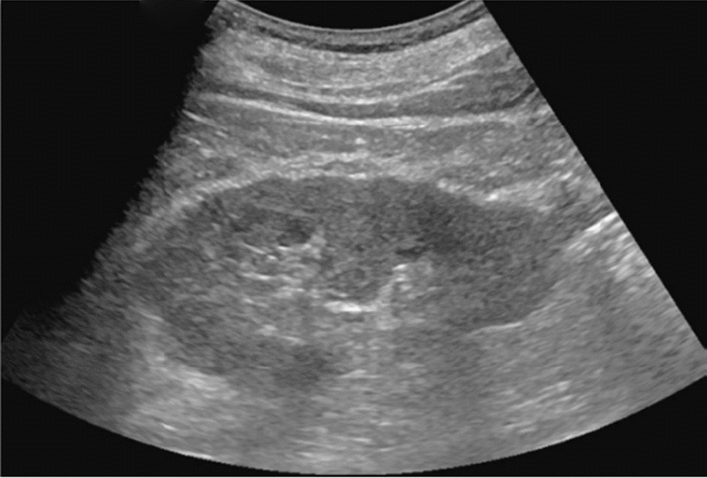
Morphological abnormality. Congenital deformity (renal column of Bertin). Category 2, Assessment B

**Fig. 108:**
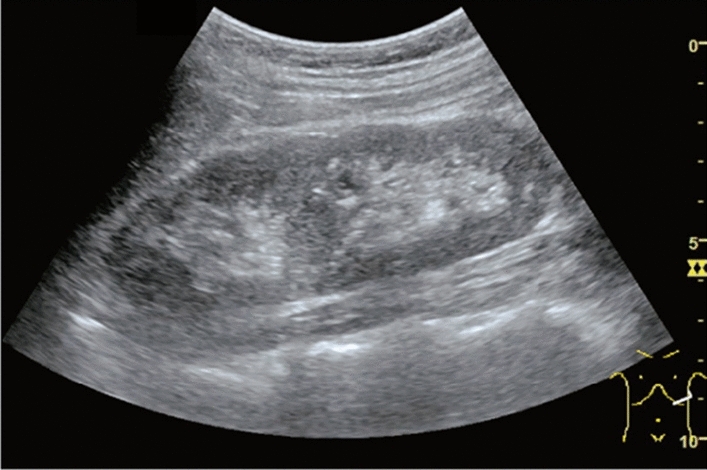
Morphological abnormality. Congenital deformity (double renal pelvis). Category 2, Assessment B

**Fig. 109:**
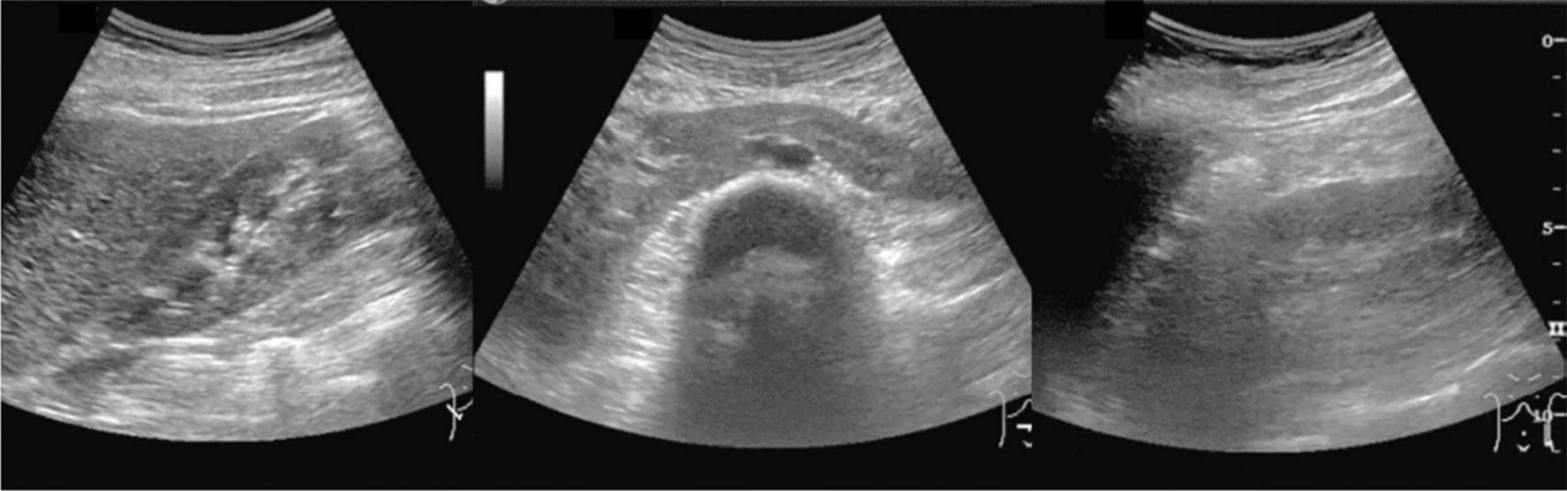
Morphological abnormality. Congenital deformity (horseshoe kidney). Right abdominal vertical scan, abdominal midline horizontal scan, and left abdominal vertical scan. Category 2, Assessment B

**Fig. 110:**
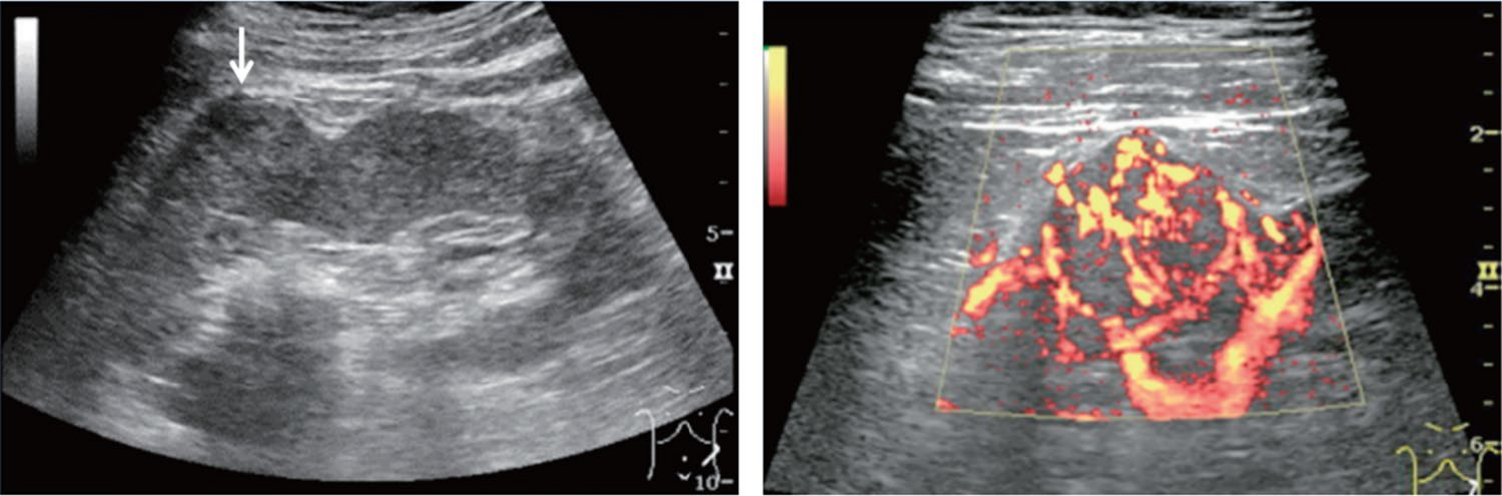
Morphological abnormality. Rugged contour is present (basket-like blood flow is displayed in lesion on color Doppler). Left: convex probe; right: power Doppler with high-frequency probe. Category 3, Assessment D2

**Fig. 111:**
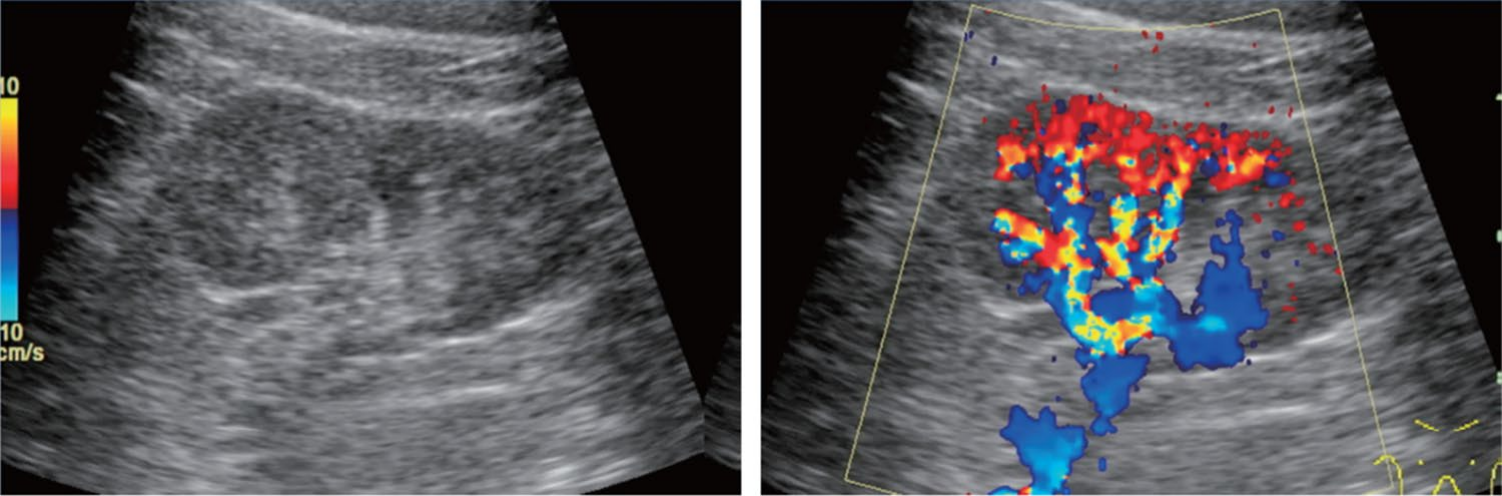
Morphological abnormality. A rugged contour is present (vascular structure similar to that of the normal renal parenchyma on color Doppler). Left: renal long-axis view; right: color Doppler. Category 2, Assessment B

**Fig. 112:**
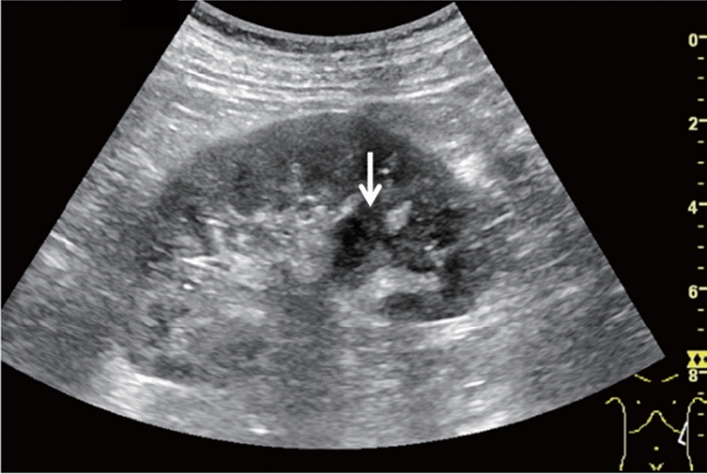
Morphological abnormality. Splitting and deformation of central echo complex. Category 3, Assessment D2

**Fig. 113:**
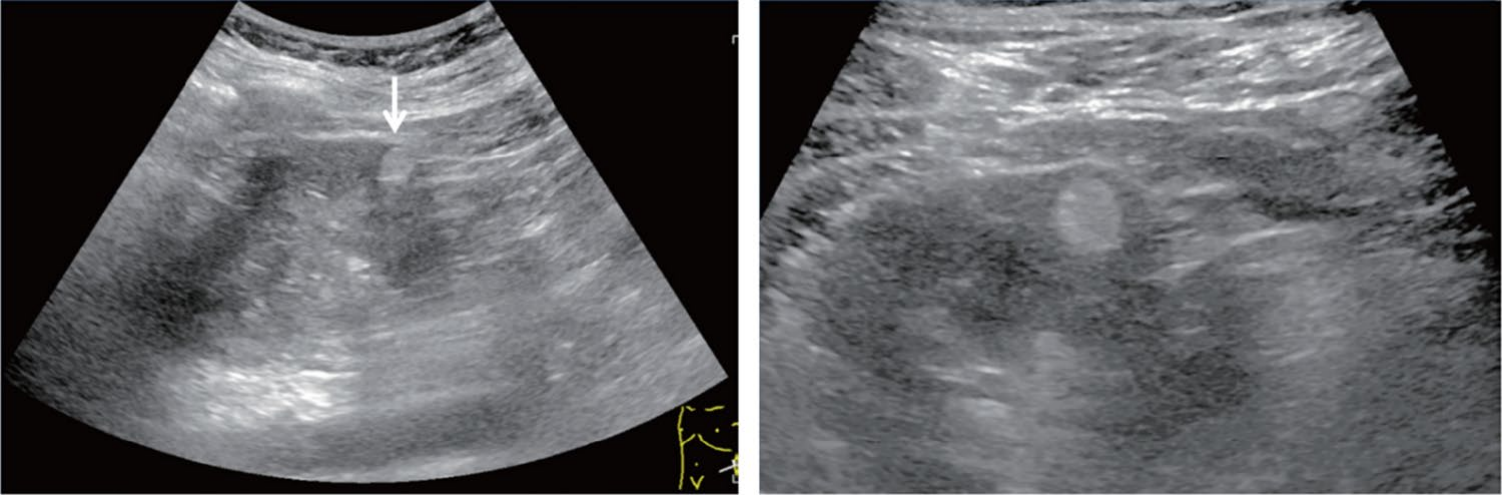
Solid lesion. Hyperechoic mass with maximum diameter < 10 mm. Left: convex probe; right: high-frequency linear probe. Category 3, Assessment C

**Fig. 114:**
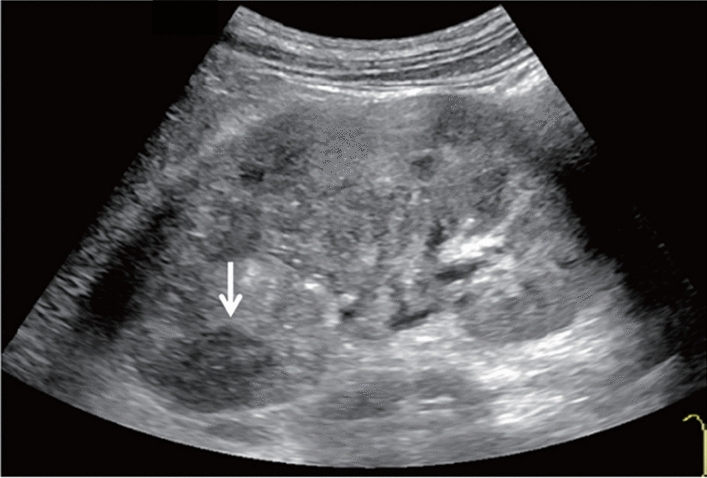
Solid lesion. A solid lesion is present (hypoechoic mass with irregular contour). Category 3, Assessment D2

**Fig. 115:**
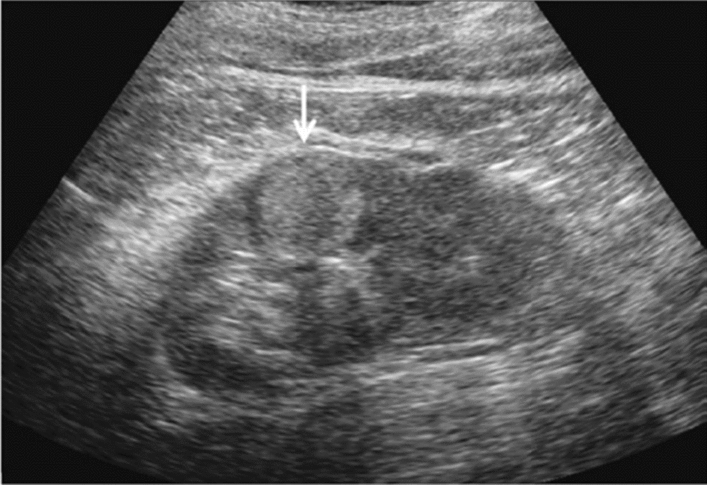
Solid lesion. A peripheral hypoechoic zone is present in a round lesion with a distinct border and smooth contour. Category 4, Assessment D2

**Fig. 116:**
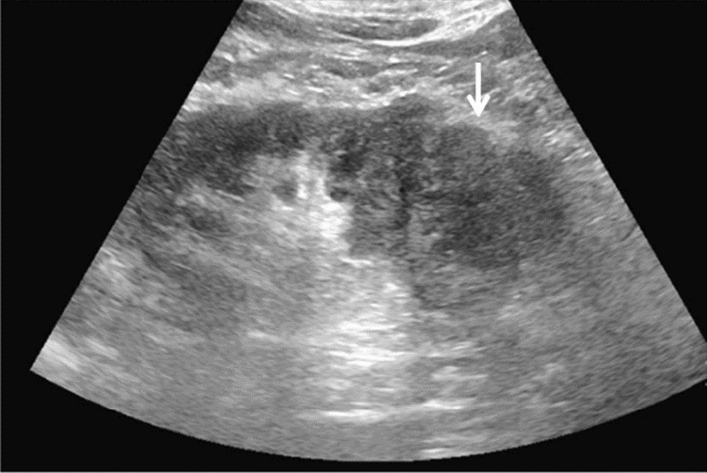
Solid lesion. Deformation of the central echo complex is present (hypoechoic lesion with irregular contour). Category 4, Assessment D2

**Fig. 117:**
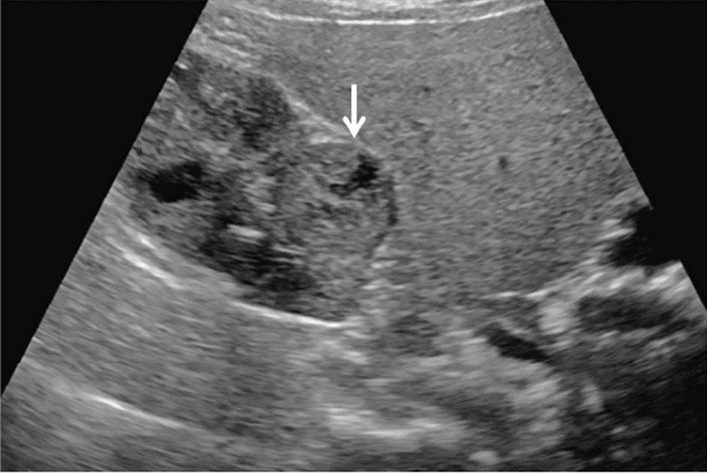
Solid lesion. An internal anechoic region with a peripheral hypoechoic zone and lateral shadow is present in a round lesion with a distinct border and a smooth contour. Category 5, Assessment D1

**Fig. 118:**
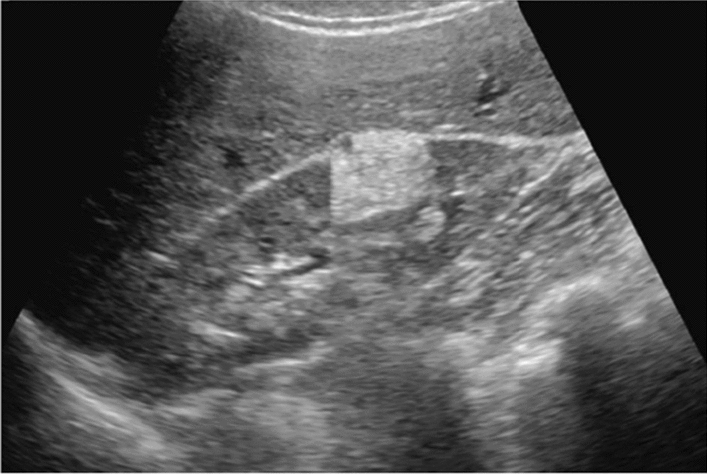
Solid lesion. Brightness equal to or higher than that of the central echo complex with an irregular contour. Maximum diameter < 40 mm. Category 2, Assessment C

**Fig. 119:**
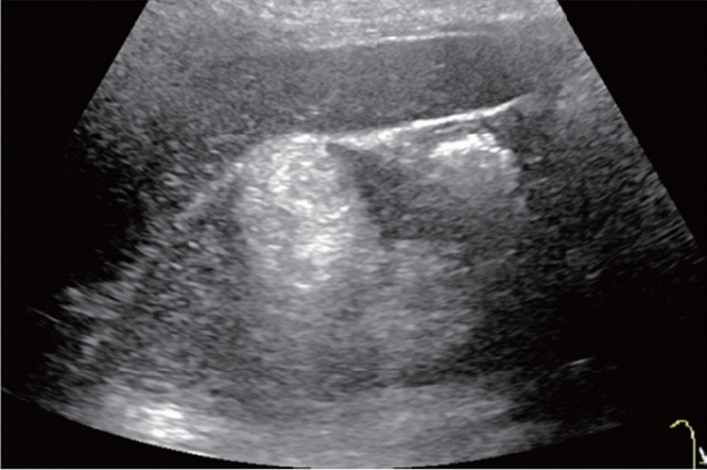
Solid lesion. Brightness equal to or higher than that of the central echo complex a comet image is present. Maximum diameter < 40 mm. Category 2, Assessment C

**Fig. 120:**
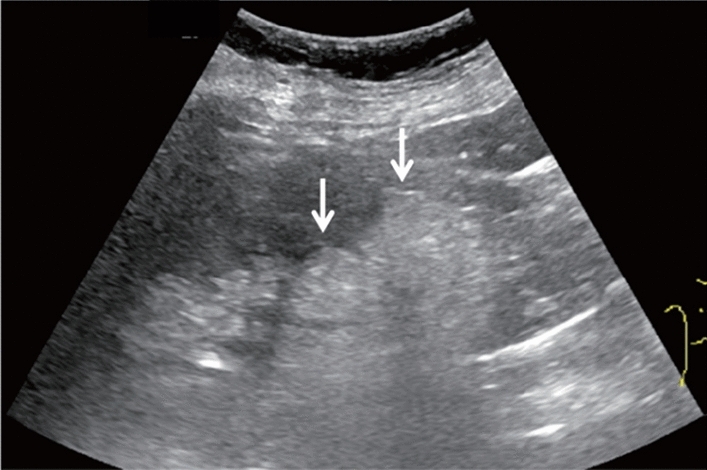
Solid lesion. Brightness equal to or higher than that of the central echo complex with an irregular contour and comet image is present. Maximum diameter ≥ 40 mm. Category 2, Assessment D2

**Fig. 121:**
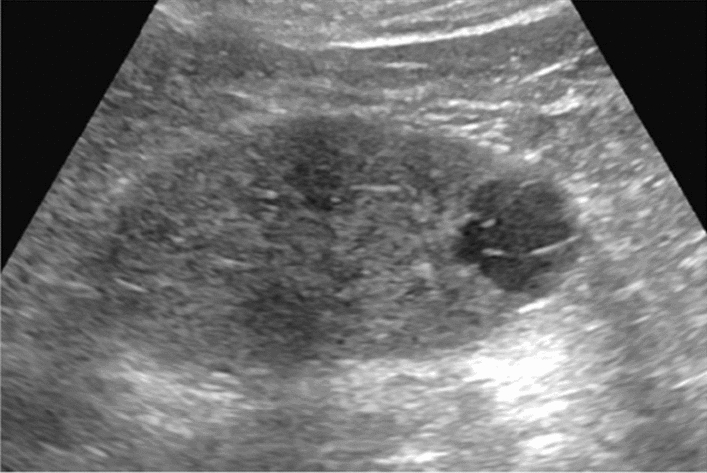
Cystic lesion. No more than two thin septi are present. Category 2, Assessment B

**Fig. 122:**
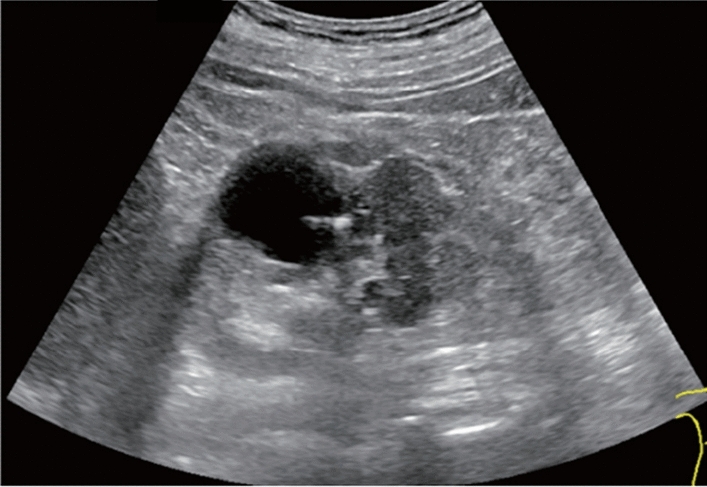
Cystic lesion. Microcalcification is present. Category 2, Assessment B

**Fig. 123:**
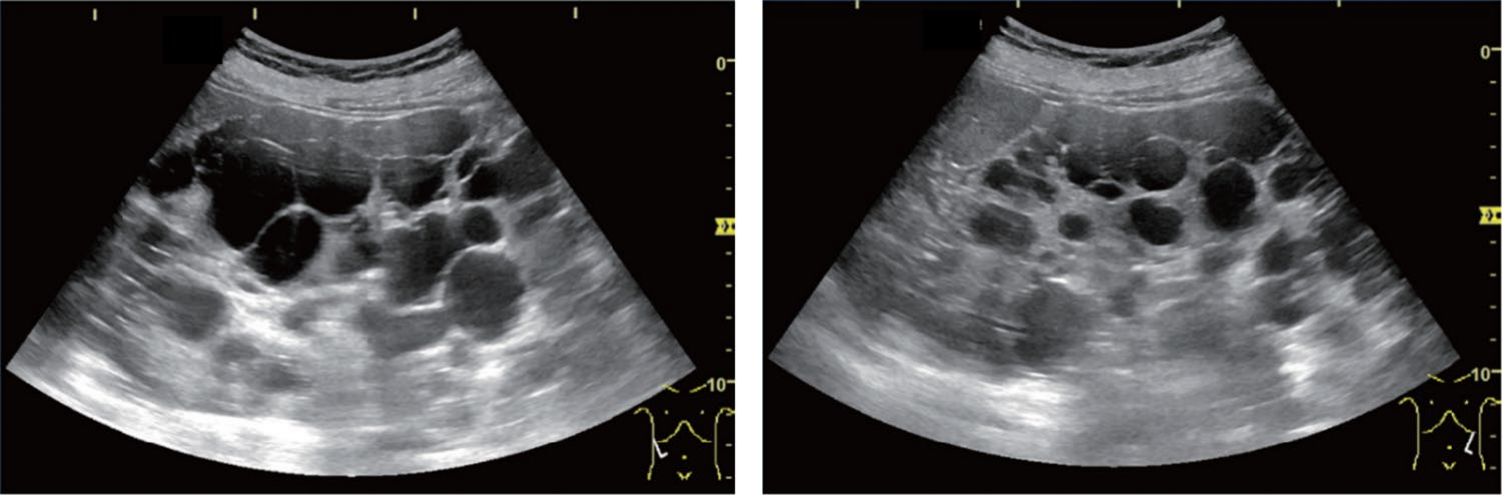
Cystic lesion. Five or more cysts are present bilaterally (longest diameter > 9 cm on both sides). Left: right renal long-axis view; right: left renal long-axis view. Category 2, Assessment D2

**Fig. 124:**
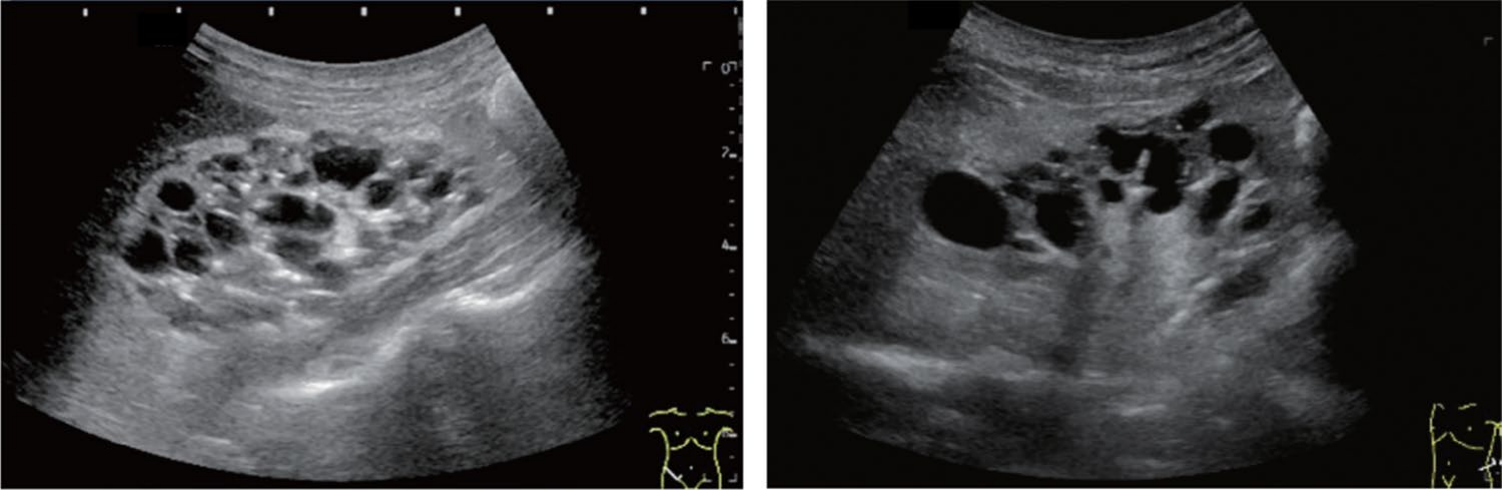
Cystic lesion. Five or more cysts are present bilaterally (longest diameter ≤ 9 cm). Left: right renal long-axis view, right: left renal long-axis view. Category 2, Assessment C

**Fig. 125:**
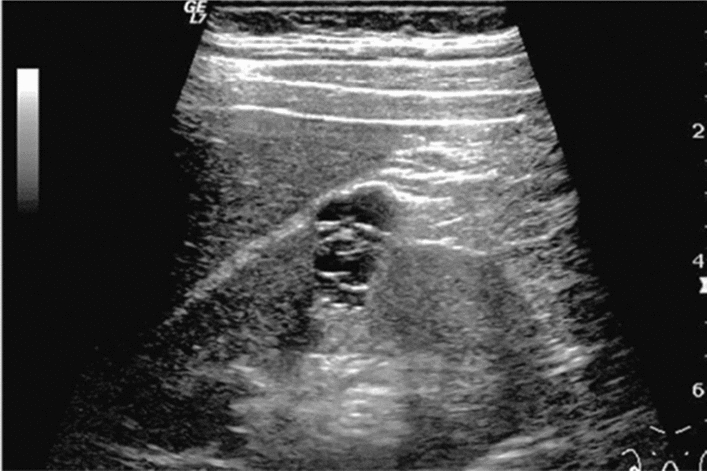
Cystic lesion. Multiple thin septi are present. Category 3, Assessment C

**Fig. 126:**
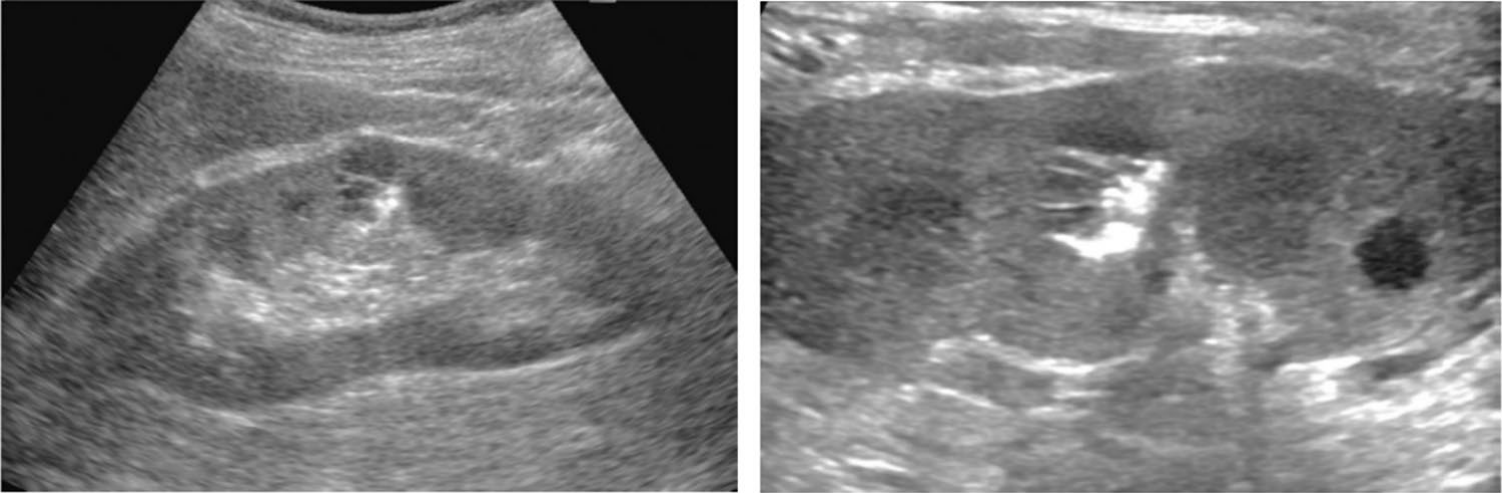
Cystic lesion. Multiple thin septi and nodular calcification are present. Left: convex probe; right: high-frequency probe. Category 3, Assessment C

**Fig. 127:**
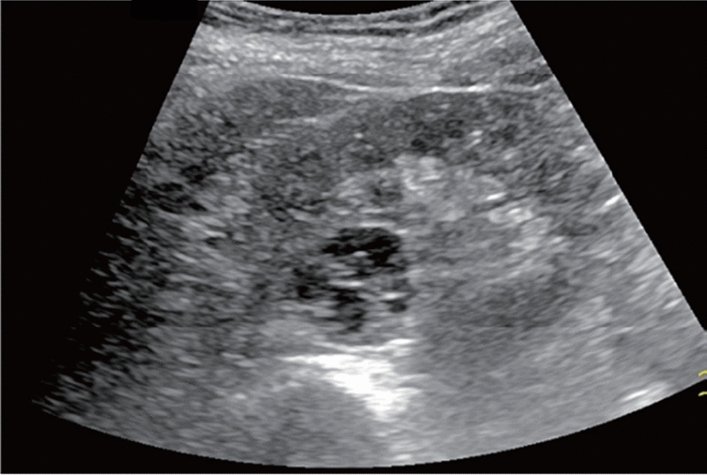
Cystic lesion. A solid component (septal thickening) is present. Category 4, Assessment D2

**Fig. 128:**
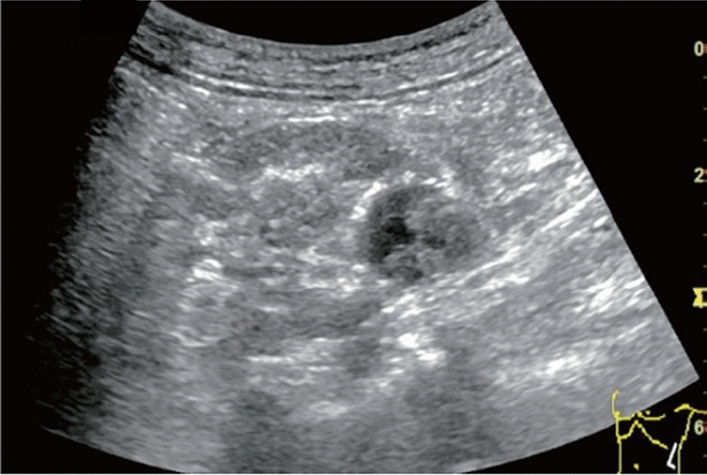
Cystic lesion. A solid component (intracystic nodules) is present. Category 4, Assessment D2

**Fig. 129:**
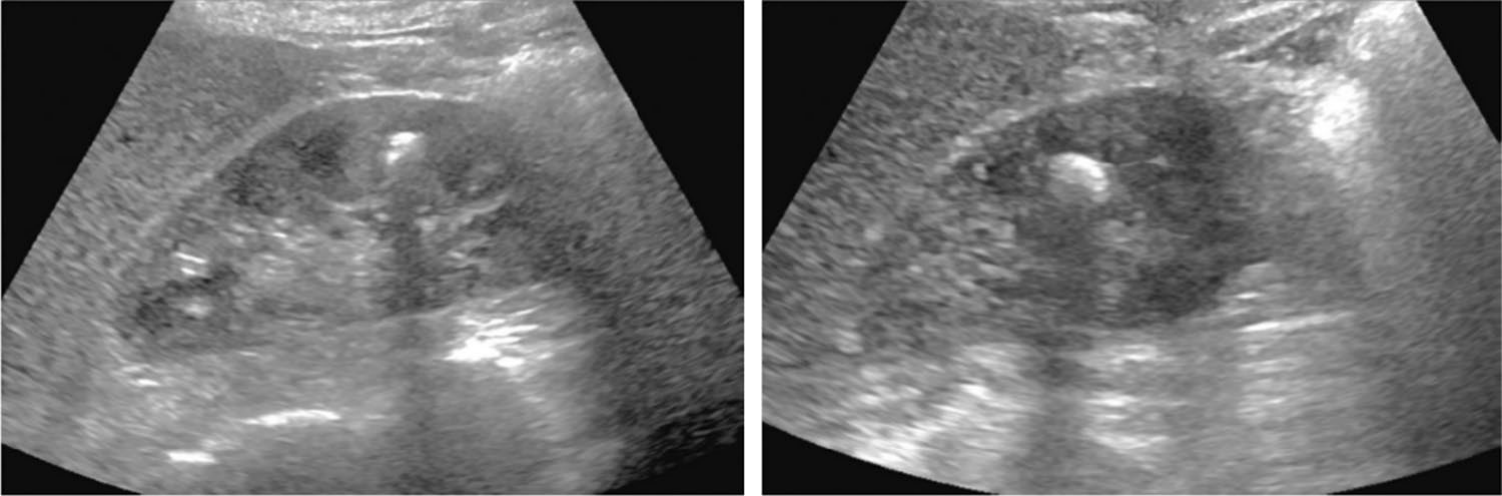
Other findings. Calcified lesion (in renal parenchyma). Category 2, Assessment B

**Fig. 130:**
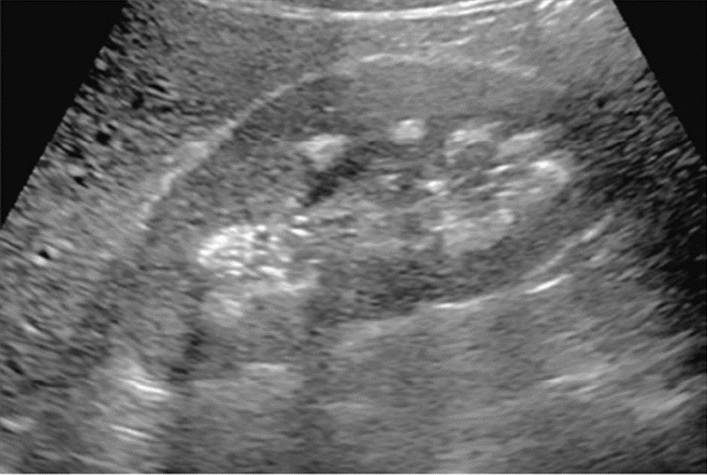
Other findings. Calcified lesion (in renal parenchyma/in pelviocaliceal system). Renal stone. Maximum diameter ≥ 10 mm. Category 2, Assessment D2

**Fig. 131:**
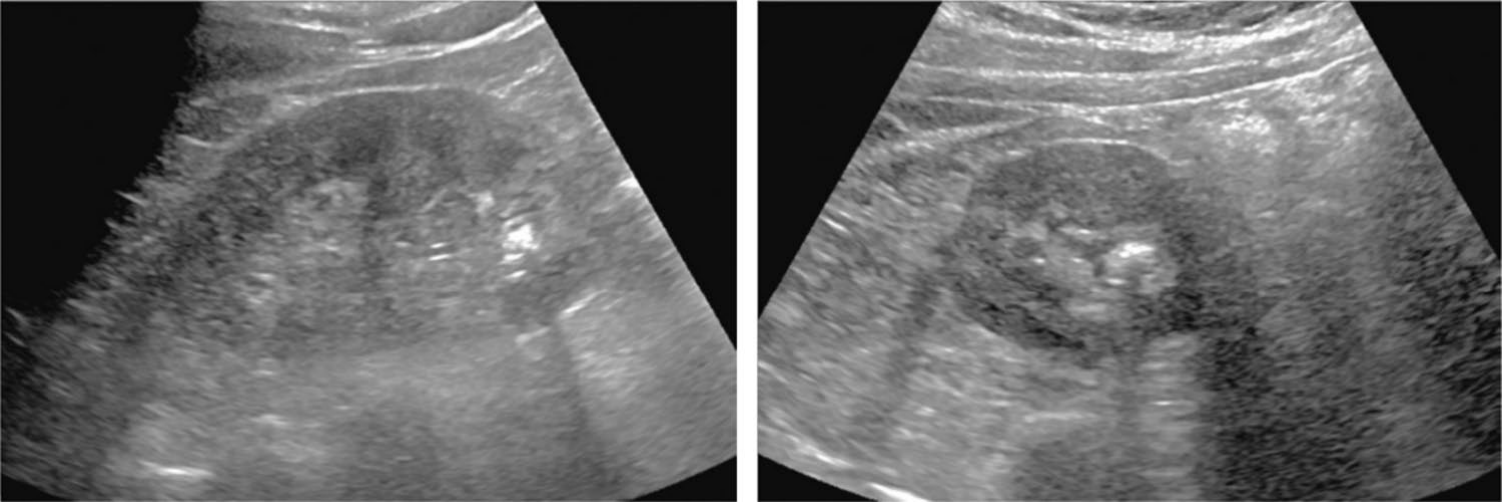
Other findings. Calcified lesion. In the pelviocaliceal system. Maximum diameter < 10 mm. Left: long-axis view; right: short-axis view. Category 2, Assessment C

**Fig. 132:**
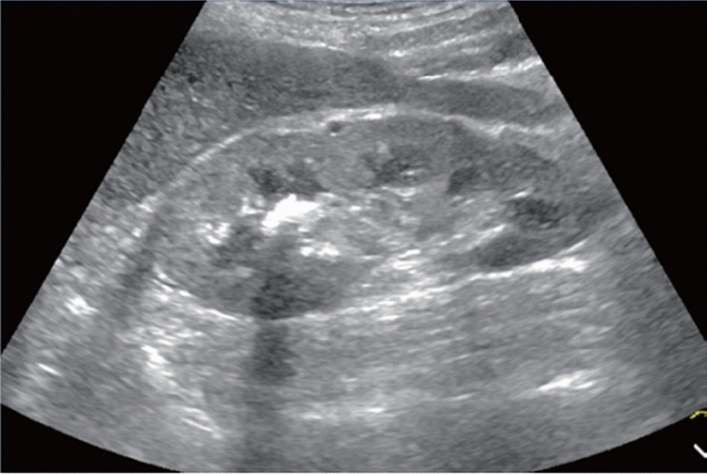
Other findings. Calcified lesion. In the pelviocaliceal system. Maximum diameter ≥ 10 mm. Category 2, Assessment D2

**Fig. 133:**
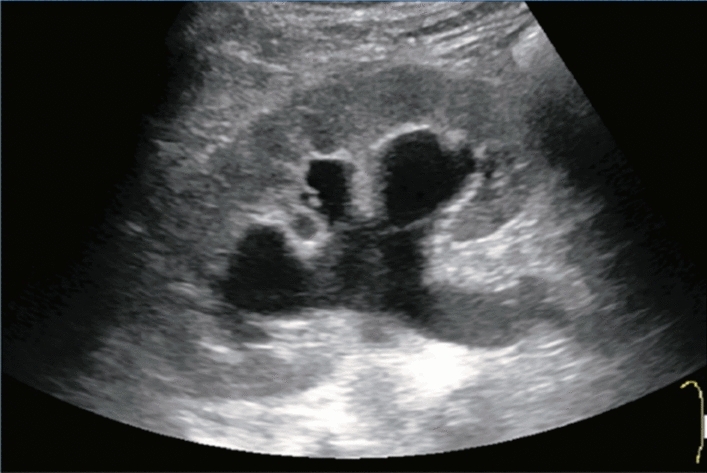
Other findings. Pyelectasis (unknown cause of occlusion). Category 3, Assessment D2

**Fig. 134:**
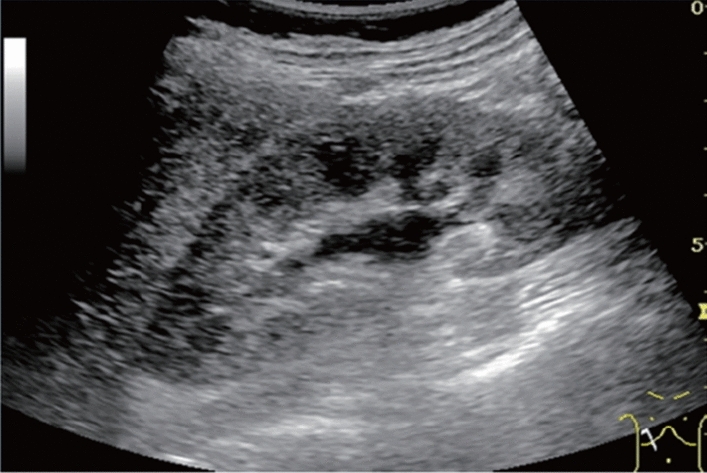
Other findings. Mild pyelectasis (without caliectasis). Category 2, Assessment B

**Fig. 135:**
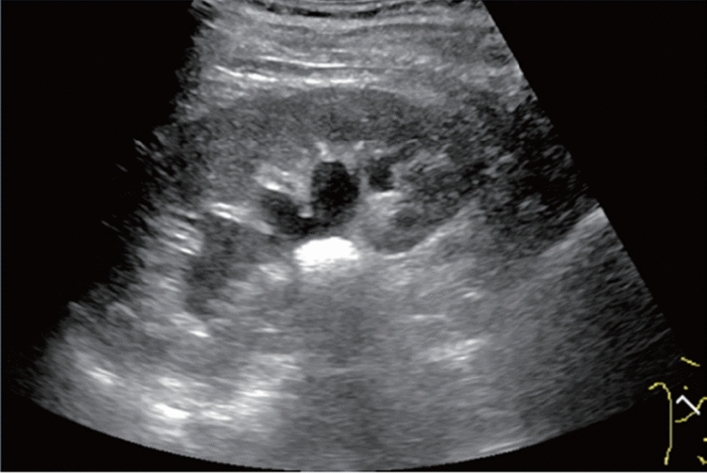
Other findings. Pyelectasis (with calcified lesion in the occluded region). Category 2, Assessment D2

**Fig. 136:**
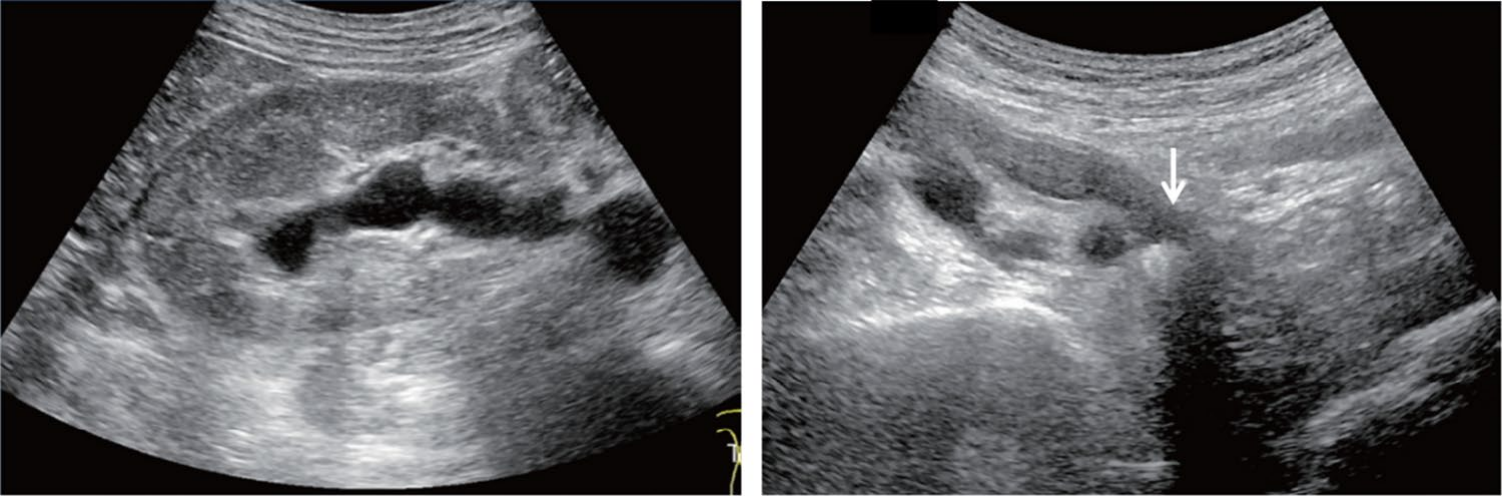
Other findings. Pyelectasis (with calcified lesion in the ureter). Category 2, Assessment D2

**Fig. 137:**
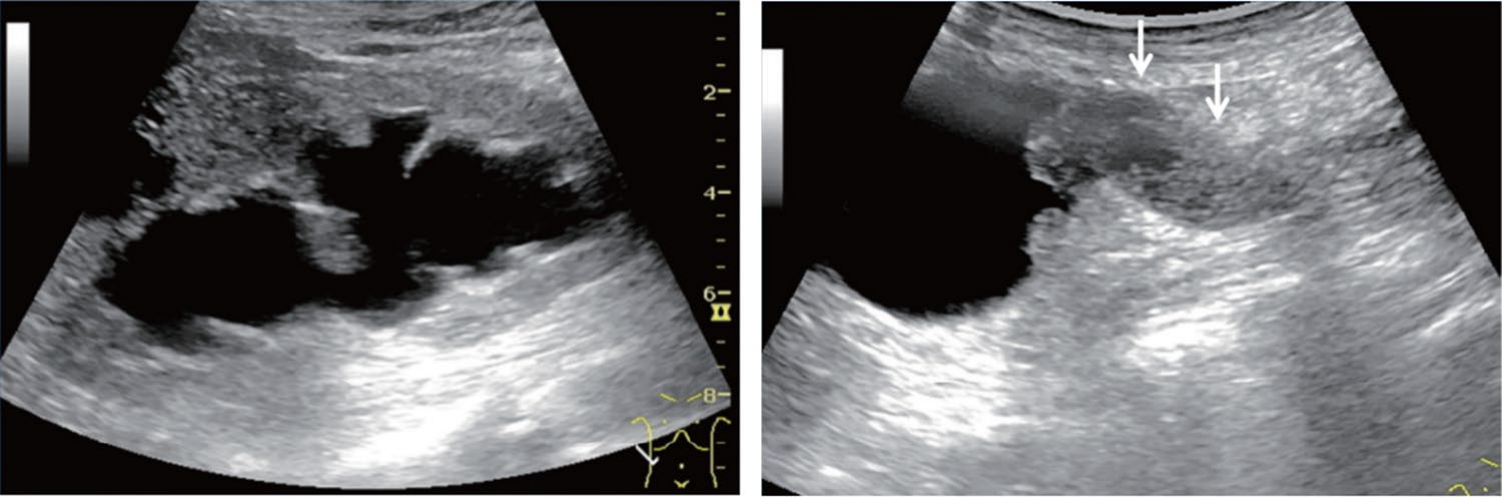
Other findings. Pyelectasis (with a solid lesion in the occluded region). Left: renal long-axis view; right: long-axis view of upper ureter from renal pelvis. Category 4, Assessment D2

**Fig. 138:**
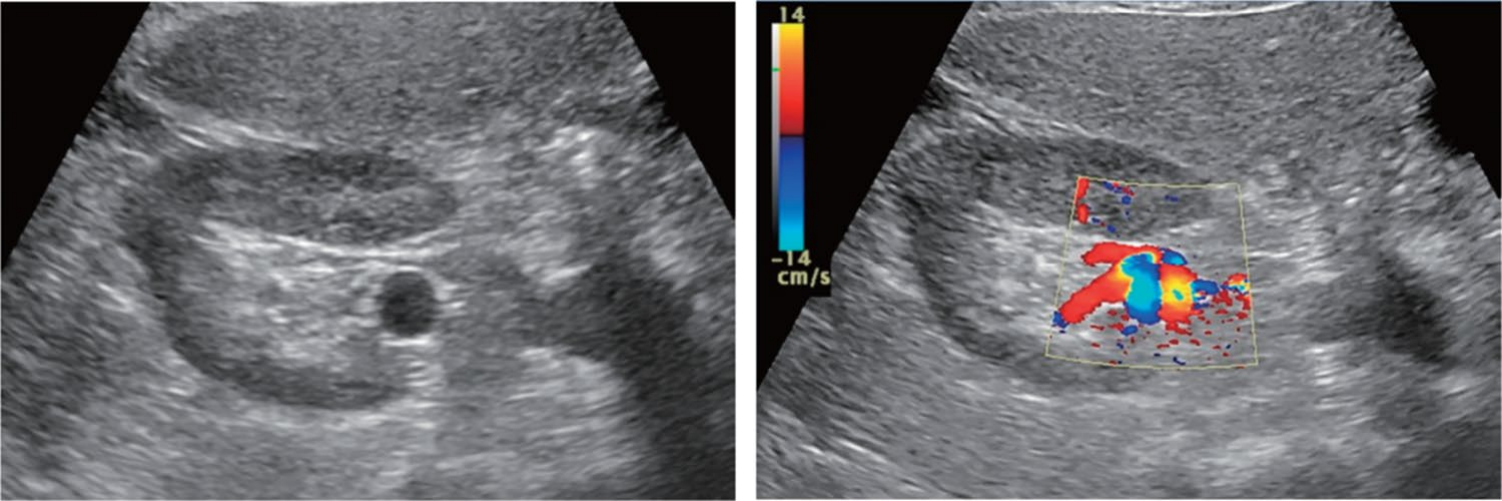
Other findings. Vascular abnormality. A cystic lesion is present in the renal hilus (left), and a blood flow signal is present on color Doppler (right). Left: renal short-axis view b-mode; right: color Doppler. Category 2, Assessment D2

**Fig. 139:**
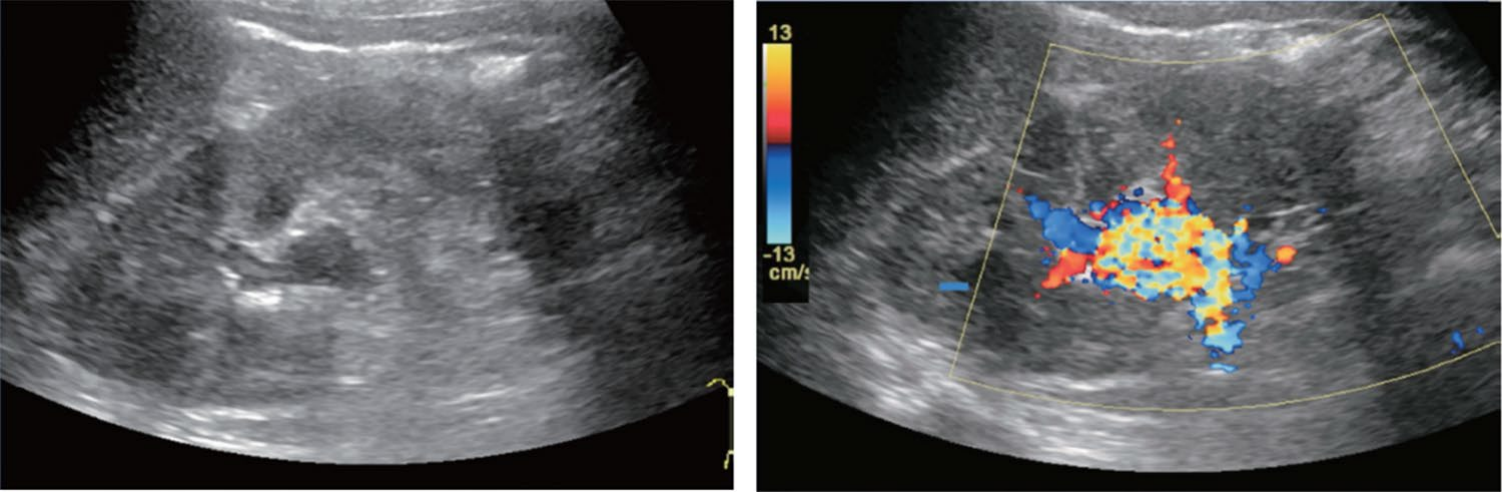
Other findings. Vascular abnormality. An irregular anechoic lesion is present in the renal central echo complex (left), and a blood flow signal is present on color Doppler (right). Left: renal long-axis view b-mode; right: color Doppler. Category 2, Assessment D2

**Table 9:** Abdominal aorta

Abdominal aorta				
Ultrasound imaging finding	Category	Ultrasound finding (described in report form)	Assessment	Figure number
Post-treatment^*1)^	2	Post-abdominal aorta treatment	B	Figure 141
Unable to visualize	0	Unable to visualize abdominal aorta	B	
Localized aortic dilatation^*2)^				
Fusiform dilatation				
Maximum short diameter ≥ 30 mm, < 45 mm	2	Abdominal aortic aneurysm	C	Figure 142
Maximum short diameter ≥ 45 mm, < 55 mm	2	Abdominal aortic aneurysm	D2	
Maximum short diameter ≥ 55 mm^*3)^	2	Abdominal aortic aneurysm	D1P	Figure 143
Saccular dilatation	2	Abdominal aortic aneurysm	D2P	Figure 144
Other findings				
A flap is present^*4)^	2	Abdominal aortic dissection	D2	Figure 145
Vessel wall/lumen abnormality such as plaque^*5)^	2	Arteriosclerosis	C	Figures 146, 147
No abnormal findings	1	Normal aorta	A	

^*^Panic finding: Add "P" to the assessment in the case of an urgent condition

^*1)^ Examinees who have undergone stent-graft insertion for aortic aneurysm are assessed as D2P* if the maximum aneurysm diameter has increased since the last examination (including before treatment)

^*2)^ Measure the aorta diameter as shown in Fig. 140 (according to The Japan Society of Ultrasonics in Medicine Terminology and Diagnostic Criteria Committee: Standard Evaluation Methods for Aortic Lesions Using Ultrasound 2020)

^*3)^ For saccular dilatation or fusiform dilatation ≥ 55 mm in maximum diameter, report it to the physician in charge of assessment as "P" as the risk of rupture is high

^*4)^ As a rule, the assessment of aortic dissection is D2, but fusiform aortic aneurysm applies depending on the extent of the dilatation. Assess as D2P if it is a new finding

^*5)^ If particularly large plaque or mobile plaque is found in the aorta, it can be documented. Wall thickening, calcification, or other findings can also be documented separately

**Fig. 140:**
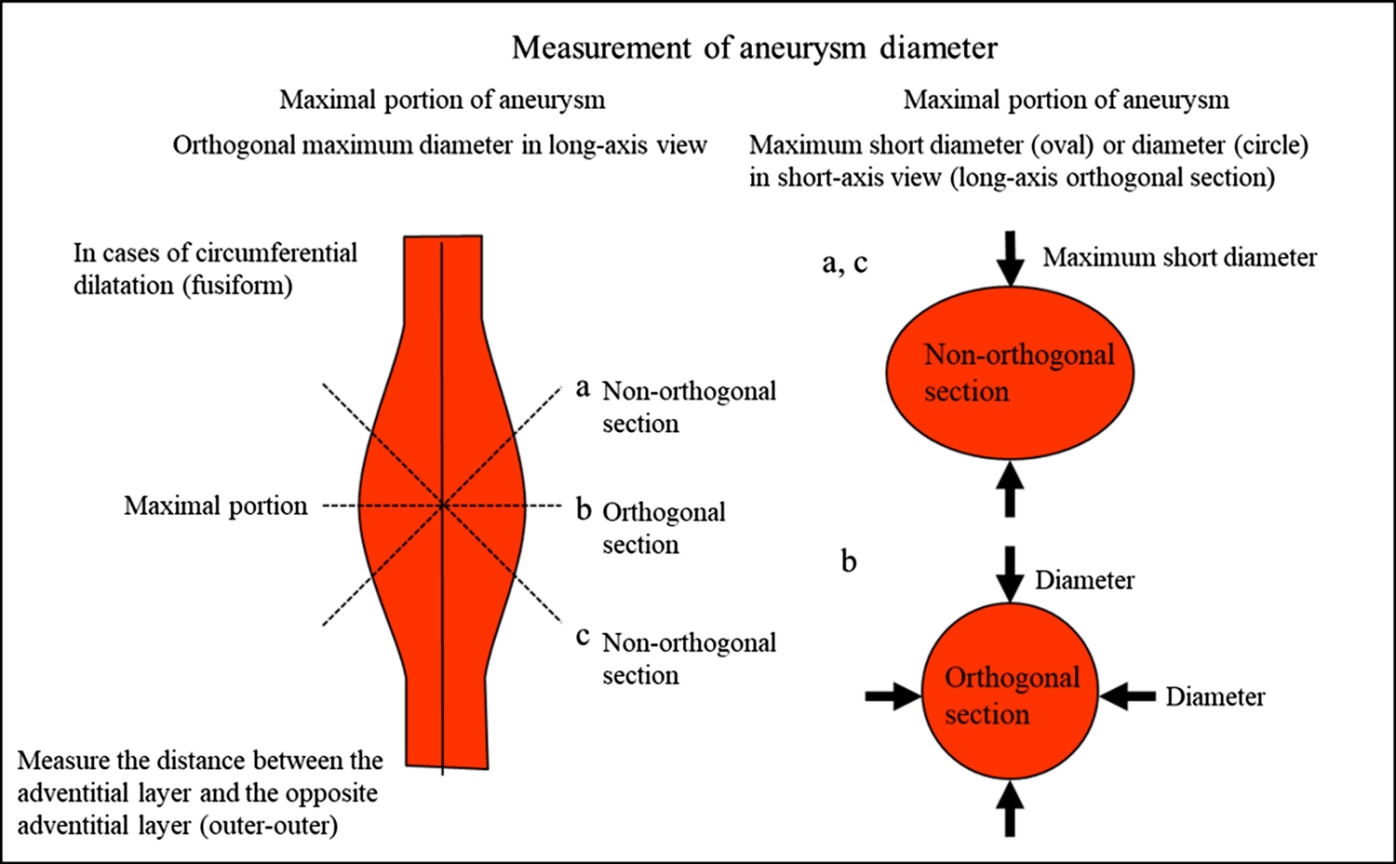
Measurement of fusiform aneurysm diameter. (https://www.jsum.or.jp/committee/diagnostic/pdf/aorticlesion2020.pdf)

**Fig. 141:**
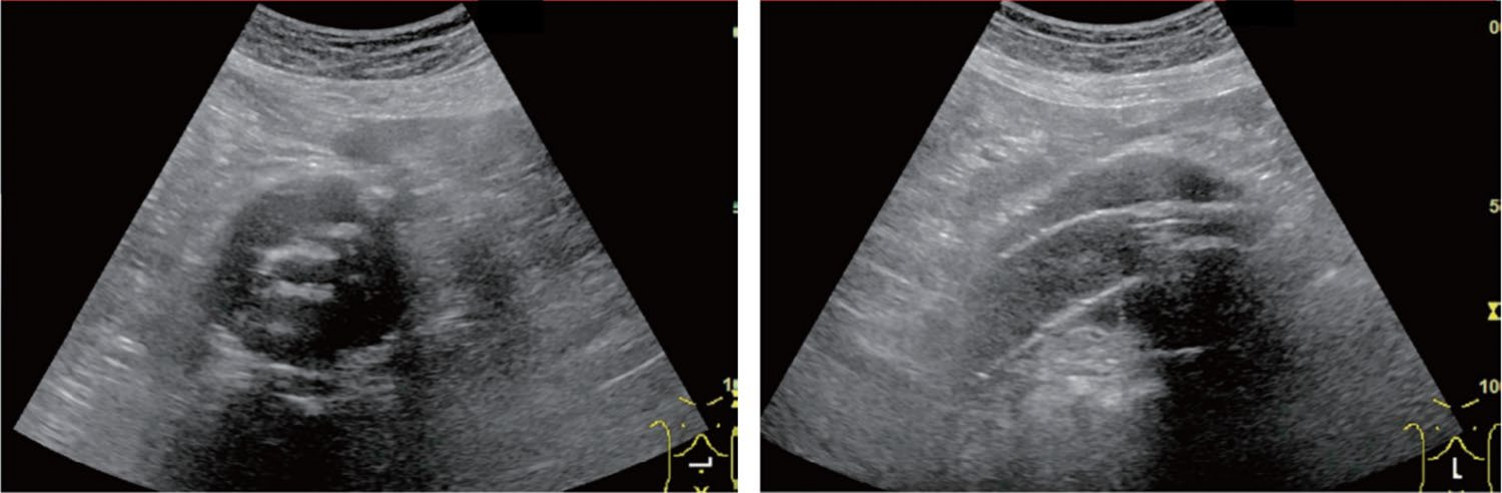
Post-treatment (stent-graft insertion). Category 2, Assessment B

**Fig. 142:**
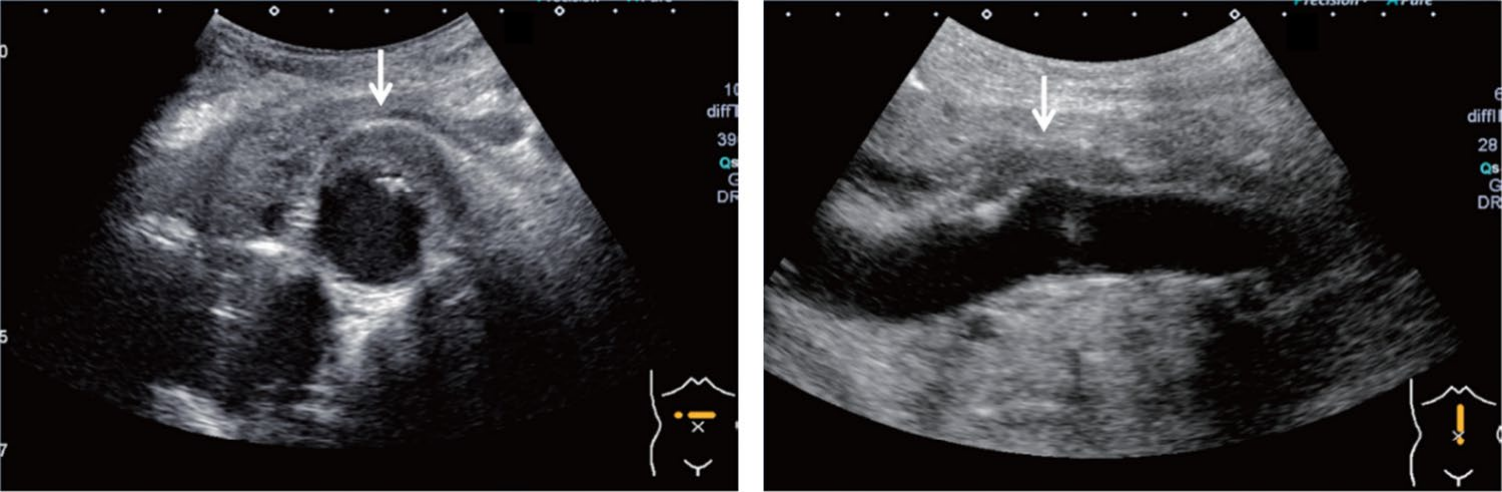
Localized aortic dilatation (32 mm). Fusiform dilatation. Maximum short diameter ≥ 30 mm, < 45 mm. Left: short-axis view; right: long-axis view. Category 2, Assessment C

**Fig. 143:**
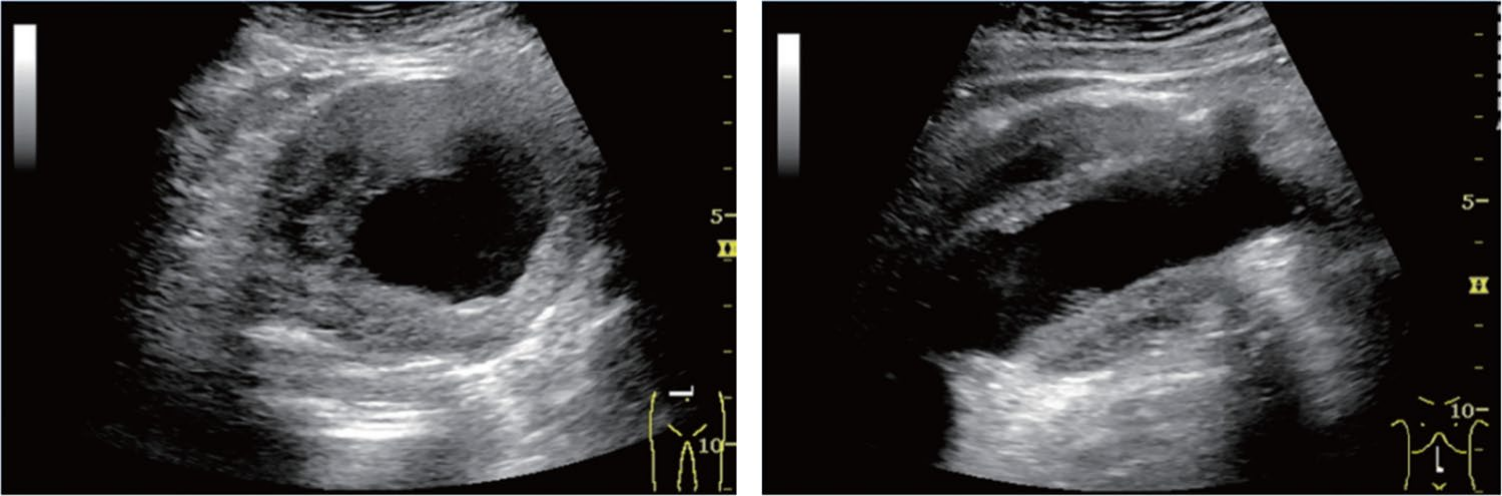
Localized aortic dilatation (62 mm). Fusiform dilatation. Maximum short diameter ≥ 55 mm. Left: Short-axis view; right: long-axis view. Category 2, Assessment D1P

**Fig. 144:**
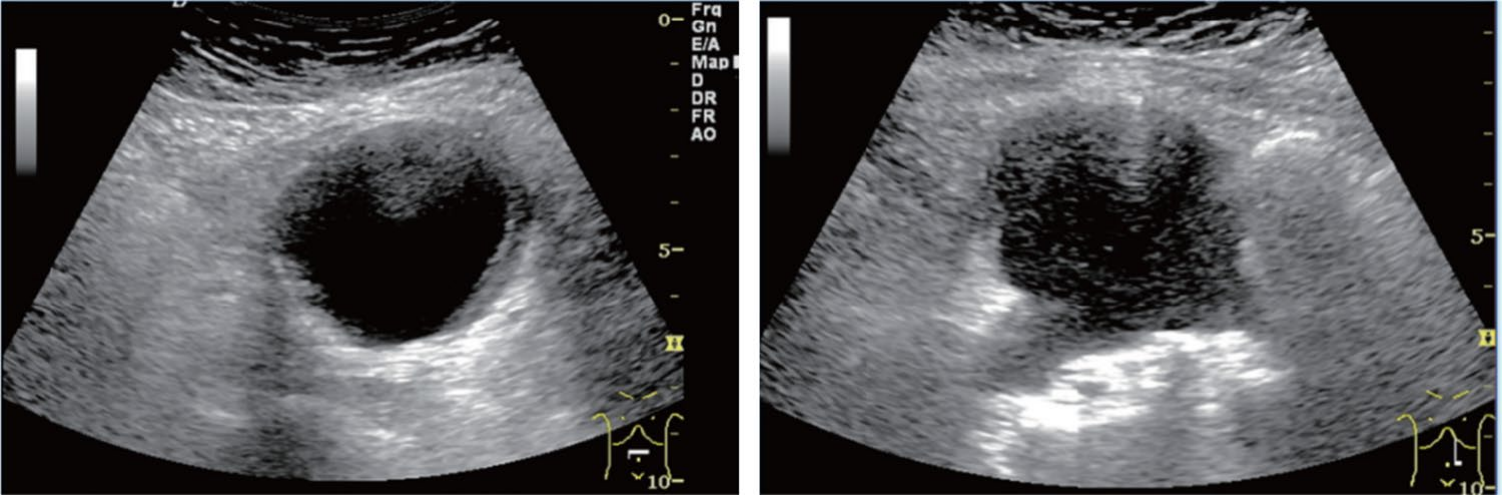
Localized aortic dilatation. Saccular dilatation (saccular aneurysm). Left: short-axis view; right: long-axis view. Category 2, Assessment D2P

**Fig. 145:**
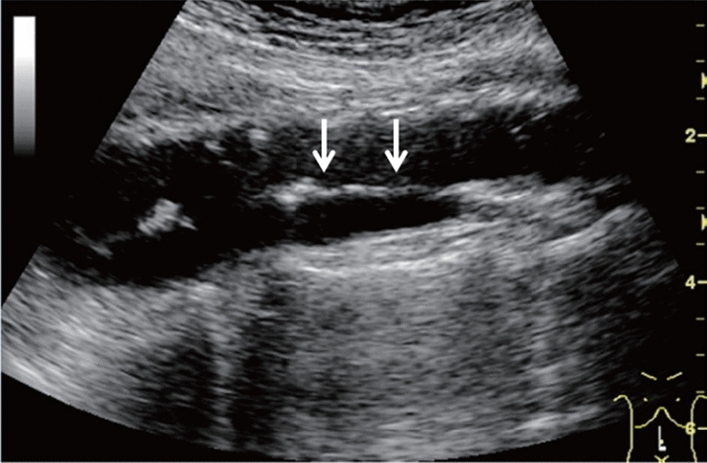
Other findings. A flap is present (maximum short diameter 23 mm). Category 2, Assessment D2

**Fig. 146:**
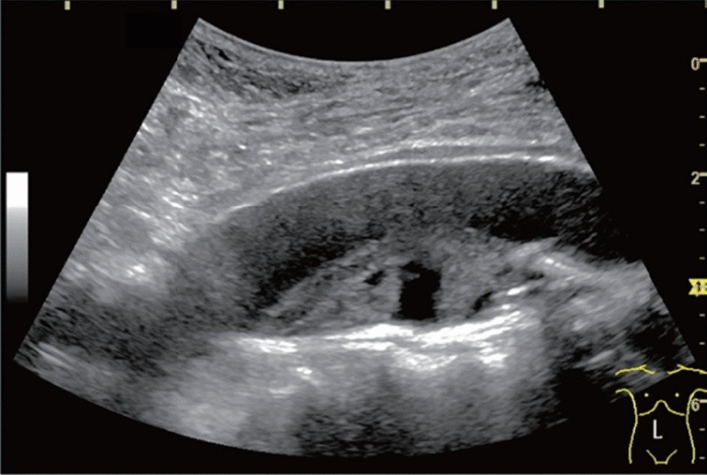
Other findings. Plaque. Category 2, Assessment C

**Fig. 147:**
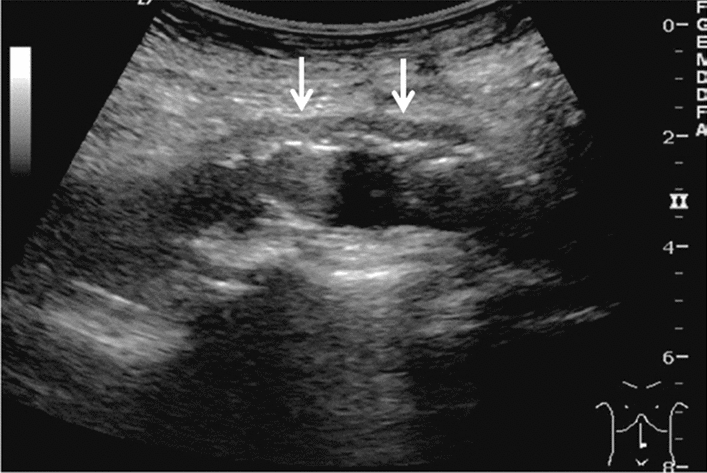
Other findings. Wall thickening and calcification. Category 2, Assessment C

**Table 10:** Others

Others				
Ultrasound imaging finding	Category	Ultrasound finding (described in Report Form)	Assessment	Figure number
Lymph-node swelling				
Short diameter ≥ 7 mm^*1)^	3	Lymph-node swelling	C	Figure 148
Either short diameter ≥ 10 mm or short/long diameter ratio ≥ 0.5	4	Lymph-node swelling	D2	Figure 149
Intraperitoneal fluid retention				
Fluid retention is present^*2)^	3	Ascites	D2	Figure 150, 151
Intrathoracic fluid retention				
Fluid retention is present^*2)^	3	Pleural effusion	D2	Figure 152, 153, 154
Fluid retention in cardiac cavity Fluid retention is present^*3)^	2	Pericardial effusion	D2	Figure 155
Abdominal cavity, retroperitoneum, or pelvic cavity (including adrenal gland)				
Mass image is present^*4)^	3	Abdominal mass	D2	Figure 156, 157

^*1)^ Record as "finding present" for lymph-node swelling with minor axis ≥ 7 mm

^*2)^ A state in which fluid retention has exceeded the physiological limit. In cases where a fluid echogenic spot (debris echo) or solid echo image in the fluid is present, it may be regarded as Category 4 in light of suspected hemorrhage/malignant disease (including peritoneal metastasis)

^*3)^ Pericardial effusion is assessed as D2 even if it is benign as it may require treatment

^*4)^ Cystic masses are included in abdominal masses

**Fig. 148:**
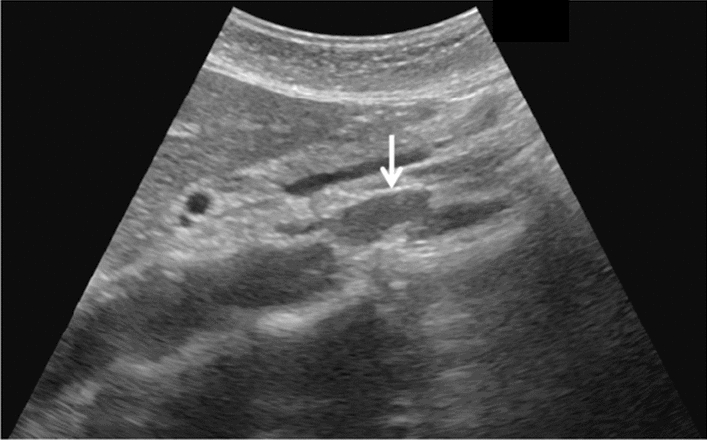
Lymph-node swelling. Minor axis ≥ 7 mm. Category 3, Assessment C

**Fig. 149:**
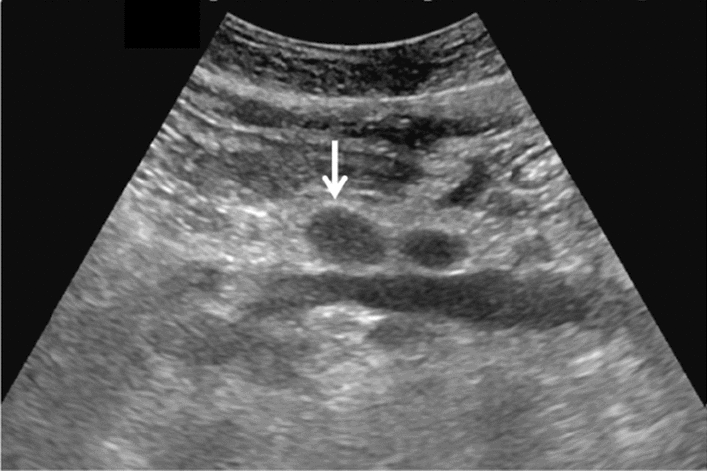
Lymph-node swelling. Either minor axis ≥ 10 mm or minor/major axis ratio ≥ 0.5. Category 4, Assessment D2

**Fig. 150:**
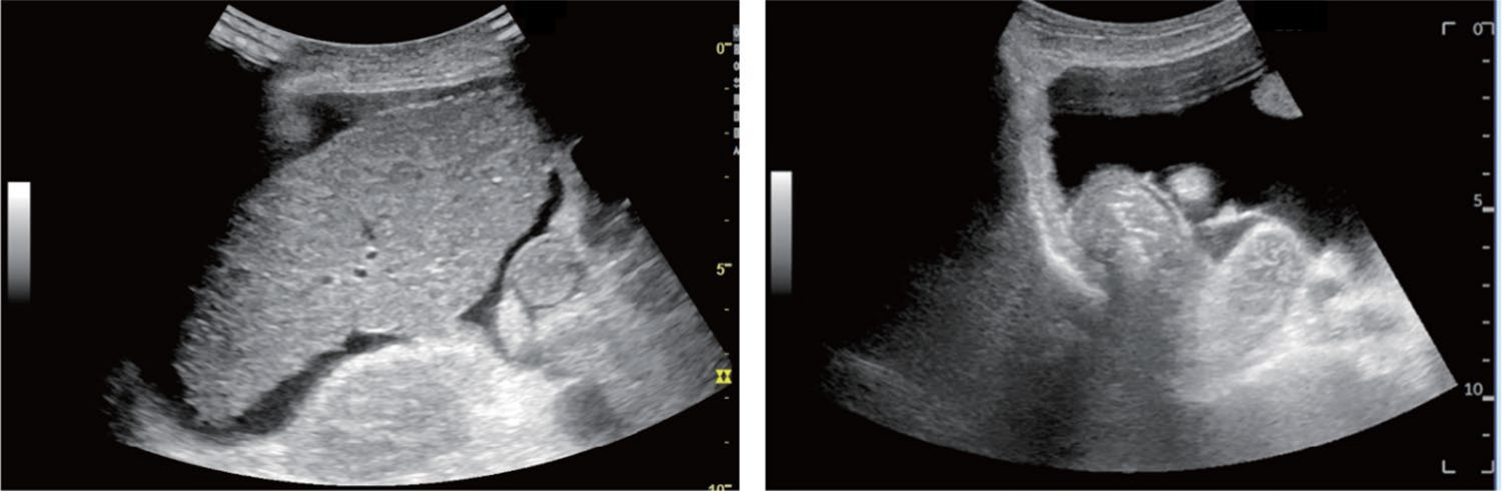
Intraperitoneal fluid retention. Left: right upper abdominal horizontal scan; right: right lower abdominal horizontal scan. Category 3, Assessment D2

**Fig. 151:**
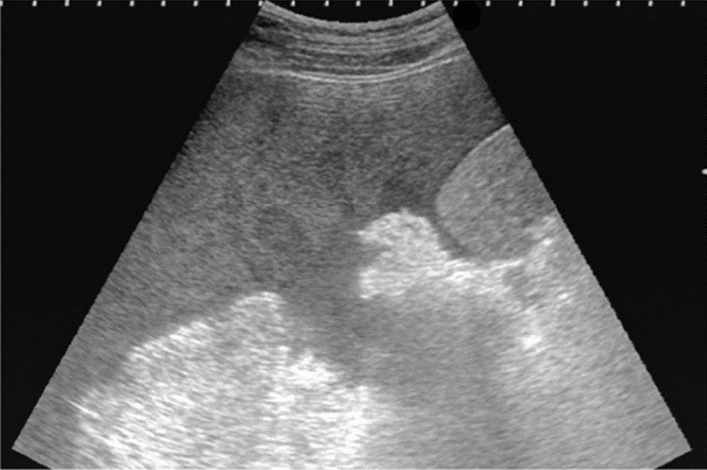
Intraperitoneal fluid retention (with debris echo). Category 4, Assessment D2

**Fig. 152:**
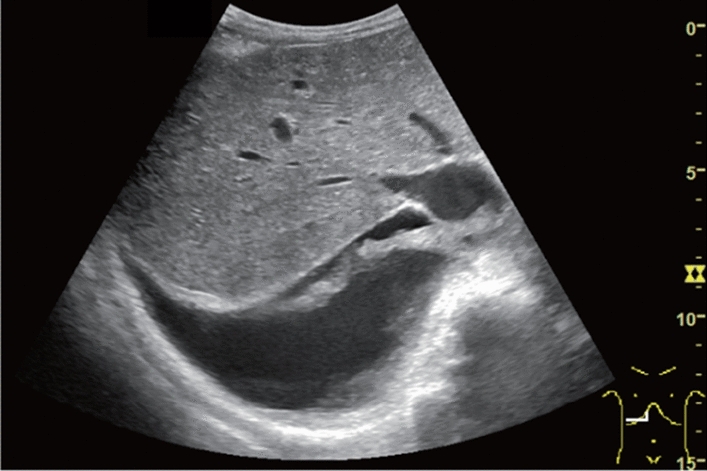
Intrathoracic fluid retention (without debris echo in pleural fluid). Category 3, Assessment D2

**Fig. 153:**
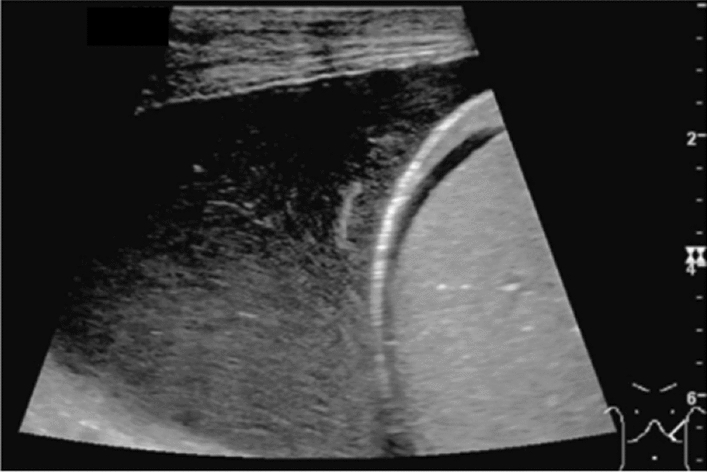
Intrathoracic fluid retention (with debris echo in pleural fluid). Category 4, Assessment D2

**Fig. 154:**
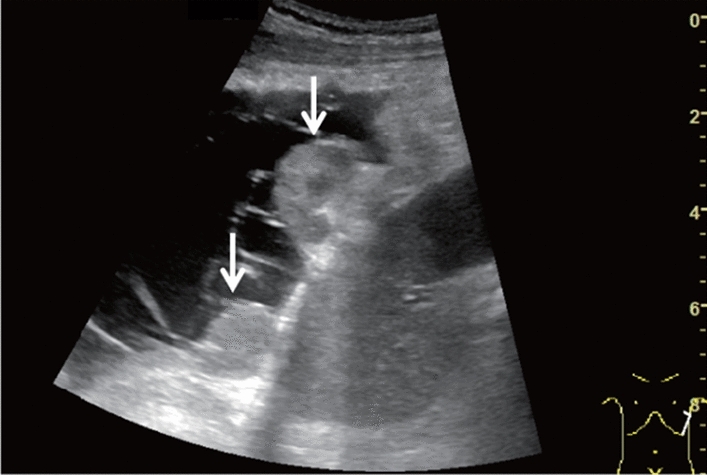
Intrathoracic fluid retention (with solid echo image in pleural fluid). Category 4, Assessment D2

**Fig. 155:**
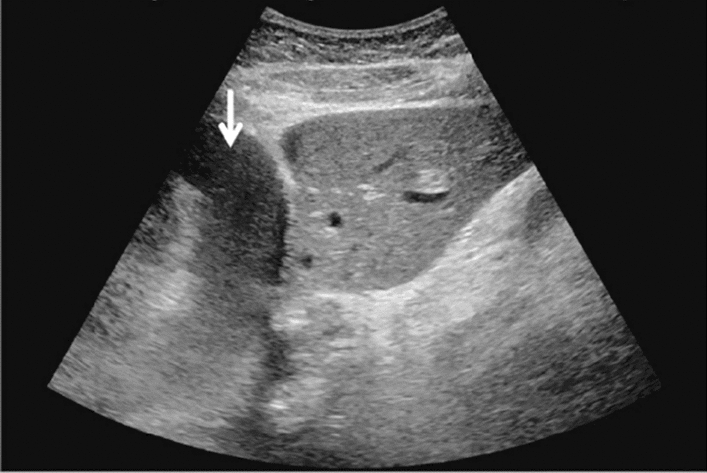
Fluid retention in cardiac cavity. Category 2, Assessment D2

**Fig. 156:**
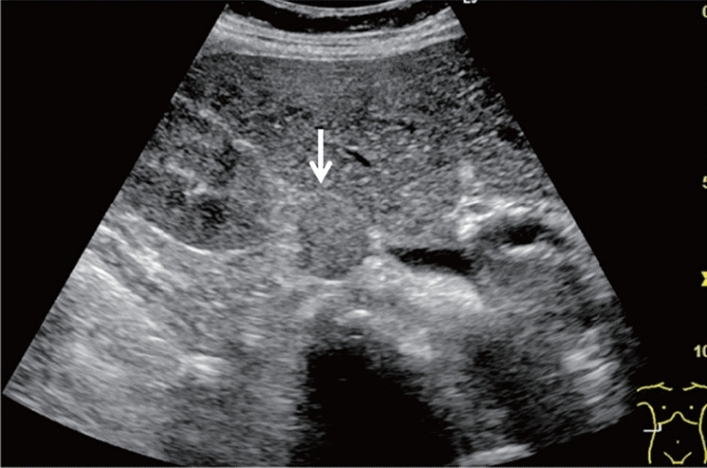
Abdominal cavity, retroperitoneum, or pelvic cavity (including adrenal gland). Retroperitoneal mass image. Category 3, Assessment D2

**Fig. 157:**
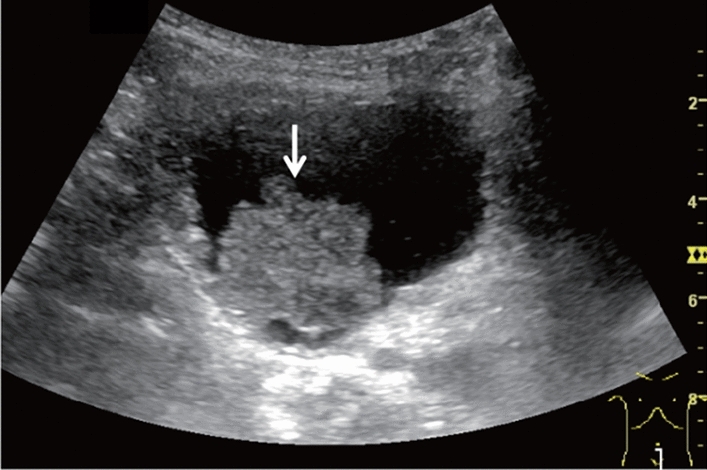
Abdominal cavity, retroperitoneum, or pelvic cavity (including adrenal gland). Intravesical mass image. Category 3, Assessment D2

## Data Availability

The author guideline of JOMU does not require Data availability statement. Also, this article is guideline and there are no data generated by the research group.
